# 5‑^*t*^Bu-Thalidomide Is a Selective Molecular Glue Degrader of the Transcription Factor FLT3 Interacting Zinc Finger 1 (FIZ1)

**DOI:** 10.1021/jacs.6c14185

**Published:** 2026-09-02

**Authors:** Patrick J. Brennan, Ruiyang Guo, Alban Ordureau, Sarah E. Singleton, Miranda Li, Fatma Albayrak, Connor H. Chang, Lorena Yu Liao, Charlotte M. Deane, Kendall N. Houk, Michael M. Hann, Lewis L. Brayshaw, Heeseon An, Stuart J. Conway

**Affiliations:** † Department of Chemistry & Biochemistry, 8783 University of California Los Angeles, Los Angeles, California 90095, United States; ‡ Department of Chemistry, Chemistry Research Laboratory, 6396 University of Oxford, Oxford OX1 3TA, U.K.; § Chemical Biology Program, 5803 Memorial Sloan Kettering Cancer Center, New York, New York 10065, United States; ∥ Tri-Institutional PhD Program of Chemical Biology, Memorial Sloan Kettering Cancer Center, New York, New York 10065, United States; ⊥ Cell Biology Program, Memorial Sloan Kettering Cancer Center, New York, New York 10065, United States; # Department of Chemistry, Gebze Technical University, Kocaeli 41400, Türkiye; ∇ Department of Statistics, University of Oxford, Oxford OX1 3LB, U.K.; ○ GSK, Medicines Research Centre, Stevenage SG1 2NY, U.K.; ◆ California Nano Systems Institute, University of California Los Angeles, Los Angeles, California 90095, United States; ¶ Molecular Biology Institute, University of California Los Angeles, Los Angeles, California 90095, United States; †† Jonsson Comprehensive Cancer Center, University of California Los Angeles, Los Angeles, California 90095, United States

## Abstract

Cys2-His2 (C2H2) zinc finger (ZF)-containing proteins are the largest family of human DNA-binding transcription factors (TFs), a class of proteins that has long been considered undruggable. Significant progress has recently been made in the development of immunomodulatory imide drugs (IMiDs) that induce the degradation of proteins that possess the G-loop CXXCG degron, including these transcription factors. Despite these advances, the identification of IMiD-based molecular glues that selectively induce the degradation of a single protein, and the rational design of such compounds, remains challenging. Here we report the serendipitous discovery that 5-^*t*^Bu-thalidomide (^*t*^Bu-Thal, **5**) potently and selectively induces proteasomal degradation of a single C2H2 ZF-containing TF, FLT3 interacting zinc finger 1 (FIZ1), in six cell lines. While FIZ1 contains 11 C2H2 ZFs, six of which contain the G-loop CXXCG degron necessary for IMiD-induced degradation, we demonstrate that this degradation is mediated exclusively by ZF8. Computational modeling of the CRL4^CRBN^-IMiD-FIZ1 complex enabled rationalization of why ^*t*^Bu-Thal (**5**) selectively engages with ZF8, and hints at a strategy for more rational design of selective IMiD-based degraders. ^*t*^Bu-Thal (**5**) was employed in ChIP-seq studies to identify a motif matching the CRX binding site, consistent with prior reports. We also identified enriched motifs resembling binding sites of the transcription factors ZBTB32 and ARID3a, which have not previously been associated with FIZ1. IP-MS studies indicated that FIZ1 associates with C1QBP and YBX1. C1QBP is involved in DNA damage repair, suggesting a role for FIZ1 in this process. These data show that ^*t*^Bu-Thal (**5**) is a powerful molecular tool to selectively dissect the role of FIZ1 in complex biological settings.

## Graphical abstract

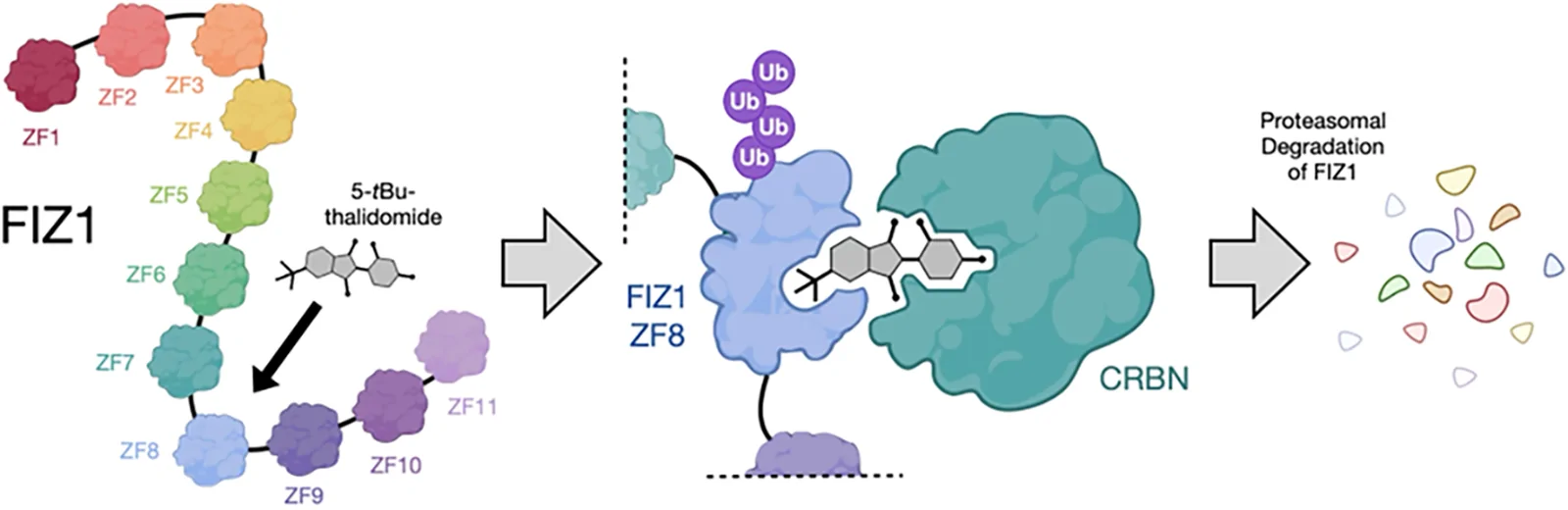

## Introduction

DNA-binding transcription factors (TFs) recognize specific DNA sites and regulate transcriptional output of genes in their immediate vicinity. In doing so, they modulate the identity and fate of cells in multicellular organisms by regulating the common genetic code, and coordinate responses to both internal and external stimuli by functioning as end points in cell signaling networks. There are an estimated >1600 transcription factors (TFs) in the human proteome, and the mutation or dysregulation of at least 300 of these proteins has been linked to human disease.^1^ TFs are, in principle, more attractive as therapeutic targets than upstream proteins such as kinases or G-protein-coupled receptors, as they have the potential for highly selective disease modulation resulting from their ability to regulate a specific set of genes.^2,3^ Despite this, TFs have traditionally been seen as ‘undruggable’ mainly due to the presence of large disordered regions and the lack of well-defined small molecule binding sites.^3^

Cys2-His2 (C2H2) zinc finger (ZF)-containing proteins are the largest family of human DNA-binding TFs. They are characterized by the C2H2 motif, which coordinates to a Zn^2+^ ion that creates the structural integrity of the DNA-binding ‘fingers’. Sequence variation in the ZF enables the recognition of specific DNA sequences.^4^ Interest in this class of TFs as therapeutic targets was sparked when it was shown that the immunomodulatory imide drugs (IMiDs, also known as cereblon E3 ligase modulators [CELMoDs]), pomalidomide (**2**) and lenalidomide (**3**) (Figure 1A), bind to the CUL4·RBX1·DDB1 (CRL4) E3 ligase adaptor protein cereblon (CRBN)^5^ and function through stabilization of a complex between the C2H2 zinc finger-containing proteins IKZF1 (Ikaros) and IKZF3 (Aiolos) and CRBN (Figure 1B).^6,7^ This proximity induction leads to ubiquitination and proteasomal degradation of IKZF1 and IKZF3, which are known as neosubstrates of CRBN. Shortly after this, it was shown that the teratogenic effects of thalidomide (**1**) result from CRBN-induced degradation of the C2H2-containing protein spalt-like transcription factor 4 (SALL4).^8,9^ Since then, there has been significant progress in the development of a more potent and selective degrader for IKZF1/3,^10^ and IMiDs that induce degradation of other C2H2-containing ZFs, including IKZF2 (Helios),^11,12^ WEE1,^13^ WIZ,^14,15^ ZBTB11,^16^ and ZBTB16.^17^ In addition, the scope of proteins that are known to be recruited to CRBN by molecular glues has expanded,^18–20^ and now includes non-C2H2 ZF-containing proteins such as ZMYM2,^21^ and proteins that contain a β-hairpin motif, but not as part of a ZF, including CK1α,^22^ GSPT1,^23,24^ and NEK7.^25^

**Figure 1:**
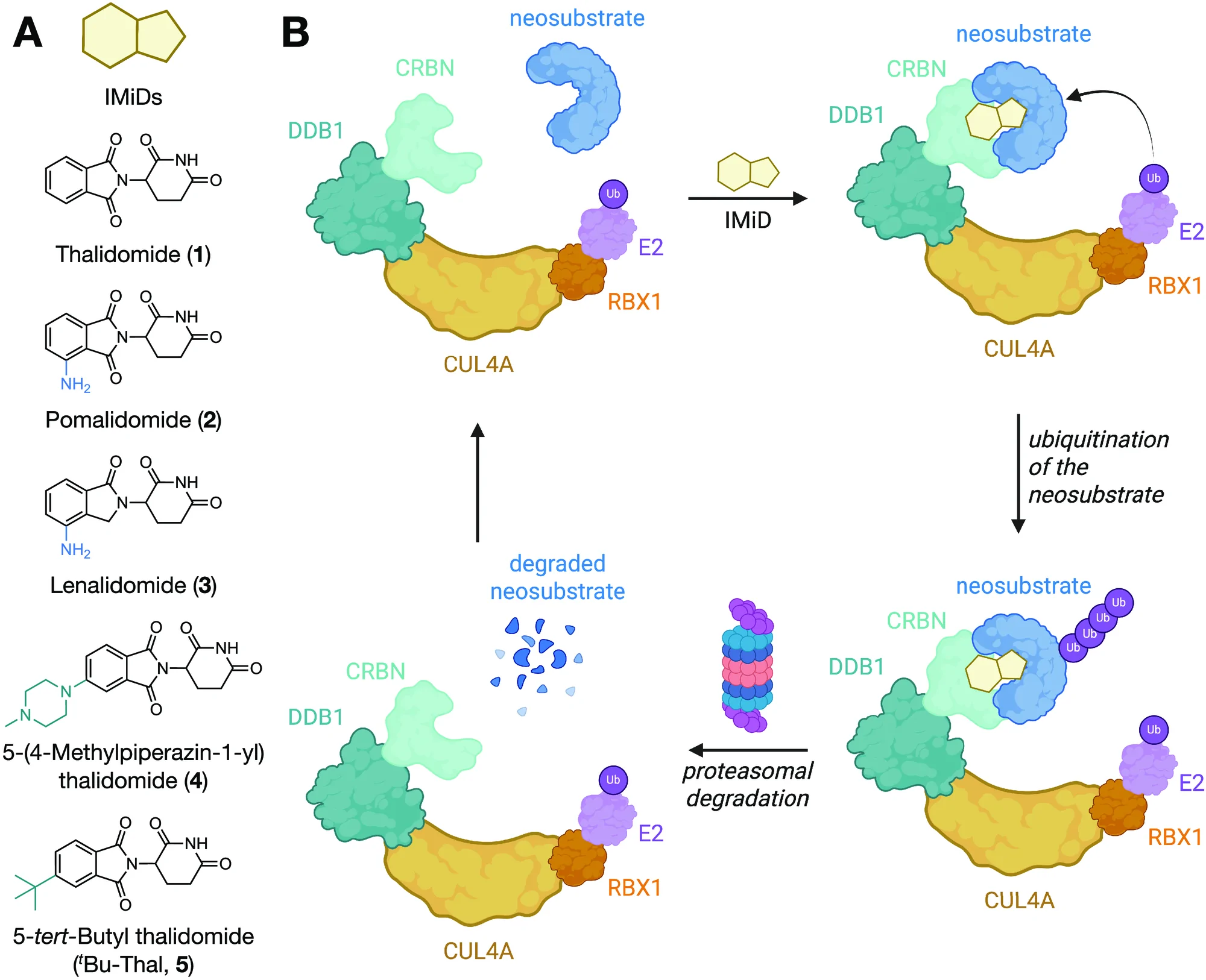
(A) The chemical structures of the common IMiD molecular glues (**1–3**) and the previously reported IMiD analogs used in this study (**4–5**).^36^ (B) Cartoon representation of CRBN-mediated polyubiquitination and subsequent proteasomal degradation of CXXCG-degron-containing neosubstrates induced by the molecular glue action of immunomodulatory drugs (IMiDs).

The work summarized above demonstrates that it is now possible to identify small molecules that induce degradation of C2H2- or β-hairpin-containing proteins. A key challenge that remains, however, is identifying molecules that selectively induce the degradation of a single target in preference to the many hundreds of closely related proteins. In addition, while some trends are starting to emerge, a rational approach to the development of selective IMiD molecular glues has not yet been achieved. We now report the serendipitous discovery that 5-^*t*^Bu-thalidomide (^*t*^Bu-Thal, **5**) potently and selectively induces proteasomal degradation of the C2H2 ZF-containing TF FLT3 interacting zinc finger 1 (FIZ1), in six cell lines. While FIZ1 contains 11 C2H2 ZFs, six of which contain the G-loop CXXCG degron necessary for IMiD-induced degradation,^26^ we demonstrate that this degradation is mediated exclusively by ZF8. Computational modeling of the CRL4^CRBN^-IMiD-FIZ1 complex enabled rationalization of how ^*t*^Bu-Thal (**5**) selectively engages with ZF8 over other FIZ1 ZFs, and hints at a strategy for future rational design of selective IMiD-based degraders.

FIZ1 is widely expressed throughout the body, but is particularly abundant in mature retinal cells.^27–29^ It interacts with neural retina-specific leucine zipper protein (NRL), a transcriptional activator, and cone-rod homeobox protein (CRX), which is a TF affecting the expression of rod-specific genes.^27,30–34^ In addition, FIZ1 has been implicated in the regulation of autocrine signaling in a human keratinocyte cell line^35^ and has been reported to interact with FMS-related tyrosine receptor kinase 3 (FLT3).^31,32^ Despite these studies, the biological roles of FIZ1 remain largely uncharacterized and the potent and selective degrader of FIZ1 reported here will likely be a useful tool for investigations into the role of this protein. This tool will be particularly valuable when the use of gene editing to disrupt the expression of FIZ1 is intractable.

## Results and Discussion

In recent work we took a “bump-and-hole” approach^37^ to identify IMiD analogues that can induce CRBN-mediated degradation of unnatural mutant degrons based on IKZF1/3 ZF2.^36^ We found that changing residues at positions 147, 149, 150, and 154 of IKZF1/3 ZF2 most affected which IMiD analogs were accommodated by the mutant ZF in a ternary complex with CRBN. We showed that the 5-*N*-methylpiperazine derivative of thalidomide (**4**, Figure 1B) induced degradation of a ZF with the residues W, E, I, and S at positions 147, 149, 150 and 154, respectively. Similarly, ^*t*^Bu-Thal (**5**) induced degradation of unnatural ZFs with residues H, E, I, P, or A, E, V, K at positions 147, 149, 150, and 154. Global proteomic analysis in Jurkat (T-lymphoblasts), human embryonic stem cells (ESC), and MM.1S (multiple myeloma) cell lines revealed that **4** did not induce significant degradation of any naturally occurring ZFs expressed in these cell lines. Global proteomic analysis was also conducted to examine the impact of ^*t*^Bu-Thal (**5**) across the same cell lines. Tandem-mass-tag-based proteomics assays in Jurkat cells and ESCs quantified over 9,500 proteins, covering more than 80% of the proteome in the given cell lines without missing values (Table S1). Remarkably, treatment with ^*t*^Bu-Thal (**5**) (16 h) revealed a potent and selective reduction in levels of a single protein, FIZ1 (Figure 2A,2B), in both cell lines.^38^

**Figure 2:**
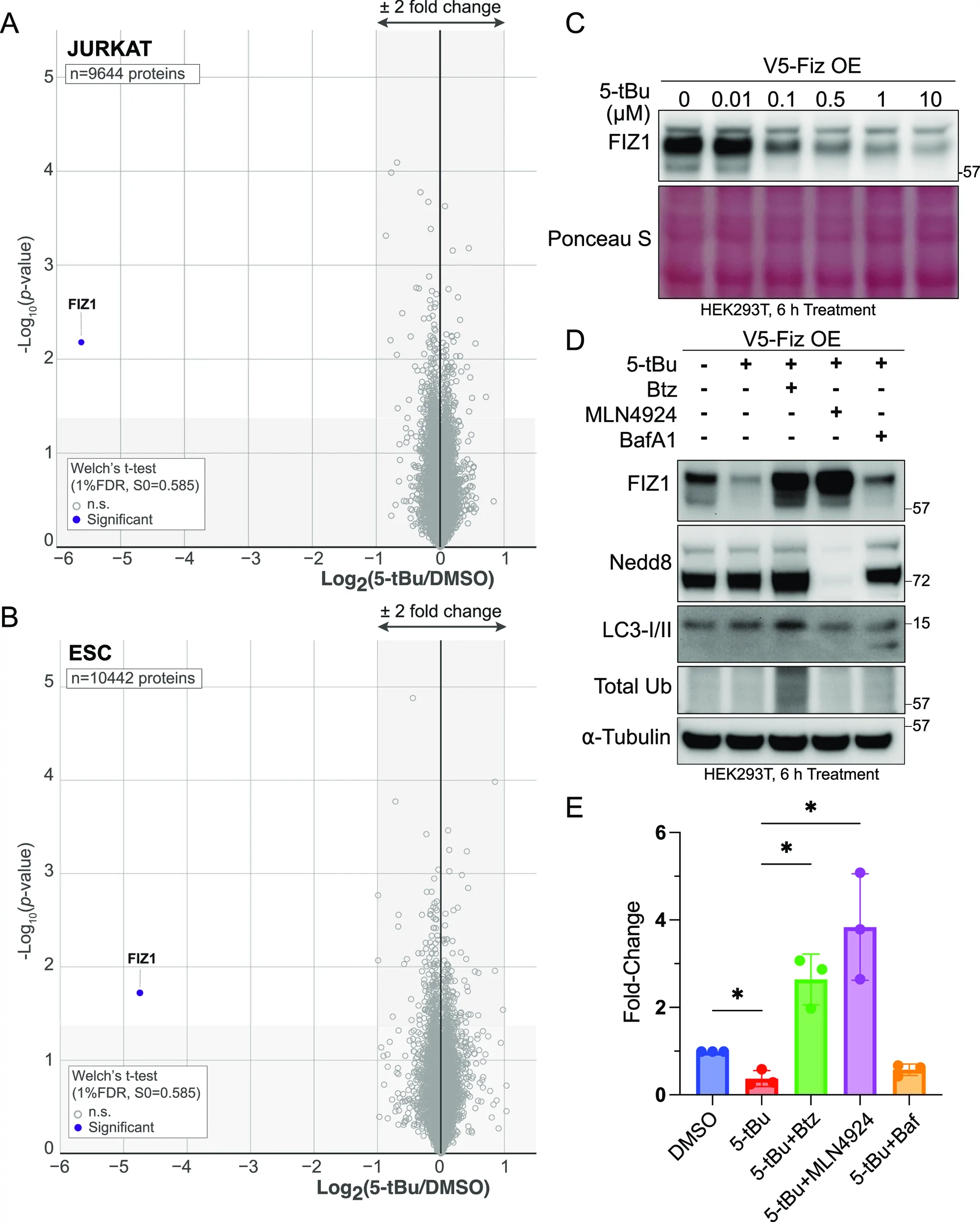
Quantitative proteomics profiling: Jurkat cells (A) or human embryonic stem cells (B) were treated for 16 h with either 5-^*t*^Bu-Thal (**5**, 10 μM) or a DMSO control. Protein abundance was analyzed using multiplexed TMTpro quantification mass spectrometry. log_2_FC is shown on the *x*-axis, and −log_10_(*p*-value) is shown on the *y*-axis. Values shown are the mean of three biological replicates. (C) HEK293T cells were transduced to overexpress V5-tagged FIZ1 and treated with a concentration curve of 5-^*t*^Bu-Thal (**5**, 10 nM–10 μM) for 6 h. One representative blot of three biological replicates. (D, E) HEK293T cells were transduced to overexpress FIZ1 and treated with 5-^*t*^Bu-Thal (**5**, 1 μM) and cotreated with bortezomib (5 μM), MLN4924 (500 nM), or bafilomycin A1 (100 nM) for 6 h. One representative blot of three biological replicates *p < 0.05.

Further proteomics carried out in MM.1S cells confirmed there were no significant proteome changes caused by ^*t*^Bu-Thal (**5**), although FIZ1 was not quantified, which we attribute to low expression levels in this cell line (Figure S1). These results are consistent with a previous report of ^*t*^Bu-Thal (**5**) by Burslem et al., who showed that this compound binds to CRBN with a *K*_d_ = 221 ± 52 nM but did not induce degradation of IKZF3 or CK1α.^38^

Degradation of FIZ1 by ^*t*^Bu-Thal (**5**) was further confirmed by Western blot analysis in an HEK293T cell line overexpressing V5-tagged FIZ1 (Figure 2C). Treatment of this cell line with ^*t*^Bu-Thal (**5**, 1 μM) for 6 h efficiently induced degradation of V5-tagged FIZ1, while coincubation with ^*t*^Bu-Thal (**5**, 1 μM) and either the proteasome inhibitor bortezomib (5 μM),^39^ or the neddylation inhibitor MLN4924 (500 nM),^40^ rescued this degradation (Figure 2D,E). This result is consistent with degradation of FIZ1 proceeding through the proteasome following ubiquitination by a Cullin-RING E3 ligase. Co-incubation of HEK293T cell line overexpressing V5-tagged FIZ1 with ^*t*^Bu-Thal (**5**, 1 μM) and bafilomycin A1 (100 nM)^41^ for 6 h also resulted in efficient degradation of V5-tagged FIZ1 (Figure 2D,E), suggesting that lysosomal protein degradation is not involved in ^*t*^Bu-Thal (**5**)-induced FIZ1 degradation. This result is consistent with the established mechanism by which IMiD-based compounds induce degradation of their targets through recruitment of CRBN.^5^

Prior studies have shown that CRBN is located in both the cytoplasm and the nucleus,^42^ and its neosubstrates are found in multiple cell compartments, including the nucleus (e.g., IKZF1/Ikaros)^43,44^ and cytosolic vesicles (e.g., CK1α).^45^ Research on FIZ1′s localization have been somewhat inconsistent, with the initial study demonstrating its equal distribution between nucleus and cytoplasm in a fibroblast cell line, NIH3T3, leading to the hypothesis that the cytoplasmic pool of FIZ1 may interact with FLT3, a receptor tyrosine kinase expressed on the plasma membrane.^31^ Another study showed that FIZ1 is predominantly present in the nucleus, with a minor population detected in the cytoplasm in a keratinocyte line, HaCaT cells.^35^ Our nuclear-cytosolic fractionation and live-cell imaging data suggest that FIZ1 is predominantly localized in the nucleus in HEK293T (embryonic kidney) and HCT116 (colon cancer) cells (Figure 3A,3B).^35^ Treatment of HCT116 cells stably expressing mCherry-FIZ1 fusion with ^*t*^Bu-Thal (**5**) showed a dose-dependent loss of mCherry signal, which was rescued by treatment with MLN4924, when analyzed using flow cytometry (Figure 3C,D). These data provide further evidence that ^*t*^Bu-Thal (**5**) induces CRBN-mediated degradation of FIZ1, as observed in the ESCs and Jurkat cell lines.

**Figure 3:**
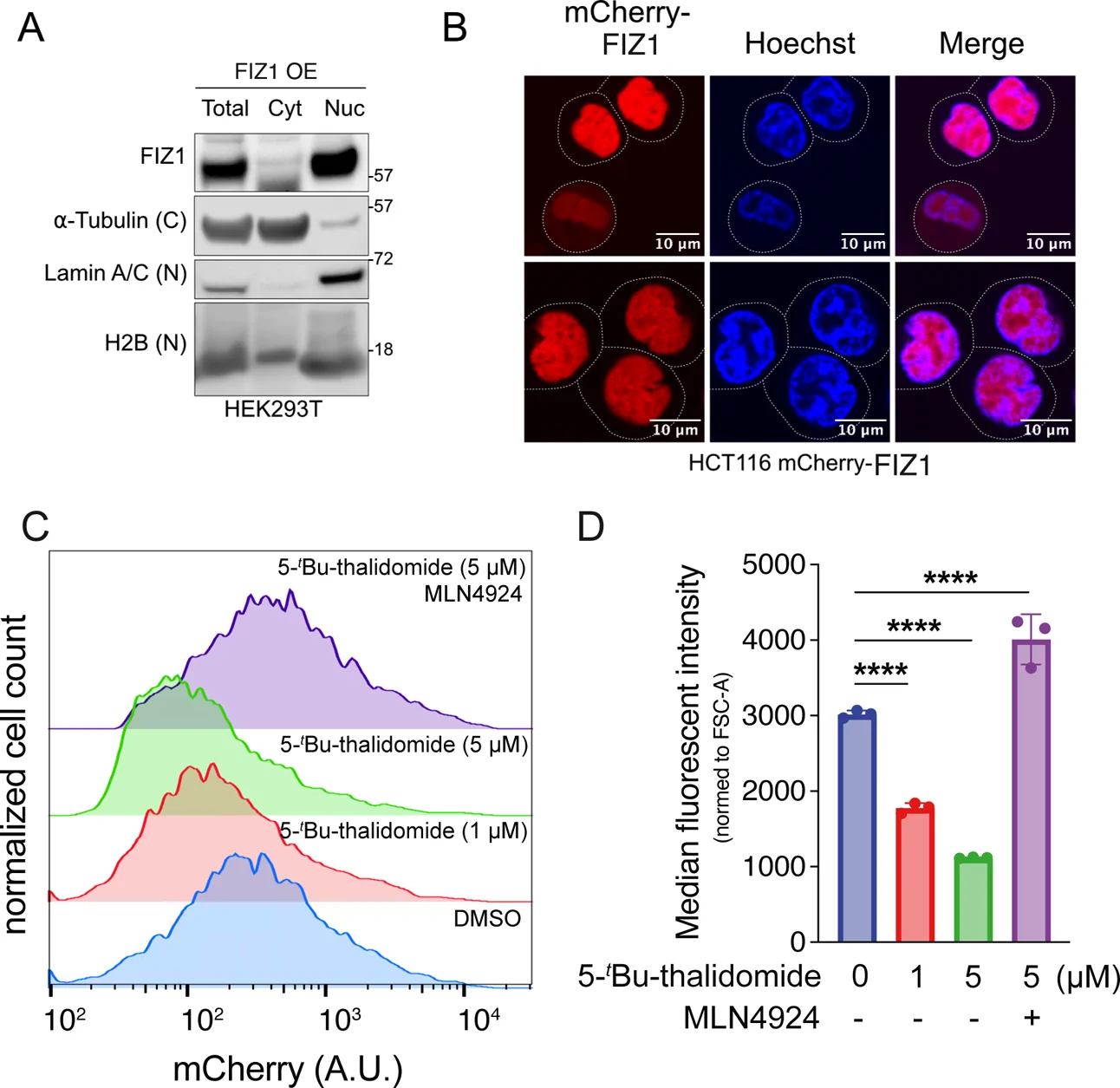
(A) Nuclear-cytosolic fractionation of HEK293T cells stably transduced with FIZ1 shows localization of overexpressed FIZ1 to the nucleus by colocalization with nuclear markers (laminA/C and Histone H2B) and lack of colocalization with a cytosolic marker (α-tubulin). (B) Imaging analysis using the HCT116 cell line demonstrates nuclear localization of overexpressed mCherry-FIZ1 by colocalization with Hoechst stain. (C, D) HCT116 cells stably expressing mCherry-FIZ1 were treated with DMSO, 5-^*t*^Bu-Thal (**5**, 1 μM or 5 μM), or 5-^*t*^Bu-Thal (**5**, 5 μM) and MLN4924 (500 nM) for 16 h, then analyzed using flow cytometry for changes in mCherry signal. The representative line histogram is shown in panel (C), while the mean ± s.d. of biological triplicates is presented as a bar graph in panel D. The mCherry signal in panel (D) was normalized by forward scatter-area (FSC-A) to account for cell size changes upon MLN4924 treatment.

FIZ1 contains 11 C2H2 ZFs (Figure 4A);^31^ analysis of the primary sequence (Figure 4B) indicates that these ZFs are found in four clusters, comprising ZF1–4, ZF5 and 6, ZF7 and 8, and ZF9–11 (Figure 4A,C). Six ZFs, ZF1, 2, 7, 8, 10, and 11, possess the characteristic conserved glycine residue found in the CXXCG degron motif required for IMiD analog-induced degradation (Figure 4B). While the majority of the FIZ1 ZFs possess the typical number of 23 amino acid residues, ZFs 4, 7, and 8 are 24, 22, and 24 residues in length, respectively, due to either an extra residue between the two C2H2 histidine residues (ZF4 and ZF8), or one less amino acid between the two C2H2 cysteine residues (ZF7).^16^

**Figure 4:**
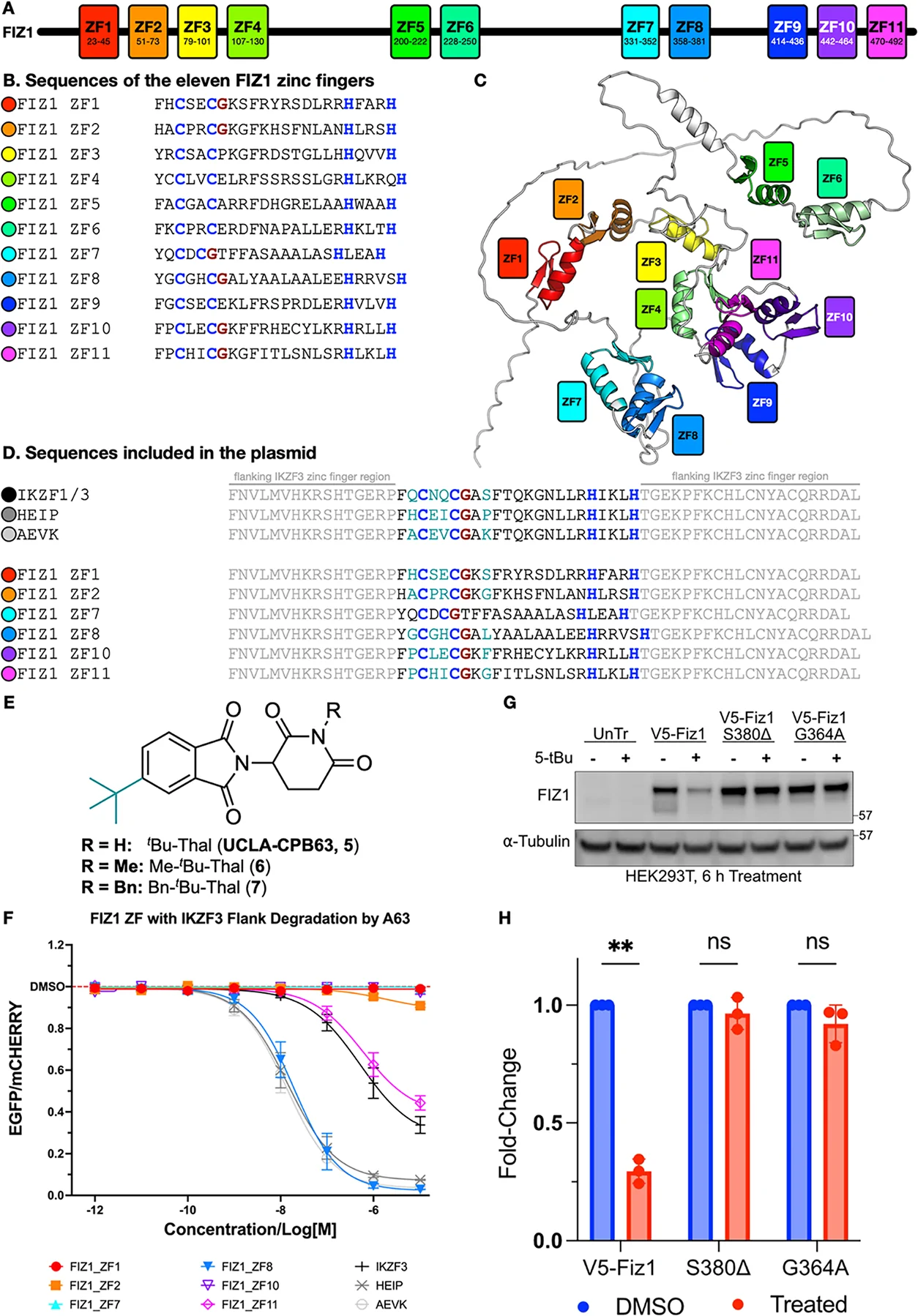
(A) The domain structure of FIZ1. (B) The sequences of the 11 ZFs found in FIZ1, the C2H2 motif is highlighted in blue, and the glycine found in the CXXCG degron is shown in maroon. (C) The AlphaFold 3-predicted structure of FIZ1 with the ZFs highlighted. (D) The sequences of the six FIZ1 ZFs (1, 2, 7, 8, 10, and 11) and the control ZFs (IKZF1/3 ZF2, the HEIP and AEVK degrons) that were incorporated into the Artichoke plasmid for the flow cytometry assay. The C2H2 motif is highlighted in blue, the glycine found in the CXXCG degron is shown in maroon, and the four interface residues are shown in turquoise. (E) The chemical structures of ^*t*^Bu-Thal (UCLA-CPB63, **5**), Me-^*t*^Bu-Thal (**6**), and Bn-^*t*^Bu-Thal (**7**). (F) Normalized eGFP/mCherry fluorescence ratio for ZF-EGFP constructs stably expressed in Jurkat cells after 18 h treatment with a concentration curve of **5** (1 pM–10 μM). (G) HEK293T cells overexpressing V5-tagged FIZ1—either WT, S380Δ, or G364A variants—alongside untransduced HEK293T cells, were either treated with **5** (**1** μM) or a DMSO control for 6 h. (H) Quantification of the data shown in panel G. Mean ± s.d. *n* = 3 biological replicates. **p < 0.01.

We were interested whether a single ZF was responsible for mediating the ^*t*^Bu-Thal (**5**)-induced degradation of FIZ1, or whether multiple ZFs can engage with this compound (**5**). To investigate this, all six CXXCG-containing ZFs were expressed individually as part of a dual reporter system, in which the ZF-EGFP fusion protein is expressed simultaneously with mCherry, but separated by an internal ribosomal entry site (IRES) in the Artichoke plasmid (Addgene #73320; Figure S2). When IMiD treatment results in ZF-EGFP degradation, a change in the EGFP to mCherry ratio is observed using flow cytometry, allowing quantitative analysis of protein degradation.^26,36^

In our previous work we used this assay to study IKZF1/3 ZF2-based mutant degrons, employing a 60 amino acid construct in which the 23 amino acid ZF sequence was flanked by additional residues from IKZF3 (16 residues upstream and 21 residues downstream).^36^ Using this approach we observed efficient degradation of the EGFP fusion proteins, and so we employed this system here by evaluating the degradation of each FIZ1 ZF while flanked by the IKZF3-derived residues. This approach ensures that differences in degradation observed are due to the variance in the 23 residue ZF motif, and not due to differences between the regions upstream and downstream of the ZF. As ZFs 4, 7, and 8 have 22 or 24 amino acids, rather than 23, a number of sequence designs that adjusted these sequences to have 23 amino acids were investigated, including an S380Δ mutation in ZF8 (Figure S3), but ultimately, it primarily was the wild-type sequences, shown in Figure 4D, that provided the most insight into the molecular basis of ^*t*^Bu-Thal (**5**)-induced FIZ1 degradation.

Upon treatment with ^*t*^Bu-Thal (**5**), only FIZ1 ZF8 showed substantial degradation (Figure 4F). This ZF was degraded to a similar extent as the unnatural HEIP and AEVK degrons, which we previously developed to engage with ^*t*^Bu-Thal (**5**) (*vide supra*).^36^ Interestingly, treatment with ^*t*^Bu-Thal (**5**) did not induce degradation of the S380Δ mutant of ZF8 (Figure S3). ZF11 showed modest degradation, at a level similar to that observed for IKZF3 ZF2. Previous work has shown that this assay is very effective at detecting degradation, and that less pronounced degradation in the assay does not always translate into degradation of the corresponding full-length protein. So, although we also observed modest ^*t*^Bu-Thal (**5**)-induced degradation of IKZF3 ZF2 in the assay, we have previously shown that this compound does not induce degradation of IKZF3 in cells.^36^ Based on these previous results, we proposed that ZF8 and not ZF11 plays the primary role in mediating ^*t*^Bu-Thal (**5**)-induced degradation of FIZ1. Coincubation with either the neddylation inhibitor MLN4924 ^40^ or the proteasome inhibitor carfilzomib^47^ rescued degradation of the ZF8 construct, consistent with reliance on both ubiquitination and proteasomal degradation (Figure S4). To further confirm that FIZ1 degradation proceeds via interaction with CRBN, we synthesized the *N-*methyl (**6**, Figure 4E) and *N-*benzyl (**7**, Figure 4E) derivatives of ^*t*^Bu-Thal (**5**), as previous studies have used *N*-methyl and *N-*benzyl IMiD derivatives as negative control PROTACs and molecular glues.^48–51^ We proposed that these compounds would have reduced or no affinity to CRBN and so would not induce degradation of FIZ1 through the canonical IMiD pathway. To test this idea, we treated FIZ1 ZF8-EGFP- and mCherry-expressing Jurkat cells with ^*t*^Bu-Thal (**5**), Me-^*t*^Bu-Thal (**6**), or Bn-^*t*^Bu-Thal (**7**) and measured their effect on EGFP levels. As expected, ^*t*^Bu-Thal (**5**) induced effective degradation of ZF8-EGFP consistent with the data described above, while Me-^*t*^Bu-Thal (**6**) induced only very modest degradation of ZF8-EGFP and Bn-^*t*^Bu-Thal (**7**) induced no observable degradation of ZF8-EGFP in this system (Figure S5).

We next wanted to confirm that ZF8 was primarily responsible for mediating ^*t*^Bu-Thal (**5**)-induced degradation of FIZ1 in the full-length protein. We proposed that a G364A mutation would disrupt the CXXCG degron and therefore should cause FIZ1 to lose its sensitivity to ^*t*^Bu-Thal (**5**). Previous studies have reported that mutation of the β-hairpin glycine to alanine abolishes IMiD-induced degradation in other ZFs.^52^ We also hypothesized that the S380Δ*m*utant of FIZ1 would also be insensitive to ^*t*^Bu-Thal (**5**)-induced degradation, given its lack of degradation in the flow cytometry assay (*vide supra*). Therefore, WT, S380Δ, and G364A versions of V5-tagged FIZ1 were overexpressed in HEK293T cells, which were then treated with either ^*t*^Bu-Thal (**5**) (1 μM for 6 h) or a DMSO control (Figure 4G, H). This resulted in effective degradation of the WT FIZ1 protein, while both the S380Δ, and G364A mutants were unaffected by treatment with ^*t*^Bu-Thal (**5**). These data demonstrate that ZF8 is solely responsible for mediating ^*t*^Bu-Thal (**5**)-induced degradation of FIZ1 in cells. To investigate the effect of ^*t*^Bu-Thal (**5**) on degrading endogenous (i.e., not overexpressed) levels of FIZ1 we investigated the use of FIZ1 antibodies to directly monitor its levels in HEK293T, HeLa, and U2OS cell lines. Unfortunately, all of the commercial antibodies that we investigated showed nonspecific binding and did not report accurately on FIZ1 degradation (Figure S6). To overcome this problem, we generated new CRISPR knock-in cell lines expressing endogenous FIZ1 C-terminally tagged with HaloTag7-V5 in HCT116, HEK293T, and U2OS cell lines (Figure 5A). Treatment of these cell lines with increasing amounts of ^*t*^Bu-Thal (**5**) (0.1–5.0 μM) led to a concentration-dependent degradation of V5-tagged FIZ1, which was detected using a V5 antibody (Figure 5B). Treatment with the negative control compound Bn-^*t*^Bu-Thal (**7**, 5 μM) led to no observable degradation of V5-tagged FIZ1 (Figure 5B). This result was confirmed using TMT11-plex proteomics of HCT116 cells treated for 6 h with DMSO or Bn-^*t*^Bu-Thal (**7**, 5 μM), where again no depletion of any protein was observed (Figure S7A).

**Figure 5:**
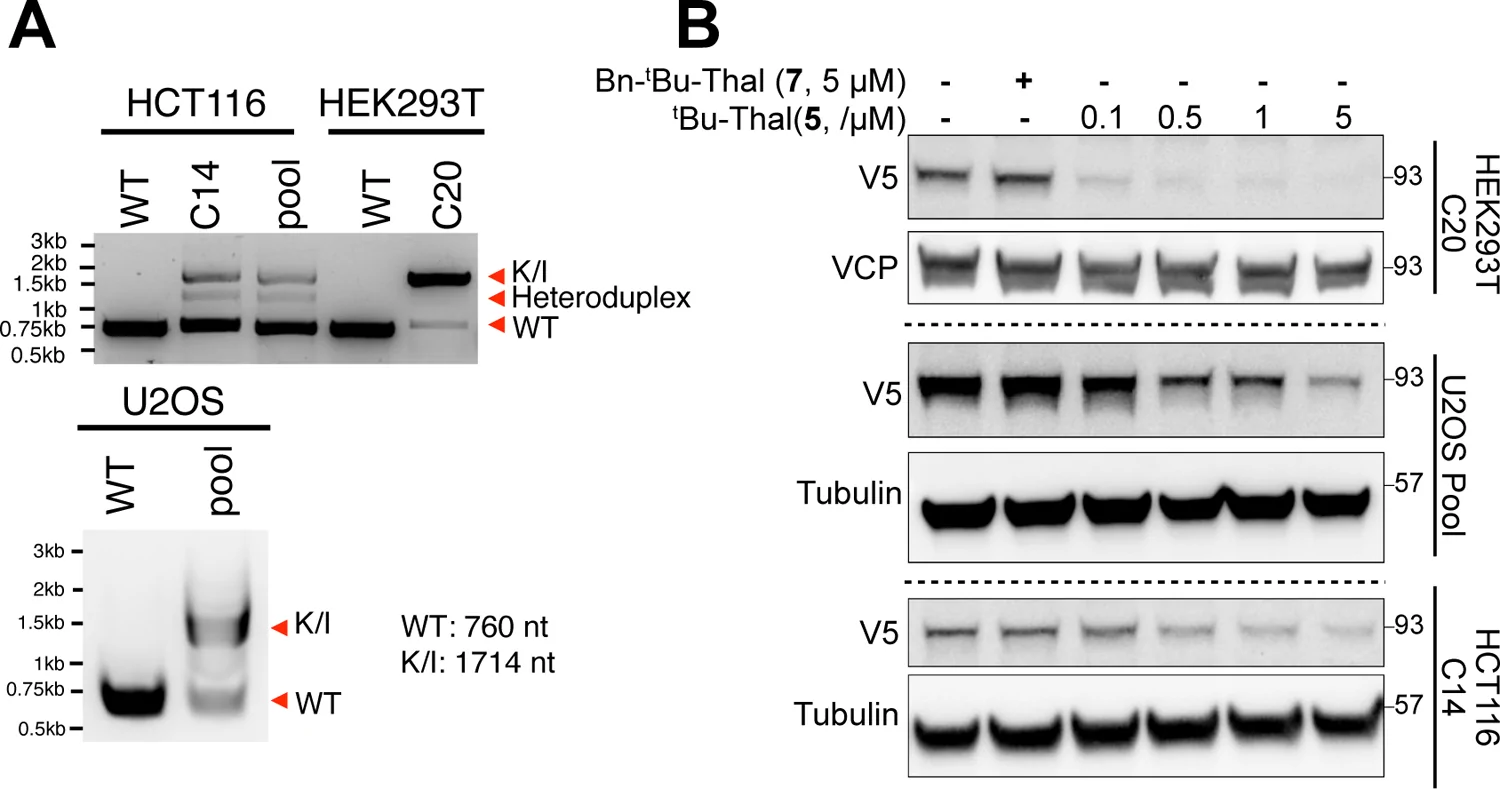
(A) PCR Genotyping of FIZ1-HaloTag-V5 knock-in cell lines. Expected lengths of the FIZ1 wild-type and knock-in amplicons are 760 and 1714 nucleotides, respectively. (B) The indicated cell lines were treated for 6 h with DMSO, Bn-^*t*^Bu-Thal (**7**, 5 μM), or the indicated concentration of ^*t*^Bu-Thal (**5**). One representative blot of three biological replicates.

### Functional Characterization

Having demonstrated that ^*t*^Bu-Thal (**5**) is a tool compound that selectively degrades FIZ1 in a range of cell lines, we decided to use it to investigate the biological function of FIZ1. This protein was first reported as a FLT3 interacting protein,^31^ and so we first sought to investigate whether degradation of FIZ1 impacted the function of FLT3. We assessed the proteome-wide impact of long-term FIZ1 degradation in MOLM13 cells. This is an acute myeloid leukemia cell line that expresses FLT3, a driver of, and one of the most frequently mutated genes in, acute myeloid leukemia, although the physiological function of this interaction remains unknown.^31^ After 72 h, FIZ1 was again the only protein whose change in abundance was statistically significant (Figure S8 and Table S1). The lack of other significant changes in the global proteome suggests that the role of FIZ1 in hematologic malignancies under normal culture conditions may be more subtle, and further study is required to tease out its function in this context.

Our data show that FIZ1 mainly localizes to the nucleus (Figure 3), unlike FLT3, which is a class III receptor tyrosine kinase that localizes to the cell membrane or endoplasmic reticulum.^53^ Therefore, we reasoned that FIZ1 may primarily function as a transcriptional regulator rather than as an interactor of FLT3, and sought to identify the specific chromosomal binding sites at which this TF interacts. We used the HCT116 FIZ1 knock-in cell line (Figure 6A) as our model to apply ^*t*^Bu-Thal (**5**), given its near diploid karyotype.^54^ ChIP-seq comparing FIZ1-HaloTag-V5 knock-in HCT116 cells treated with ^*t*^Bu-Thal (**5**) or Bn-^*t*^Bu-Thal (**7**, 5 μM) (control), showed FIZ1-dependent enrichment of sites, which were depleted upon ^*t*^Bu-Thal (**5**) treatment (Figure 6B). *De novo* motif analysis of these sites recovers a motif matching the CRX binding site, corroborating previous reports linking FIZ1 to photoreceptor maturation and validating our approach (Figure 6C).^27,33^ We note that while this sequence is present and bound by FIZ1, it is possible that it is not transcribed in HCT116 cells. The same analysis identified additional enriched motifs resembling binding sites of transcription factors not previously linked to FIZ1, including the lymphoid regulators ZBTB32 and ARID3a, indicating that FIZ1-bound regions encompass sequence contexts beyond the CRX motif. We note that these *de novo* motifs reflect the sequence composition of FIZ1-bound regions in HCT116 cells rather than direct sequence-specific binding by FIZ1.

**Figure 6:**
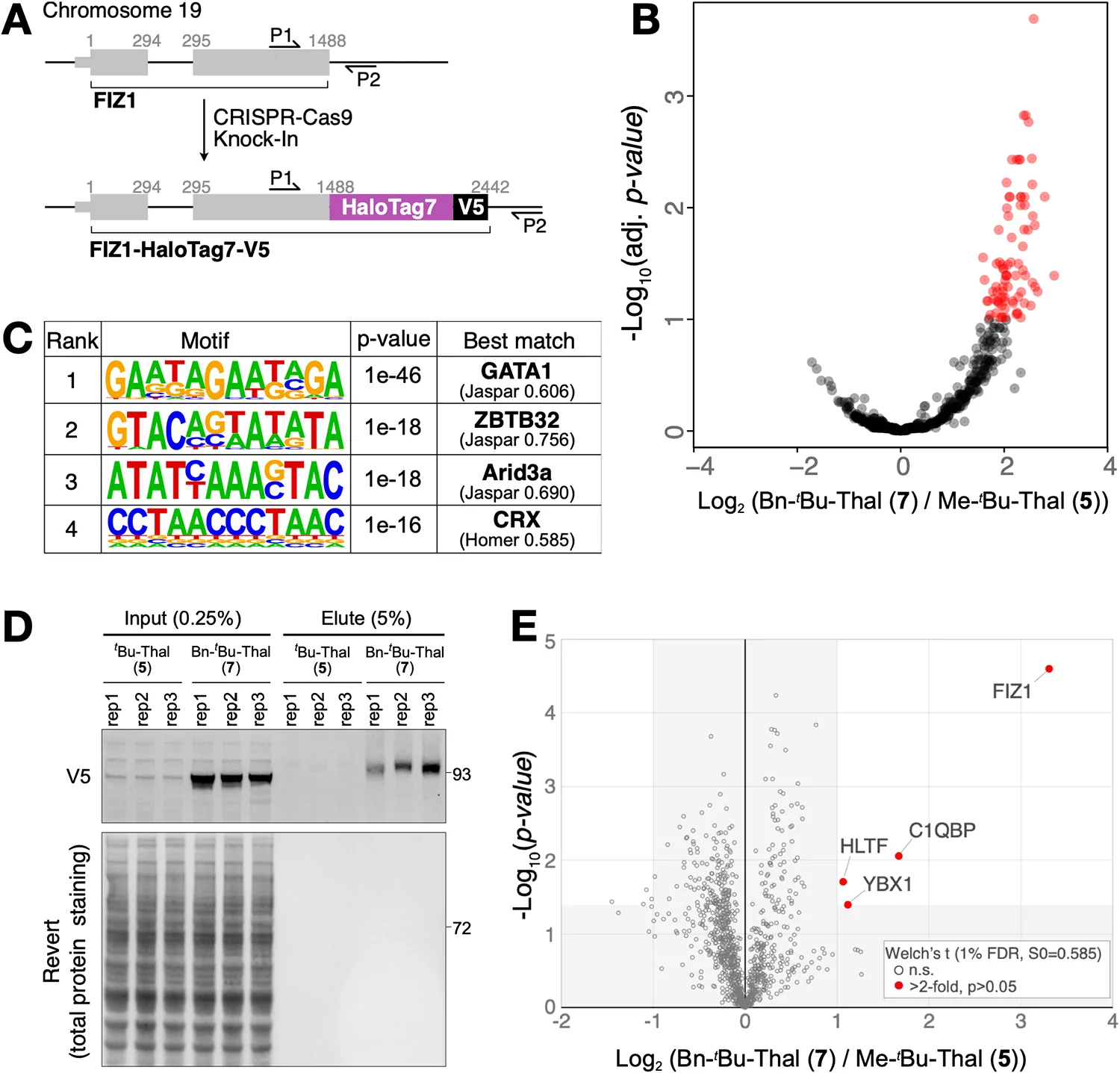
(A) A schematic of the knock-in strategy at the C-terminus of the endogenous FIZ1 locus. (B) Volcano plot of ChIP-seq data comparing peaks in FIZ1-HaloTag-V5 HCT116 cells treated with ^*t*^Bu-Thal (**5**, 5 μM) or Bn-^*t*^Bu-Thal (**7**, 5 μM). Red points indicate peaks with statistically significant increased enrichment. (C) Logos of motifs discovered by *de novo* enrichment analysis of FIZ1 target sequences. (D) Western blot quantification of FIZ1 immunoprecipitation in FIZ1-HaloTag-V5 knock-in HCT116 cells treated with ^*t*^Bu-Thal (**5**, 5 μM) or Bn-^*t*^Bu-Thal (**7**, 5 μM). (E) TMT 6-plex data of anti-V5 immune-purified proteins from FIZ1-HaloTag-V5 HCT116 cells treated with ^*t*^Bu-Thal (**5**, 5 μM) or Bn-^*t*^Bu-Thal (**7**, 5 μM).

At the protein level, prior studies suggest that FIZ1 acts in concert with other transcription factors in a cell-type-specific manner.^27,33^ To probe its interactors in an unbiased manner, we performed immunoprecipitation mass-spectrometry (IP-MS) with this same FIZ1-HaloTag-V5 knock-in HCT116 cell line, comparing cells treated with the inactive Bn-^*t*^Bu-Thal (**7**, 5 μM) control against cells treated with ^*t*^Bu-Thal (**5**). Western blot analysis confirmed almost complete degradation of FIZ1 upon ^*t*^Bu-Thal (**5**) treatment (Figure 6D), with V5-tagged FIZ1 enriched only in the control compound-treated samples (Figure 6D). As the two conditions differ primarily in the presence of FIZ1, the degrader-treated sample serves as an isogenic, FIZ1-deleted background, and proteins enriched in the FIZ1-retained condition can be assigned as FIZ1-dependent interactors. The subsequent IP-MS analysis indicates that complement component 1 Q subcomponent-binding protein (C1QBP), Y-box binding protein 1 (YBX1), and helicase-like transcription factor (HLTF) are enriched more than 2-fold in a FIZ1-dependent manner (Figure 6E), and concomitantly depleted when FIZ1 is absent/degraded. Of these, C1QBP and YBX1 have previously been shown to interact, with C1QBP being involved in DNA damage repair,^55^ placing FIZ1 in a candidate complex with these two factors in HCT116 cells. Consequently, we have shown that FIZ1 interacts with C1QBP and YBX1, suggesting a role in the DNA damage repair response.

### Structure–Activity Studies

To investigate why ZF8 can form a ternary complex with ^*t*^Bu-Thal (**5**) and CRBN, but other FIZ1 ZFs cannot, we generated AlphaFold 3 predicted structures of all 6 FIZ1 ZFs investigated in this study and overlaid them for comparison (Figure 7A). As we have previously seen that the IKZF3 ZF2 residues 147, 149, 150, and 154 have a strong influence on which IMiDs are accommodated in a ZF-IMiD-CRBN ternary complex, we focused on the equivalent residues in the 6 FIZ1 ZFs investigated in this study. Analysis of these residues showed no sequence consensus at these positions (Figure 7B) explaining the selective engagement of ^*t*^Bu-Thal (**5**) with ZF 8. Of all the FIZ1 ZFs investigated, ZF8 possesses the sterically least encumbered residues in the four interface amino acids. Both position 359 (equivalent of IKZF3 position 147) and 361 (equivalent of IKZF3 position 149) are glycine residues, while position 366 (equivalent of IKZF3 position 154) is a flexible leucine residue. To investigate how these residues accommodated ^*t*^Bu-Thal (**5**), we overlaid the AlphaFold 3 ^46^ predicted ZF8 structure with the ZF in the crystal structure of the CRBN-pomalidomide-IKZF1 ZF2 ternary complex (PDB ID: 6H0F).^26^ We replaced the original pomalidomide ligand with ^*t*^Bu-Thal (**5**) in the equivalent pose and ran a 2 ns molecular dynamics (MD) simulation to relax this complex (Figure 7C). The resulting structure indicates that the presence of the two glycine residues at the ZF-CRBN interface provides sufficient room for the bulky ^*t*^Bu group to be accommodated in this region. Conversely, overlaying the residues from the IKZF1 ZF2 ternary complex (PDB ID: 6H0F) demonstrate that the bulky glutamine residue at position 147 (IKZF3 numbering) would clash with the ^*t*^Bu group, indicating why ^*t*^Bu-Thal (**5**) does not induce degradation of IKZF1 or IKZF3 in cells (Figure 8).

**Figure 7:**
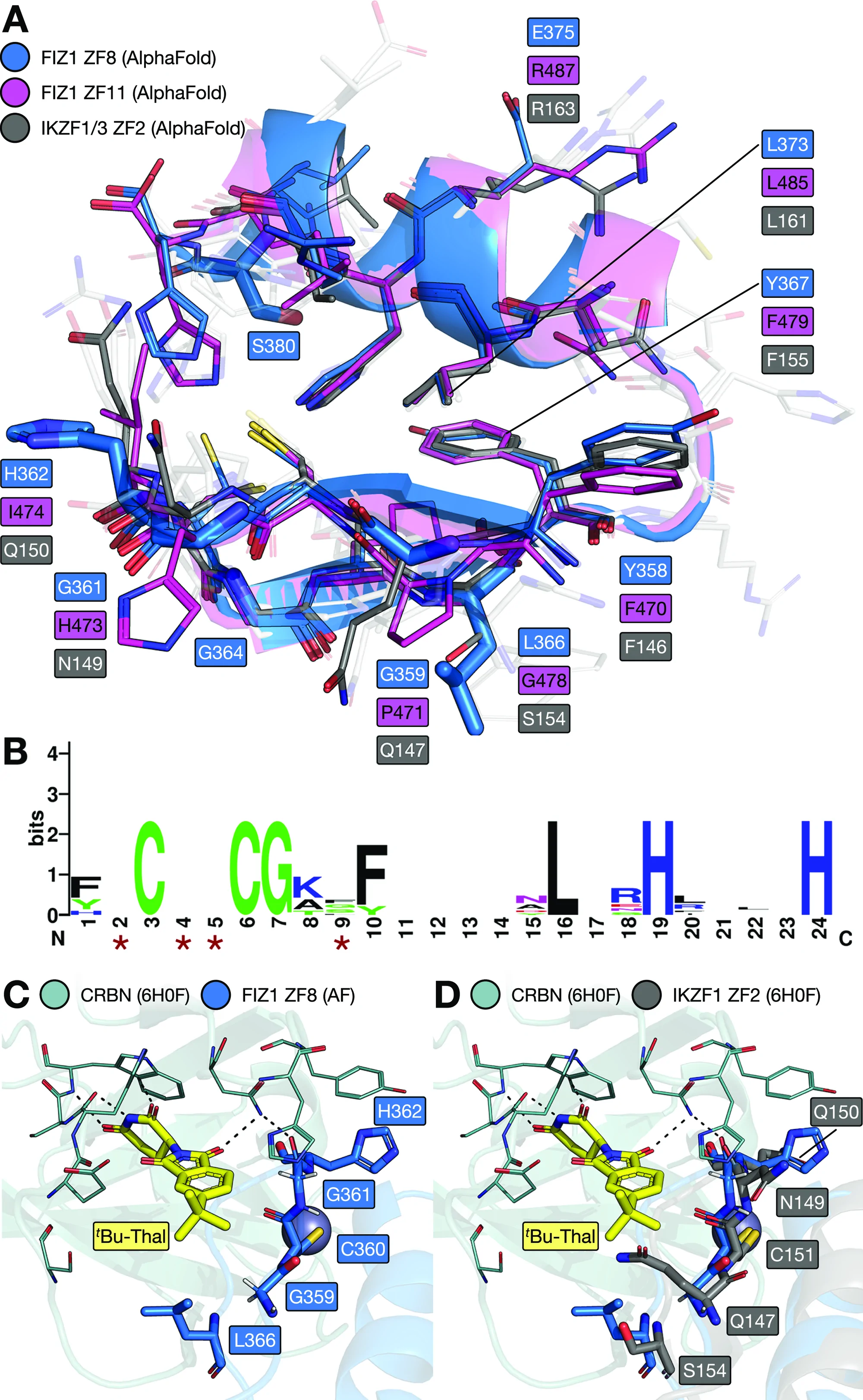
(A) An overlay of the AlphaFold 3-predicted structures of FIZ1 ZF 1, 2, 7, and 10 (carbon = white and transparent), ZF8 (carbon = blue) and ZF11 (carbon = magenta) with the AlphaFold 3-predicted structure of IKZF1/3 (carbon = gray). The residues that form interface with CRBN and conserved residues are indicated. (B) The consensus sequence logo of FIZ1 ZF1, 2, 7, 8, 10, and 11 highlighting that there is no similarity between the CRBN-interfacing residues (red asterisk) of these zinc fingers. (C) A structure predicting the binding mode of ^*t*^Bu-Thal (**5**) to the complex of CRBN (carbon = green) with FIZ1 ZF8 (carbon = blue) resulting from docking and MD simulations using an AlphaFold model of FIZ1 ZF8. The presence of G359 and G361 at this interface are proposed to be responsible for accommodating the bulky ^*t*^Bu group of ^*t*^Bu-Thal (**5**). (D) The overlay of the X-ray crystal structure of IKZF1 ZF2 ternary complex with pomalidomide and CRBN (PDB ID: 6H0F), predicting where Q147 would clash with the ^*t*^Bu group of ^*t*^Bu-Thal (**5**).

**Figure 8:**
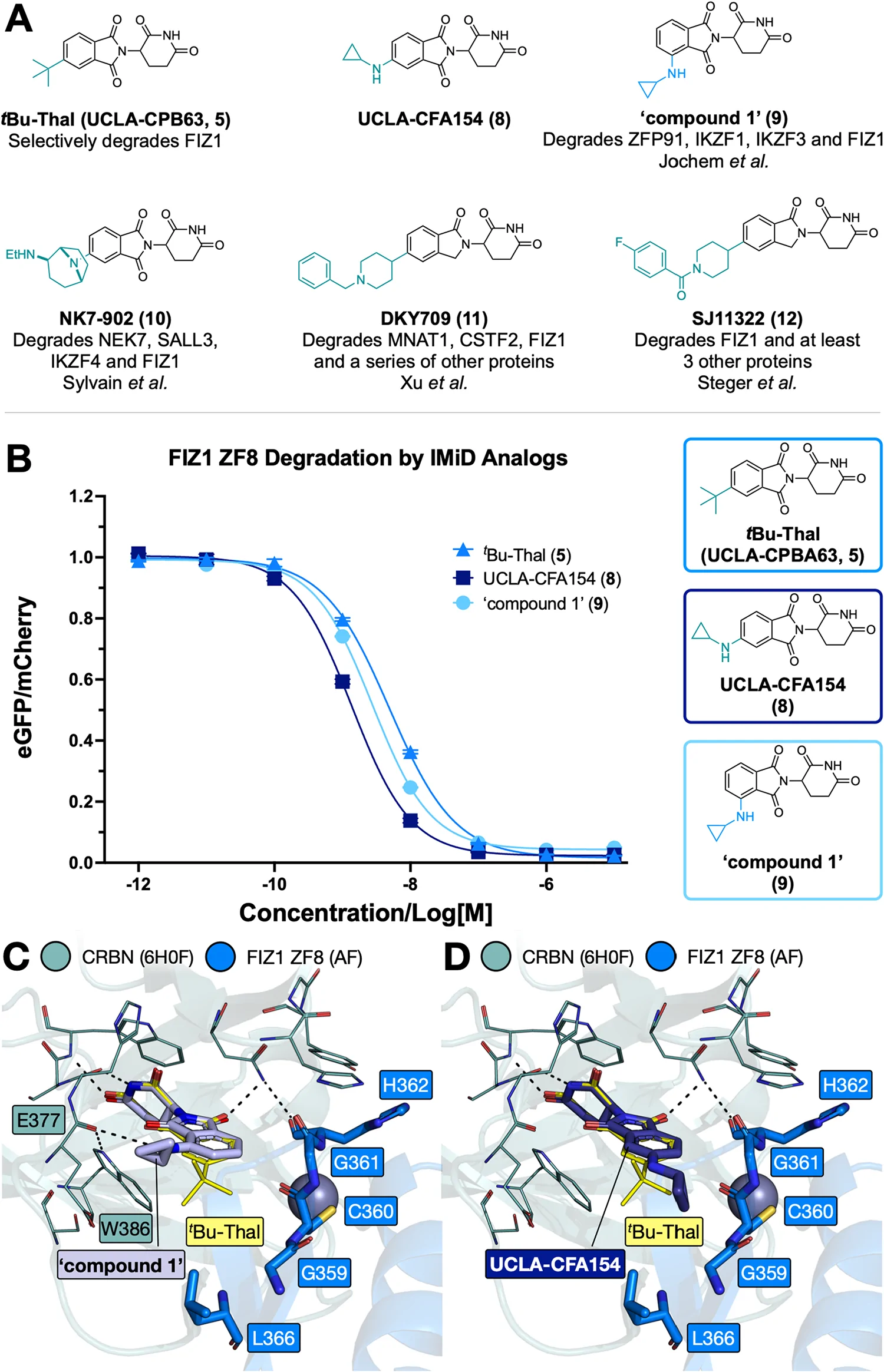
(A) The structures of **5**, **8**, and the currently reported molecular glues that induce degradation of FIZ1 (**9–12**). (B) Normalized eGFP/mCherry fluorescence ratio for ZF8-EGFP constructs stably expressed in Jurkat cells after 18 h treatment with a concentration curve of **5**, **8**, or **9** (1 pM–10 μM). (C) An overlay of the X-ray crystal structure of the CRBN (PDB ID: 6H0F) (carbon = green) ternary complex with pomalidomide and IKZF1 ZF2 (not shown) with an AlphaFold model of FIZ1 ZF8 (carbon = blue), docked to ^*t*^Bu-Thal (**5**) (carbon = yellow) or ‘compound 1′ (**9**) (carbon = lilac). The ^*t*^Bu-Thal (**5**) docked structure was relaxed using molecular dynamics. In the ‘compound 1′ (**9**) docked structure, the ‘repair’ function in FoldX to optimize the amino acid side chains. (D) An overlay of the X-ray crystal structure of the CRBN (PDB ID: 6H0F) (carbon = green) ternary complex with pomalidomide and IKZF1 ZF2 (not shown) with an AlphaFold model of FIZ1 ZF8 (carbon = blue), docked to ^*t*^Bu-Thal (**5**) (carbon = yellow) or UCLA-CFA154 (**8**) (carbon = dark blue). The ^*t*^Bu-Thal (**5**) docked structure was relaxed using molecular dynamics. In the UCLA-CFA154 (**8**) docked structure the ‘repair’ function in FoldX to optimize the amino acid side chains.

FIZ1 ZF11 also possesses relatively sterically unencumbered residues in the four interface amino acids. Position 471 (equivalent of IKZF3 position 147) is a conformationally restrained proline and 478 (equivalent of IKZF3 position 154) is glycine (Figure S9). Docking studies, conducted in a similar manner to those described above, predict how ^*t*^Bu-Thal (**5**) could be accommodated at the interface of CRBN and FIZ1 ZF11 (Figure S9), perhaps explaining the modest degradation seen in the flow cytometry assay (Figure 4F).

All other FIZ1 ZFs investigated here possess a set of larger amino acids at the 4 interface amino acids, for example Ala, His, and Pro at the equivalent of position 147, providing less flexibility to accommodate the ^*t*^Bu group. This is consistent with the lack of ^*t*^Bu-Thal (**5**)-induced degradation observed in the flow cytometry assay (Figure 4F).

During the course of this work four other examples of IMiD derivatives that induce degradation of FIZ1 were reported. Jochem et al. described “compound 1” (**9**, Figure 8) as a FIZ1 degrader, and reported that it also degrades IKZF1, IKZF2, and ZFP91 to a similar degree.^56^ Sylvain et al. recently detailed the development of NK7–902 (**10**, Figure 8), which is an IMiD-based degrader of the mitotic kinase NEK7. They show that this compound also degrades FIZ1, SALL3, and IKZF4.^25^ Xu et al. identified DKY709 (**11**, Figure 8) as a relatively weak and unselective degrader of FIZ1,^57^ and Steger et al. elucidated the structurally related SJ11322 (**12**, Figure 8) as a seemingly more potent FIZ1 degrader, which also leads to degradation of at least three other proteins to a similar degree.^20^ Our proteomics analysis quantified all the off-targets of previously reported FIZ1 degrader molecules in ESC and/or Jurkat cells, and none of these proteins showed a decrease in their expression after ^*t*^Bu-Thal (**5**) treatment, unlike FIZ1 levels (Figure S10). This further confirms the selectivity of ^*t*^Bu-Thal (**5**), which is different from other reported degrader molecules.

These compounds all possess substituents at the 4- (**9**) or 5-position of the thalidomide (**10**) or lenalidomide (**11–12**) ring. The existence of multiple compounds that induce degradation of FIZ1 to various degrees, and with varying selectivity, enables us to start understanding the structure–activity relationships of FIZ1 degradation. None of the reports investigated which of the FIZ1 ZFs are engaged by the molecular glue degraders. To investigate this, we synthesized “compound 1” (**9**) and its 5-position analog UCLA-CFA154 (**8**) (Figure 8A) and evaluated the ability of these compounds to induce degradation of FIZ1 ZF8 in the flow cytometry assay. Both **8** and **9** induced efficient degradation of this ZF8 construct, with DC_50_ values of 1.37 and 2.75 nM, respectively, which is similar to the DC_50_ value of ^*t*^Bu-Thal (**5**) which is 4.88 nM (Figure 8B). All compounds induced almost full degradation of the protein, with ^*t*^Bu-Thal (**5**) and **8** having *D*_max_ value of 98% and **9** having a *D*_max_ value of 95%. These data show that ^*t*^Bu-Thal (**5**) and **8** both induce degradation of FIZ1, at least in part, through effective engagement of ZF8. Modeling studies conducted on compound **9** (Figure 8C) and **8** (Figure 8D), using the same approach as outlined above, predict how the presence of G359 and G361 at CRBN-ZF8 interface enable accommodation of the cyclopropylamino groups of these compounds. Compound **9** is predicted to form a hydrogen bond with the backbone carbonyl group of CRBN E377, which is the residue that pomalidomide (**2**) interacts with when bound to CRBN and IKZF1 ZF2 (PDB ID: 6H0F). This observation suggests that IMiDs that form this interaction with CRBN might enable engagement with a range of ZFs, leading to the ability of **9** to induce degradation of IKZF1, IKZF2, and ZFP91, in addition to FIZ1. We have not further investigated the selectivity of compound **8**, but this hypothesis suggests that it would not induce degradation of IKZF1, IKZF2, or ZFP91. It is interesting, however, that the relatively small structural change between the 5-position ^*t*^Bu group (**5**) and the 4-cyclopropylamino group (**9**) has a significant effect on engagement with other ZF-containing proteins, and consequently compound selectivity. While only degradation of FIZ1 was observed in Jurkat or ESC cell lines after treatment with ^*t*^Bu-Thal (**5**, 10 μM, 16 h), treatment of Jurkat cells with **9** (5 μM, 13 h)^56^ resulted in degradation of IKZF1, IKZF3, and ZFP91, in addition to FIZ1. Although it is not known which ZFP91 ZF engages with **9**, we can assume that, like other IMiDs, it will bind to ZF2 of IKZF1 and 3, indicating that the 4-cyclopropylamino substituent is accommodated in the CRBN ternary complex with these ZFs.

In conclusion, we report the serendipitous discovery of ^*t*^Bu Thal (**5**) as an exquisitely selective molecular glue degrader of the C2H2 ZF-containing transcription factor FIZ1 in a range of cell lines. Analysis of the six CXXCG degron-containing FIZ1 ZFs revealed that ^*t*^Bu group (**5**) induces degradation of FIZ1 exclusively through engagement with ZF8. This finding was confirmed by overexpressing either WT or G364A V5-tagged FIZ1 in HEK293T cells, and demonstrating that ^*t*^Bu-Thal (**5**) induced degradation of the WT protein, but not the G364A mutant in which the ZF8 CXXCG degron is disrupted. Computational modeling studies suggest that the presence of the sterically unencumbered G359 and G361 residues at CRBN-ZF8 interface enable accommodation of the bulky ^*t*^Bu substituent of ^*t*^Bu-Thal (**5**). Comparison with other small IMiD derivatives that have been reported, albeit unselectively, to induce degradation of FIZ1 allows us to start understanding SAR in CRBN-IMiD-ZF ternary complexes. Our study suggests that the ZF residues that are proximal to CRBN in the ternary complex, play an important role in defining the IMiD binding site at the interface of CRBN and the ZF. This is in line with our previous work, which found that the residues at positions 147, 149, 150, and 154 of IKZF1/3 ZF2 most affected which IMiD analogs were accommodated by unnatural ZFs,^36^ and here we extend that concept to endogenous ZFs.

Studies in MOLM13 cells using ^*t*^Bu-Thal (**5**) showed no significant changes in the proteome beyond the notable reduction in FIZ1. This indicates that role of FIZ1 in hematologic malignancies needs further investigation, and ^*t*^Bu-Thal (**5**) could be a useful tool compound to further this research, and additional studies on the cellular functions of FIZ1. Analysis following ChIP-seq studies identified a motif matching the CRX binding site, consistent with reports that FIZ1 is involved in regulating CRX transcriptional activation.^33^ We also identified enriched motifs resembling binding sites of the transcription factors ZBTB32 and ARID3a, which have not previously been associated with FIZ1. IP-MS studies indicated that FIZ1 associates with C1QBP and YBX1. These proteins have previously been shown to interact with each other, and as C1QBP is involved in DNA damage repair,^55^ this result suggests that the function of FIZ1 in this process warrants further investigation. These data show that ^*t*^Bu-Thal (**5**) is a powerful molecular tool to selectively dissect the role of FIZ1 in complex biological settings.

## Supplementary Material





## Notes

Quantitative proteomics profiling; commercial FIZ1 antibodies exhibit non-specificity for endogenous protein; bar graphs; material and methods sections, the chemistry experimental section, and the computational methods section, ^1^H, ^13^C NMR spectra and HPLC traces of biologically tested compounds (PDF)
Jurkat dataset; hESC (H9) dataset: MM.1S dataset; MOLM13 dataset (XLSX)
