# Supplementary material for: 5‑ t Bu-Thalidomide Is a Selective Molecular Glue Degrader of the Transcription Factor FLT3 Interacting Zinc Finger 1 (FIZ1)
Source: J Am Chem Soc. 2026 Sep 2;148(36):39285–98. doi: 10.1021/jacs.6c14185 (PMC13587684; doi:10.1021/jacs.6c14185)
Supplement: Supplementary file 1 [file ja6c14185_si_001.pdf]

5-^{t}Bu-thalidomide is a potent and selective molecular glue degrader of the transcription factor FLT3 interacting zinc finger 1 (FIZ1)

Patrick J. Brennan,[1, 2‡] Ruiyang Guo,[3, 4‡] Alban Ordureau,[5] Sarah E. Singleton,[1] Yang Li,[1] Fatma Albayrak,[1,6] Connor H. Chang,[1] Lorena Yu Liao,[1] Charlotte M. Deane,[7] Kendall N. Houk,[1, 9, 10] Michael M. Hann,[8] Lewis L. Brayshaw,[8] Heeseon An,[3, 4] Stuart J. Conway[1, 2, 9, 10, 11]**

Supporting Information

[1]Department of Chemistry & Biochemistry, University of California, Los Angeles; Los Angeles, USA.
[2]Department of Chemistry, Chemistry Research Laboratory, University of Oxford; Oxford, UK.
[3]Chemical Biology Program, Sloan Kettering Institute, Memorial Sloan Kettering Cancer Center; New York, USA.
[4]Tri-Institutional PhD Program of Chemical Biology, Sloan Kettering Institute, Memorial Sloan Kettering Cancer Center, New York, NY, USA.
[5]Cell Biology Program, Memorial Sloan Kettering Cancer Center; New York, USA.
[6]Department of Chemistry, Gebze Technical University, Kocaeli, Türkiye (TR).
[7]Department of Statistics, University of Oxford; Oxford, UK.
[8]GSK, Medicines Research Centre; Stevenage, UK.
[9]California NanoSystems Institute, University of California Los Angeles, Los Angeles, USA.
[10]Molecular Biology Institute, University of California Los Angeles, Los Angeles, USA.
[11]Jonsson Comprehensive Cancer Center, University of California Los Angeles, Los Angeles, USA.

‡These authors contributed equally to this work

*To whom correspondence should be addressed: stuartconway@ucla.edu; anh@mskcc.org

Supplemental Figures

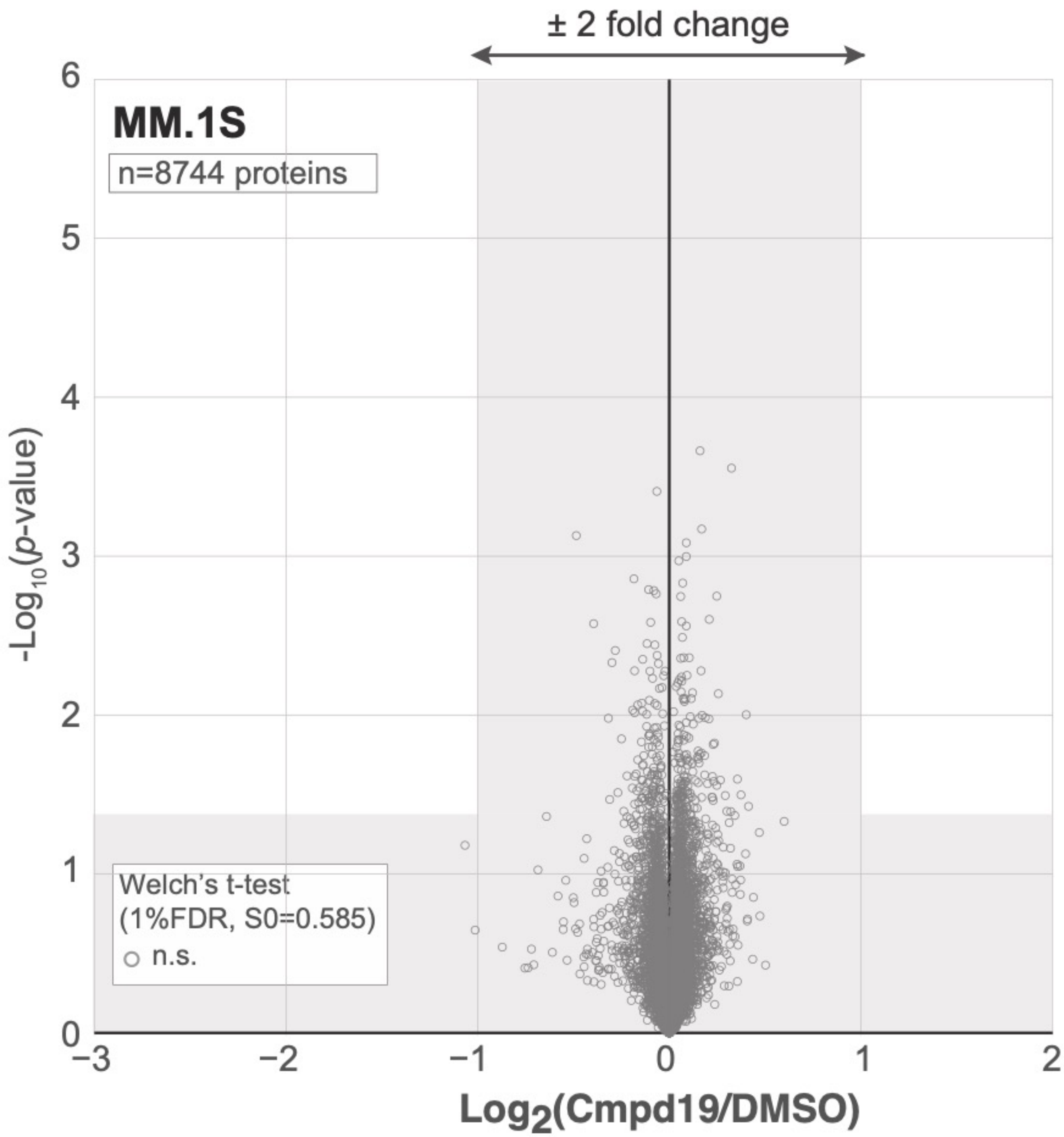

Figure S1. Quantitative proteomics profiling: MM.1S cells were treated for 16 h with either 5-^{t}Bu-Thal (**5**, 10 µM) or a DMSO control. Protein abundance was analyzed using multiplexed TMTpro quantification mass spectrometry. $\log_2$FC is shown on the x-axis, and $-\log_{10}$(*p*-value) is shown on the y-axis. Values shown are the mean of three biological replicates.

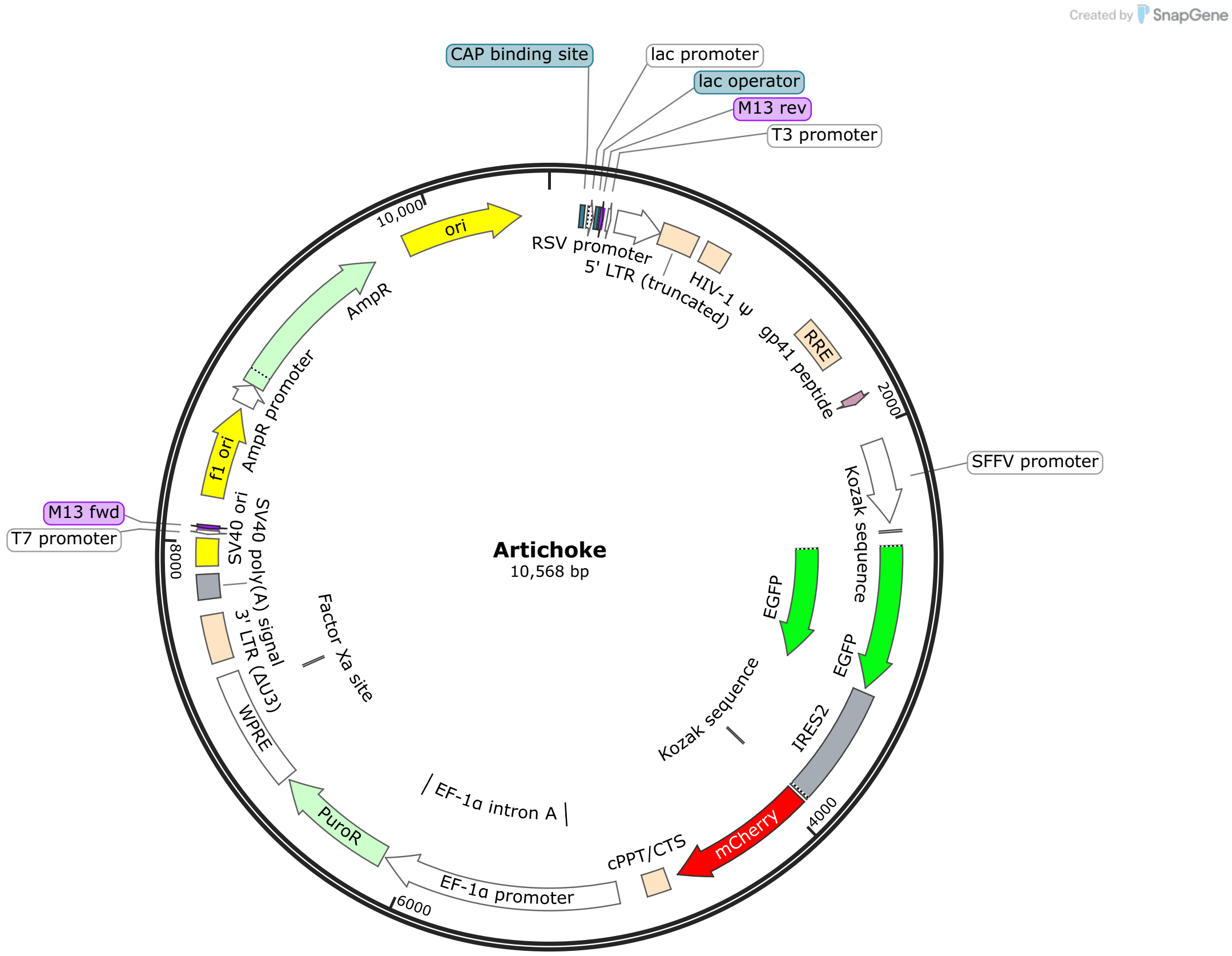

Figure S2. A map of the Artichoke plasmid (Addgene #73320).

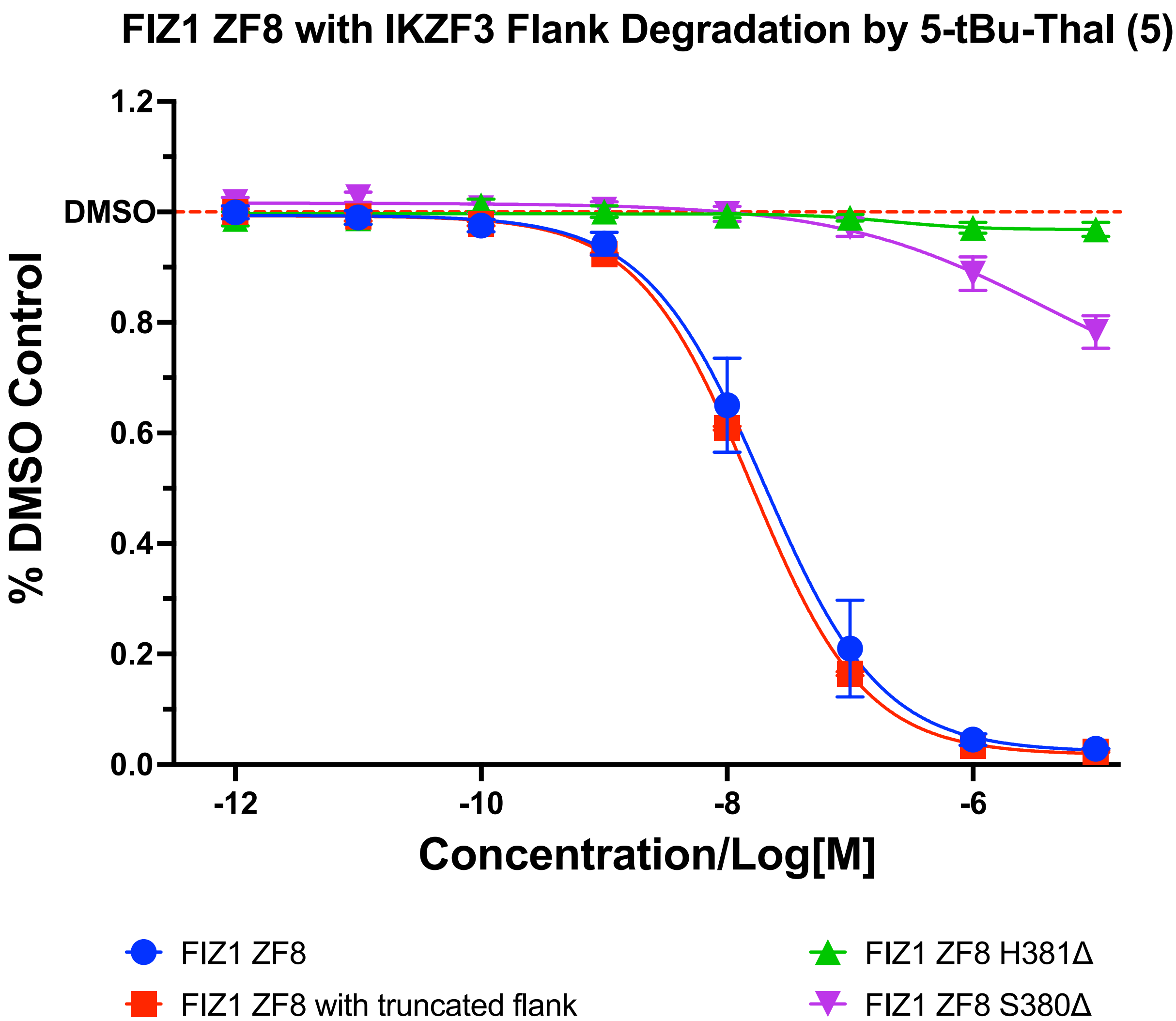

Figure S3. Normalized eGFP:mCherry fluorescence ratio for ZF-EGFP constructs stably expressed in Jurkat cells after 18 h treatment with a concentration curve of 5-^{t}Bu-Thal (**5**, 1 pm – 10 μM). DMSO 0.1% concentration in all conditions. The y-axis shows normalized EGFP:mCherry ratio compared to untreated (DMSO) control. Sequences are variants of FIZ1 ZF8 flanked by regions from IKZF3 as described in Figure 4. FIZ1 ZF8' features IKZF3 residues 169-189 as the C-terminal flanking region, while 'FIZ1 ZF8 with truncated flank' features IKZF3 residues 170-189 as the C-terminal flanking region.

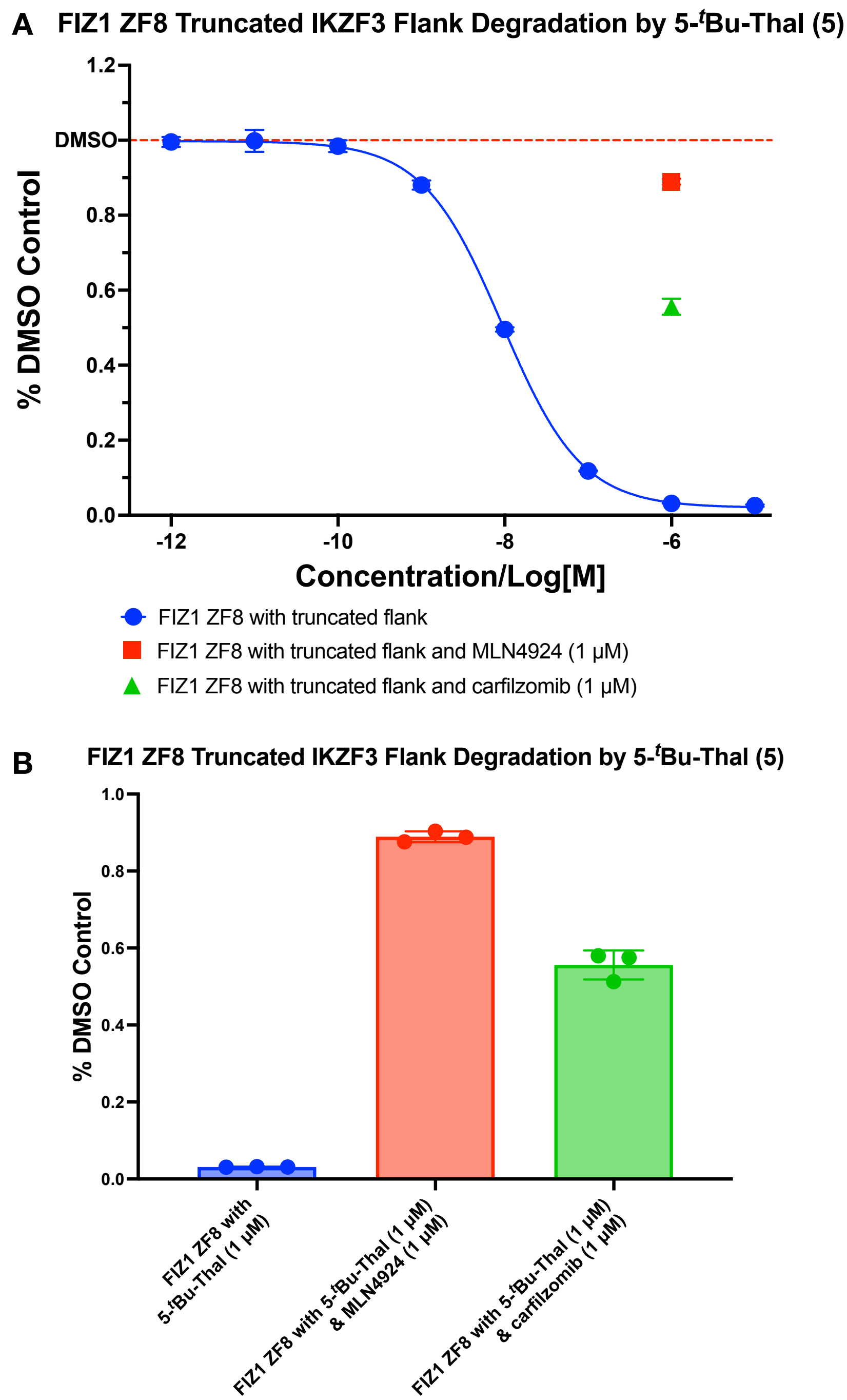

Figure S4. A. Normalized eGFP:mCherry fluorescence ratio for ZF8-EGFP construct stably expressed in Jurkat cells after 18 h treatment with a concentration curve of 5-^{t}Bu-Thal (**5**, 1 pM – 10 µM): either compound only or with MLN4924 (1 µM) or carfilzomib (1 µM). DMSO 0.2% concentration was included in all conditions. The y-axis shows normalized EGFP:mCherry ratio compared to untreated (DMSO) control. **B.** A bar graph showing the rescue of FIZ1 ZF8 degradation upon treatment with MLN4924 (1 µM) or carfilzomib (1 µM).

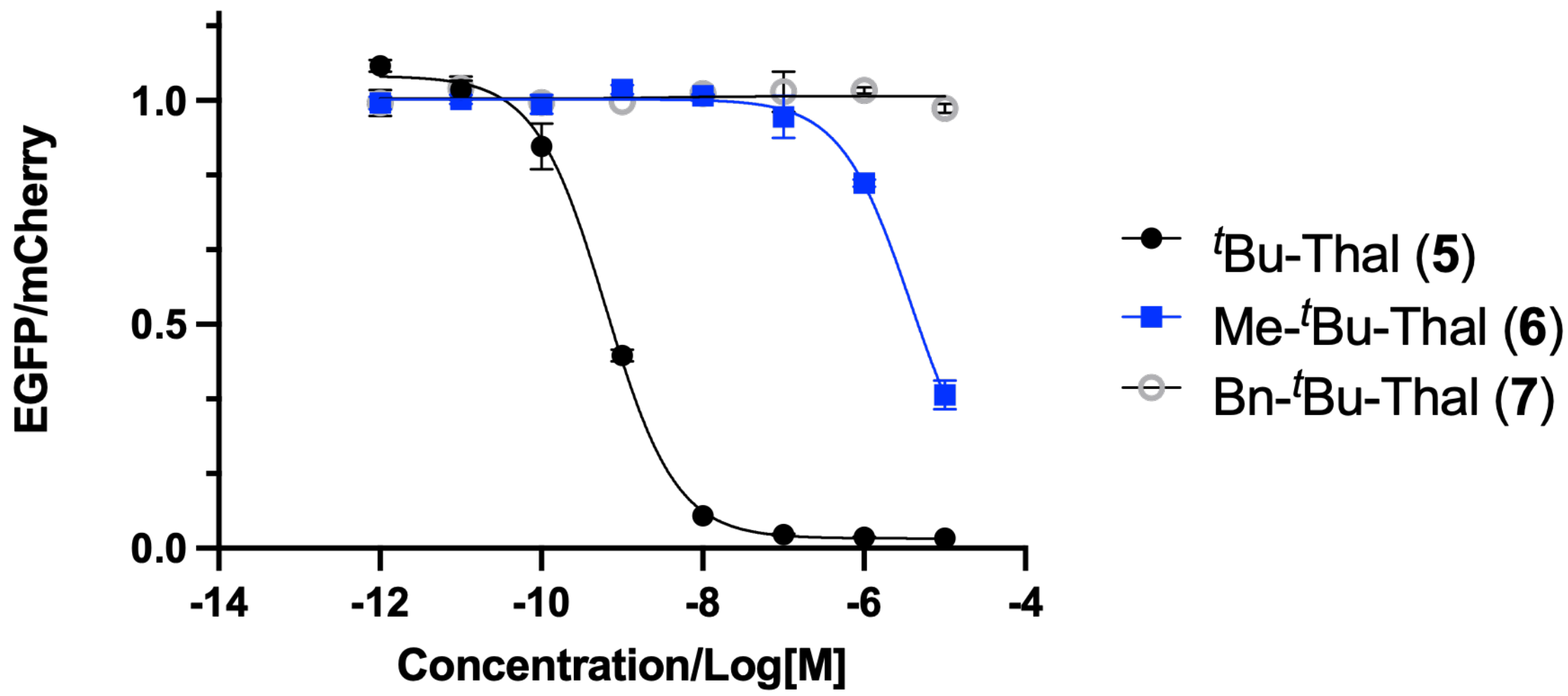

Figure S5. *N*-Methyl (**6**) and *N*-benzyl (**7**) derivatives of ^{t}Bu-Thal (**5**) induce less protein degradation than ^{t}Bu-Thal (**5**). Transduced Jurkat cells expressing the FIZ1 ZF8-EGFP fusion protein were incubated for 6 h with ^{t}Bu-Thal (**5**) (black), Me-^{t}Bu-Thal (**6**) (blue), or Bn-^{t}Bu-Thal (**7**) (grey) from at concentrations ranging from 10 μM to 1 pM. EGFP/mCherry levels were detected using flow cytometry to give a degradation curve.

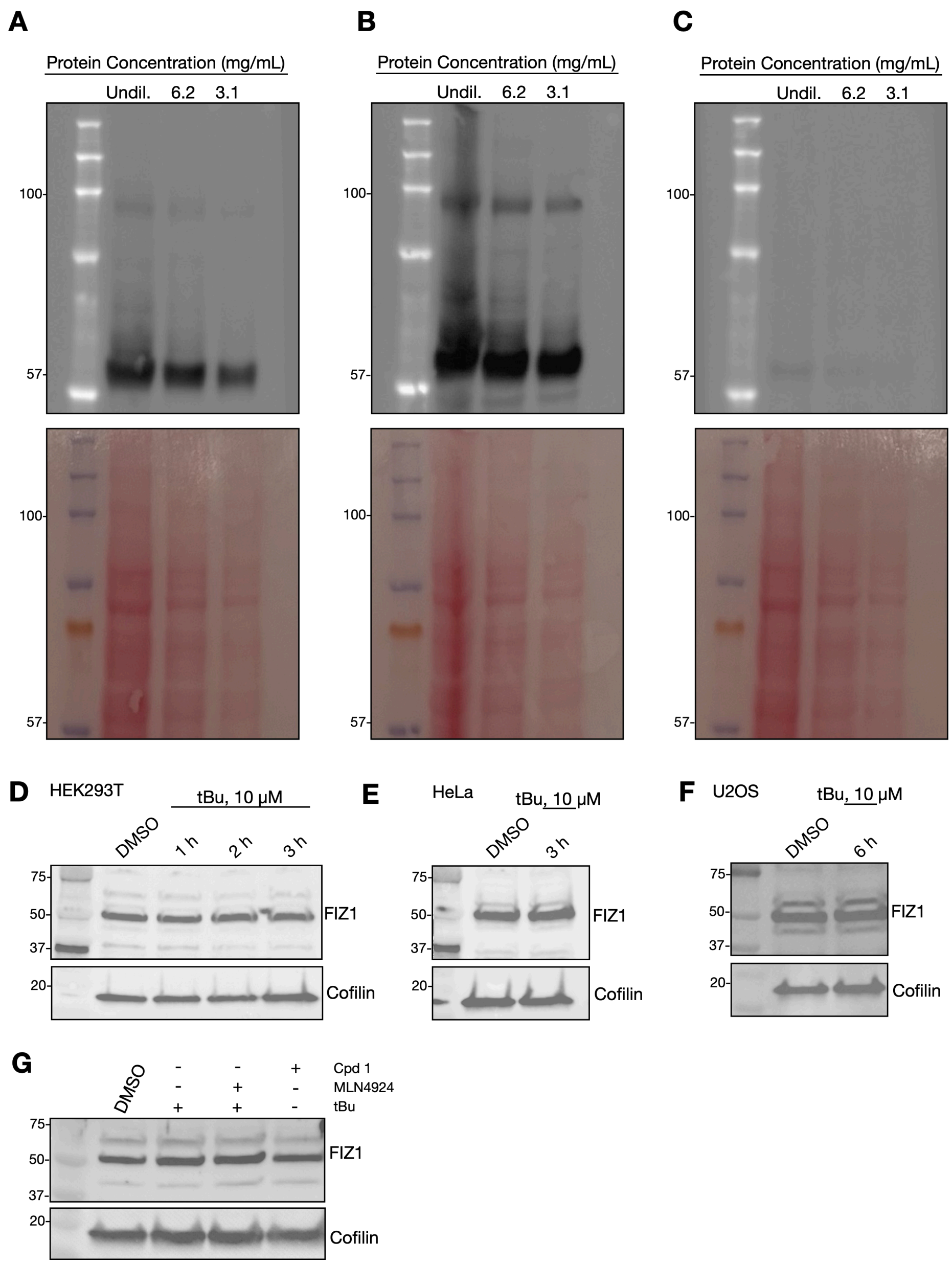

Figure S6. Commercial FIZ1 antibodies exhibit non-specificity for endogenous protein. Representative western blots of FIZ1 across multiple cell lines and treatment conditions. (**A**–**C**) Detection in FIZ1-overexpressing HEK293T cells using: (**A**) Cusabio (Cat# PA839415LA01HU, 1:2000), (**B**) Proteintech Domain 1 (15826-1-AP, 1:1000), and (**C**) Proteintech Domain 2 (15827-1-AP, 1:1000) antibodies. (**D**–**F**) Detection of endogenous FIZ1 using Thermo PA-98688 (1:1000) in: (**D**) wild-type (WT) HEK293T cells sampled at 1, 2, and 3 h, (**E**) WT HeLa cells treated for 3 h, and (**F**) WT U2OS cells treated with 10 µM tBu for 6 h. (**G**) Endogenous FIZ1 detection in WT HEK293T cells treated for 6 h using Thermo PA5-40308 (1.25 µg/mL). For panels **D**–**G**, cofilin served as the housekeeping loading control. Unless otherwise noted, 'Compound 1' (**9**) and ^{t}Bu-Thal (**5**) were used at 10 µM; where indicated, cells were pre-incubated with 1 µM neddylation inhibitor MLN4924 for 45 min.

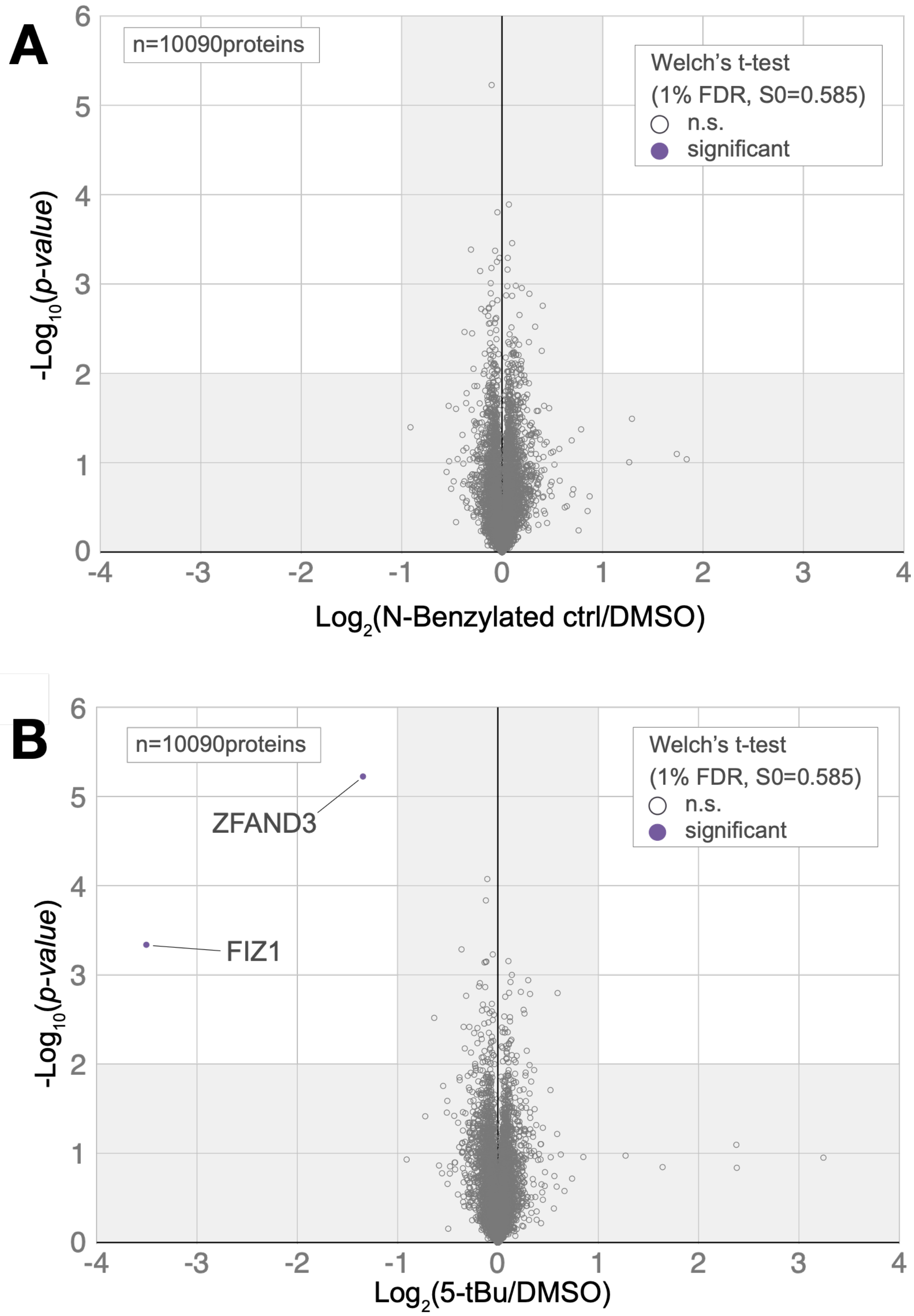

Figure S7. Quantitative proteomics profiling: TMT11-plex data of HCT116 cells treated for 6 h with DMSO, Bn-^{t}Bu-Thal (**7**, 5 μM), ^{t}Bu-Thal (**5**, 5 μM). Protein abundance was analyzed using multiplexed TMT quantification mass spectrometry. The x-axis shows (**A**) Log_2FC of the Bn-^{t}Bu-Thal (**7**) treated cells *versus* DMSO treated cells or (**B**) Log_2FC of ^{t}Bu-Thal (**5**) treated cells *versus* DMSO treated cells and the y-axis shows $-\log_{10}$(*p-value*). Values shown are the mean of at least three biological replicates.

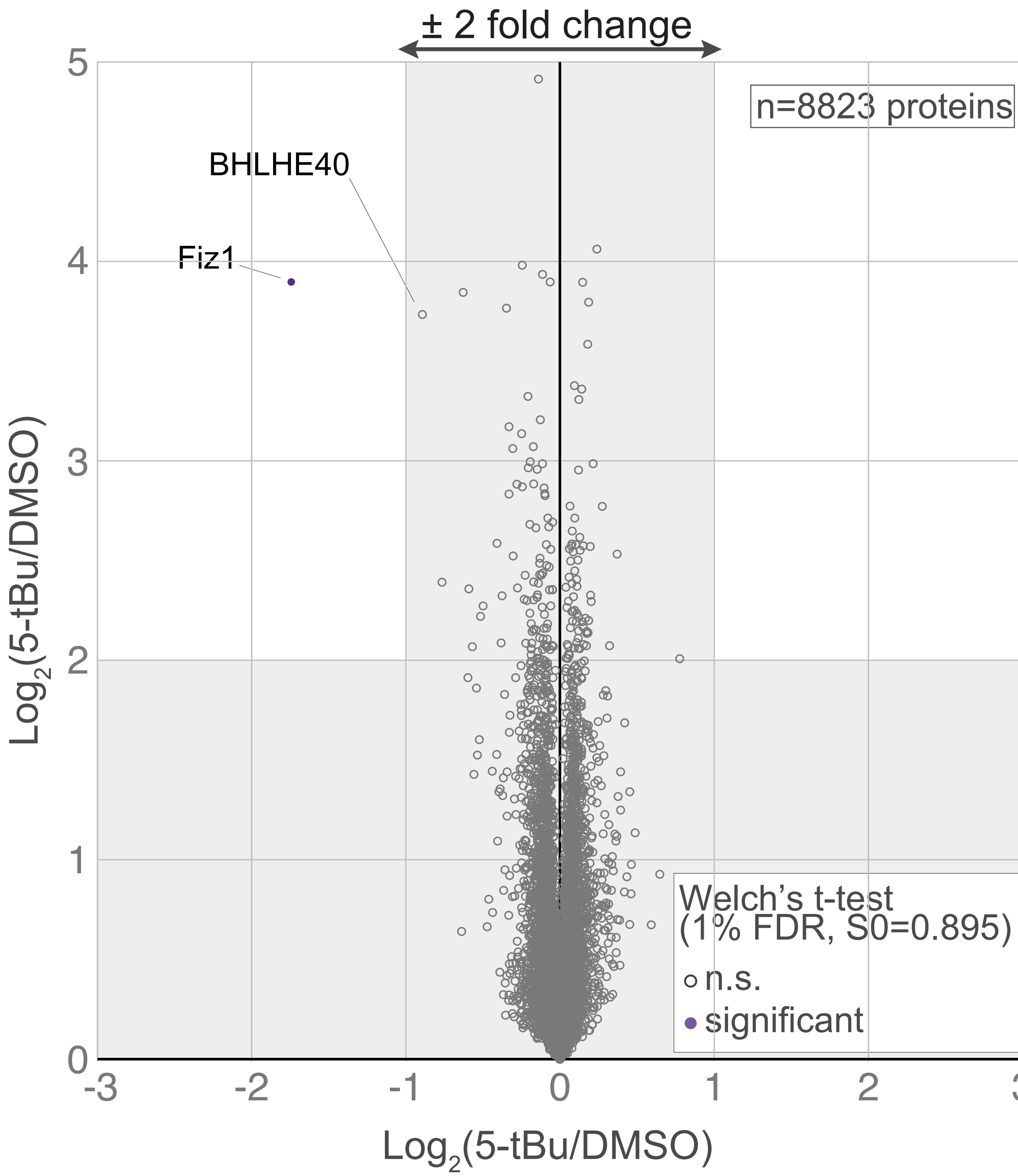

Figure S8. Quantitative proteomics profiling: MOLM13 cells were treated for 72 h with either 5-^{t}Bu-Thal (**5**, 10 µM) or a DMSO control. Protein abundance was analyzed using multiplexed TMTpro quantification mass spectrometry. $\log_2$FC is shown on the x-axis, and $-\log_{10}$(*p*-value) is shown on the y-axis. Values shown are the mean of three biological replicates.

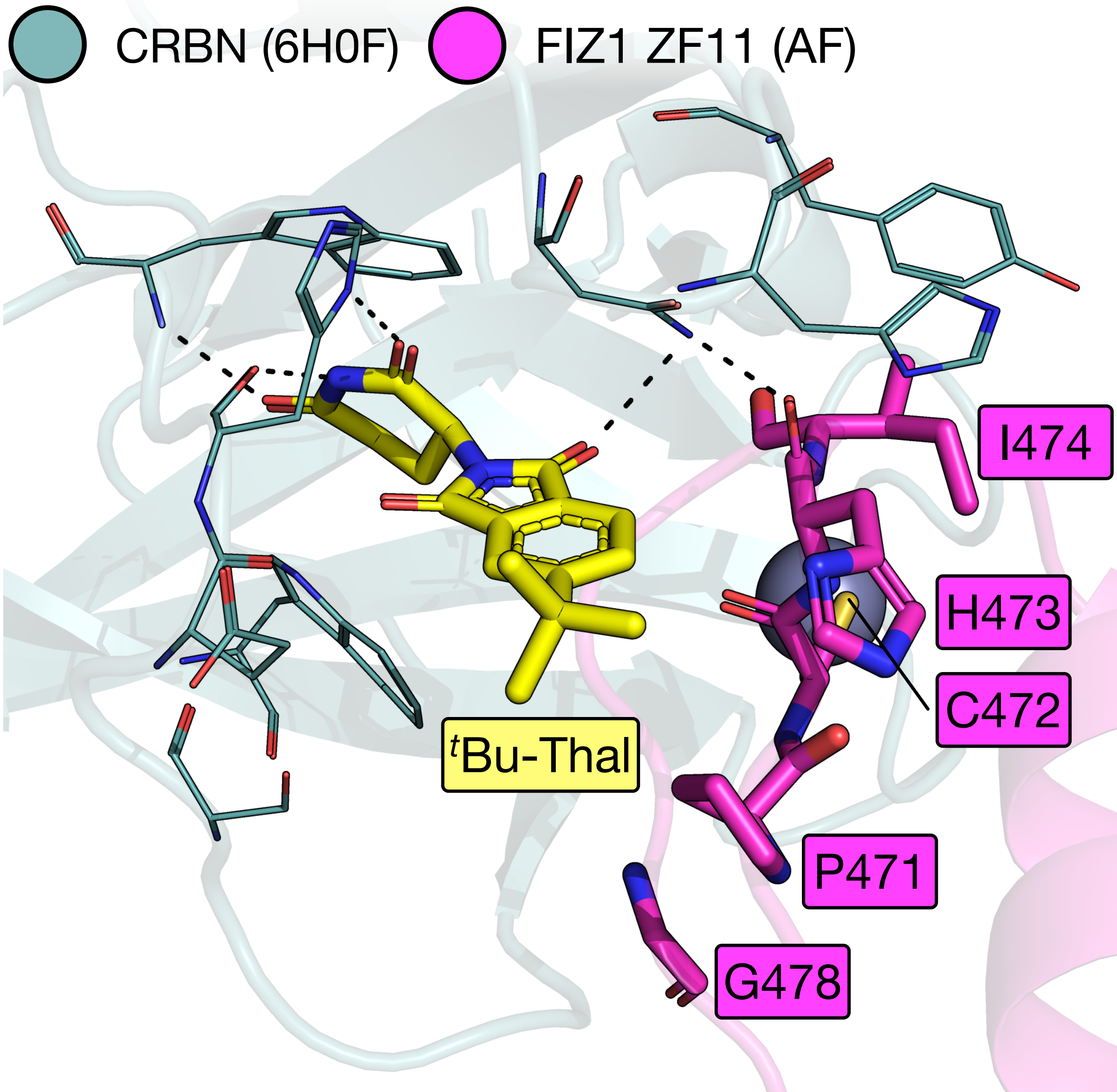

Figure S9. FIZ1 ZF11 (carbon = pink) from the AlphaFold predicted structure for FIZ1 was aligned with the IKZF1 ZF present in crystal structure 6H0F.[1] The IKZF1 ZF was then removed and the original pomalidomide ligand was replaced with 5-^{t}Bu-Thal (**5**, carbon = yellow); then the resulting CRBN-IMiD-ZF11 ternary complex was relaxed for 2 ns using molecular dynamics. CRBN (carbon = green; Zn^{2+} (dark grey).

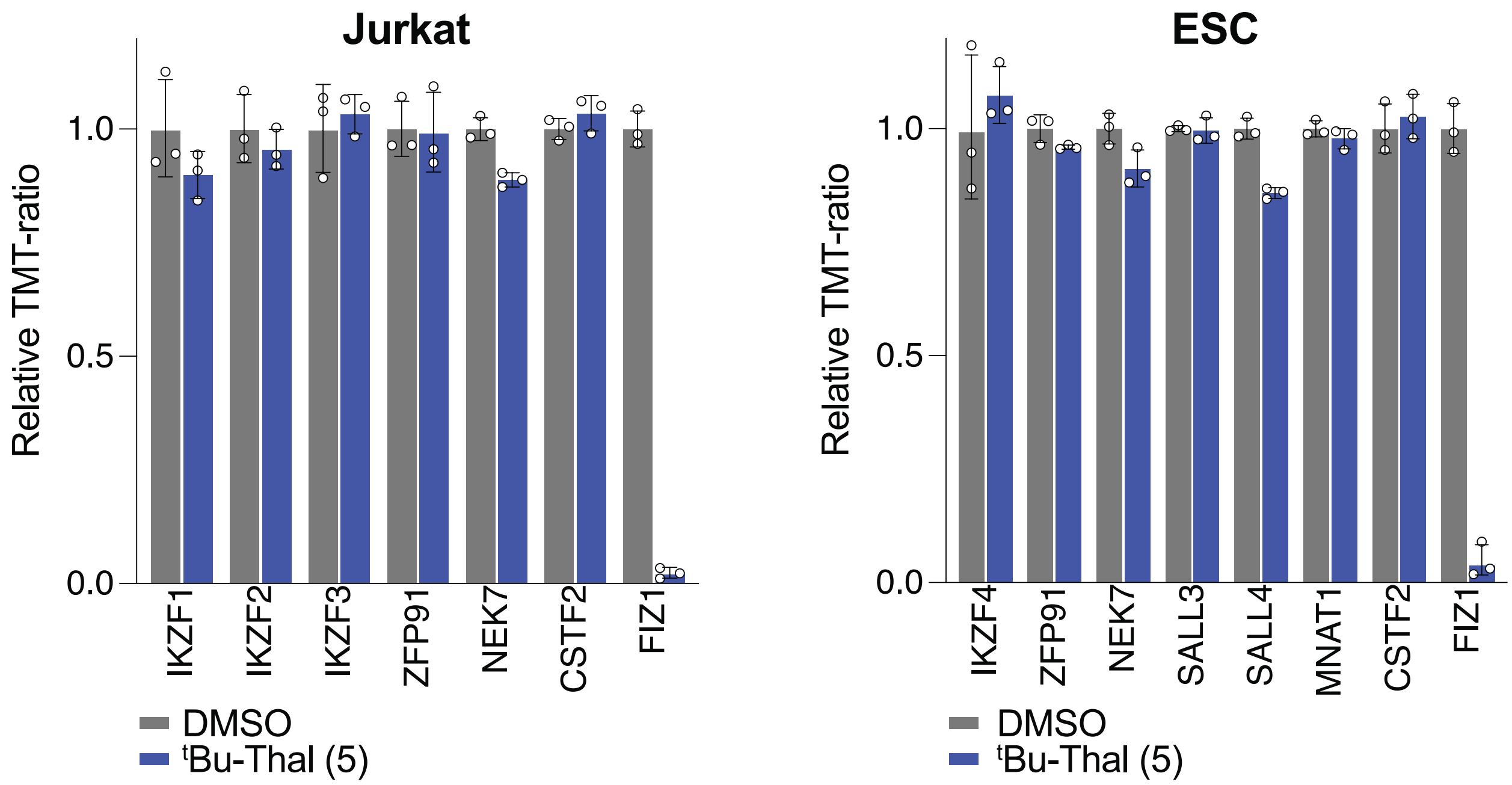

Figure S10. Bar graphs showing the relative protein expression quantified by TMTpro proteomics in Jurkat and ESC cells, after treatment with *ᵗ*Bu-Thal (**5**) for 16 h. *ᵗ*Bu-Thal (**5**) does not degrade other proteins targeted by previously reported FIZ1 degraders.

Materials and Methods

Chemistry Experimental Section

Reagents and solvents used were of commercially available reagent grade quality from Alfa Aesar, Fluorochem, Merck (formally known as Sigma-Aldrich), or Tokyo Chemical Industry, and were used without further purification unless stated. Anhydrous solvents were obtained from an MBRAUN Solvent Purification Systems 5 and stored under an argon atmosphere over 3 Å molecular sieves. Concentration *in vacuo* was performed at 40 °C for organic solvents using a Buchi™ rotary evaporator. Brine refers to a saturated aqueous solution of sodium chloride. Petroleum ether refers to the fraction of light petroleum ether boiling in the range 40-60 °C. Celite® refers to Celite® 545 filter aid, treated with sodium carbonate, flux-calcined (Merck).

^{1}H NMR spectra were measured on a Bruker AV400 (400 MHz), a Bruker AVII 500 (500 MHz) or a Bruker AV600 (600 MHz) spectrometer in the stated solvents as a reference for the internal deuterium lock. The chemical shift data for each signal are given as δ in units of parts per million (ppm) relative to tetramethylsilane (TMS) where δ(TMS) = 0.00. The spectra are calibrated using the solvent peak with the data provided by Fulmer *et al.*.[2] The multiplicity of each signal is indicated by: s (singlet); bs (broad singlet); d (doublet); t (triplet); q (quartet); p (pentet); sept (septet); m (multiplet) or combinations thereof. The number of protons (n) for a given resonance signal is indicated by nH. Where appropriate, coupling constants (*J*) are quoted in Hz, recorded to the nearest 0.1 Hz, and were determined by analysis using Bruker TopSpin v3.2 software or MestreNova software. The mean value of identical coupling constants is reported. Spectra were assigned using COSY, HSQC and HMBC experiments as necessary. Spectra acquired at high temperature (353 K or 363 K) are indicated. ^{13}C NMR spectra were measured on a Bruker AV400 (101 MHz), a Bruker AVII 500 (126 MHz) or a Bruker AV600 (151 MHz) spectrometer in the stated solvents as a reference for the internal deuterium lock using the standard ^{13}C experiment. The chemical shift data for each signal are given as δ in units of parts per million (ppm) relative to tetramethylsilane (TMS) where δ(TMS) = 0.00.

The spectra are calibrated using the solvent peak with the data provided by Fulmer *et al.*.[2] Signals are quoted to one decimal place unless peaks are indistinguishable, in which case two decimal places are used. Where appropriate, coupling constants (*J*) are quoted in Hz, recorded to the nearest

0.1 Hz, and were determined by analysis using Bruker TopSpin v3.2 software or MestreNova software. Spectra were assigned using HSQC and HMBC experiments as necessary.

Spectra acquired at high temperature (353 K or 363 K) are indicated. ^{19}F NMR spectra were measured on a Bruker AVIII HD 400 (376 MHz) spectrometer in the solvent stated, and are ^{13}C decoupled. Chemical shifts are given as δ in units of parts per million (ppm), to the nearest 0.1 ppm. The multiplicity of each signal is a singlet unless stated otherwise.

Mass spectra were acquired on either an Agilent 6120 (low resolution) or Bruker microToF spectrometer (high resolution) using electrospray ionization (ESI) from solutions of either methanol or water. *m/z* values are reported in Daltons and are followed by their percentage abundance in parentheses.

Melting points were determined using a Griffin capillary tube melting point apparatus (Registered Design No. 889339) and are uncorrected. The solvent(s) from which the sample was crystallized is given in parentheses. Dec. indicates that the sample decomposed at the stated temperature.

Infrared spectra were obtained from thin films, using a diamond ATR module on a Bruker Tensor 27 spectrometer. Absorption maxima are reported in wavenumbers (cm^{-1}) and reported as s (strong), m (medium), w (weak) or br (broad).

Analytical high performance liquid chromatography (HPLC)

Method 1: Analytical HPLC was performed on a PerkinElmer 10 Flexar system with a binary LC Pump and UV/vis LC detector set at 254 nm or Agilent 1260 Infinity II® system equipped with a Poroshell 120 EC-C18 column [4 µm, 4.6 × 100 mm] with a diode array UV/vis detector. To determine compound purity, a Dionex Acclaim® 120 C18 [5 µm, 12 Å, 150 mm × 4.6 mm] reverse phase column was used with a constant flow rate of 1.5 mL min^{-1} and gradient method of 10 min from 95:5 H_2O:acetonitrile (0.1% TFA) to 5:95 H_2O:acetonitrile (0.1% TFA) with a 5 min hold. Samples injected were prepared by dissolving in methanol, water, or acetonitrile, and filtered. All samples are run with 0.1% trifluoroacetic acid or 0.1% formic acid added unless indicated.

Method 2: Analytical HPLC was performed on a Shimadzu system with Binary LC-40D Pump and UV/vis SPD-M40 Detector using the following: a Shim-pack Velox C18 column [5 µm, 100

mm × 4.6 mm] reverse phase column was used with a constant flow rate of 1.0 mL min-1 and gradient method started with H_2O:MeCN (95:5) for 1 min, followed by a 10 min linear gradient to H_2O:MeCN (5:95). The composition was then held for 5 min and returned to H_2O:MeCN (95:5) for 3 min to re-equilibrate the column (total 19 min). Samples injected were prepared by dissolving in MeOH, MeCN or H_2O, and filtered. All samples are run with 0.1% formic acid added unless indicated. Sample purity was measured and reported using the UV absorbance at 254 nm, 220 nm and 280 nm.

All biologically assessed compounds had a purity of ≥95% determined using analytical HPLC.

Normal Phase silica gel analytical Thin Layer Chromatography (NP TLC) to monitor reaction progress was carried out on normal phase Merck silica gel 60 F254 aluminium-supported thin layer chromatography sheets. Visualization was carried out using absorption of UV light (λ_{max} = 254 nm and 365 nm) or thermal development after staining in an ethanolic solution of ninhydrin or an aqueous solution of potassium permanganate.

Normal phase silica gel flash column chromatography was carried out manually on Merck Geduran® silica gel 60 (40–63 µm), eluting with solvents as supplied, under a positive pressure of compressed, gaseous, nitrogen.

Compound Synthesis

5-(*tert*-Butyl)-2-(2,6-dioxopiperidin-3-yl)isoindoline-1,3-dione (5-^{t}Bu-Thal, 5)

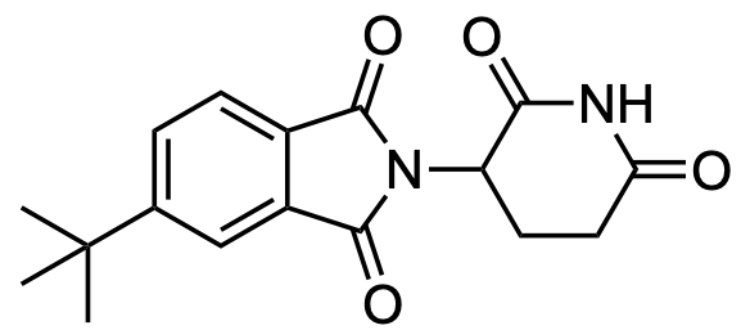

4-*tert*-Butylphthalic anhydride (200 mg, 0.979 mmol, 1.0 eq.), 3-aminopiperidine-2,6-dione hydrochloride (161 mg, 0.978 mmol, 1.0 eq) and NaOAc (121 mg, 1.47 mmol, 1.5 eq.) were dissolved in AcOH (5 mL) and heated at 120 °C under reflux for 21 h. The AcOH was removed *in vacuo* and the residue purified using flash column chromatography eluting with MeOH/CH_2Cl_2 (10/90) to afford 5-(*tert*-butyl)-2-(2,6-dioxopiperidin-3-yl)isoindoline-1,3-dione (**5**) as a colorless solid (139 mg, 45%): R_f 0.69 (10/90 MeOH/CH_2Cl_2); m.p. 145–155 °C (from $CHCl_3$); ^{1}H NMR (400 MHz, D_6-DMSO) δ 11.12 (1H, s), 7.96 – 7.82 (3H, m), 5.14 (1H, dd, *J* 12.9, 5.4 Hz), 2.96 – 2.82 (1H, m), 2.65 – 2.52 (2H, m), 2.11 – 1.99 (1H, m), 1.36 (9H, s); LRMS (ESI$^-$) *m/z* 313 ([M−H]$^-$, 100%); HPLC (*Method 1*) retention time = 9.7 min, purity: 220 nm, = 99.8%; 254 nm, >99.9%. These data are in good agreement with the literature values.[3]

5-(*tert*-Butyl)-2-(1-methyl-2,6-dioxopiperidin-3-yl)isoindoline-1,3-dione (6)

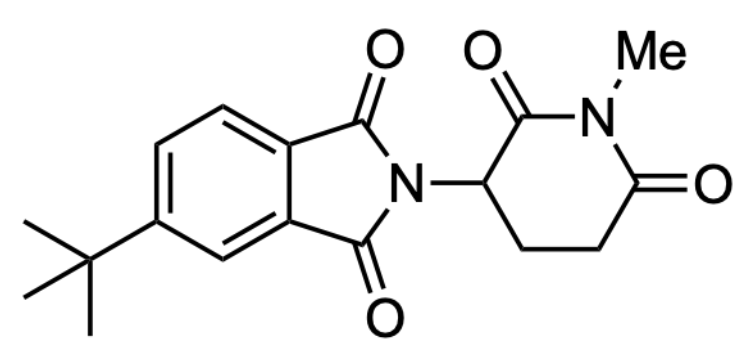

The mixture of 5-(*tert*-butyl)-2-(2,6-dioxopiperidin-3-yl)isoindoline-1,3-dione (281 mg, 0.89 mmol, 1 equiv) and K_2CO_3 (246 mg, 1.78 mmol, 2 equiv) in anhydrous DMF (2.5 mL) was added MeI (140 mg, 0.06 mL, 0.98 mmol, 1.1 equiv), stirred at 60 °C overnight. The reaction mixture was cooled to rt, quenched with H_2O (10 mL), and extracted with EtOAc (3 × 20 mL). The combined organic components were washed with brine (2 × 20 mL), dried over $MgSO_4$, filtered, and concentrated *in vacuo*. The crude residue was purified using silica gel flash column chromatography eluting with (0 – 1% MeOH/CH_2Cl_2) to give 5-(*tert*-butyl)-2-(1-methyl-2,6-dioxopiperidin-3-yl)isoindoline-1,3-dione (**6**) as a colorless solid (160 mg, 55%): R_f 0.30 (100%

CH_2Cl_2); m.p. 140 − 142 °C (from MeOH); $\overline{\nu}_{max}$ (neat)/cm^{-1} 3100.0, 3058.5, 2962.1, 2921.6, 1775.1, 1712.5, 1677.8, 1380.8, 1116.6; ^{1}H NMR (400 MHz, MeOD) δ_H 7.97 – 7.88 (m, 2H), 7.83 – 7.80 (m, 1H), 5.16 (dd, J = 13.0, 5.4 Hz, 1H), 3.18 – 3.12 (m, 3H), 2.99 – 2.84 (m, 2H), 2.77 – 2.66 (m, 1H), 2.17 – 2.06 (m, 1H), 1.41 (s, 9H) ppm; ^{13}C NMR (101 MHz, MeOD) δ_C 173.6, 171.2, 169.2, 168.8, 160.5, 133.3, 132.9, 130.5, 124.4, 121.5, 51.2, 36.7, 32.5, 31.4, 27.3, 22.9 ppm; HRMS (DART-Orbitrap) *m/z* [M + H]$^+$ Calcd for $C_{18}H_{20}N_2O_4$ 329.14958; Found 329.14940; LRMS (ESI$^+$) *m/z* 329.25 ([M + H]$^+$, 100%); HPLC (*Method 2*), retention time = 8.4 min, purity: 220 nm, 97.8%; 254 nm, 97.7%; 280 nm, 99.3%

2-(1-Benzyl-2,6-dioxopiperidin-3-yl)-5-(*tert*-butyl)isoindoline-1,3-dione (7)

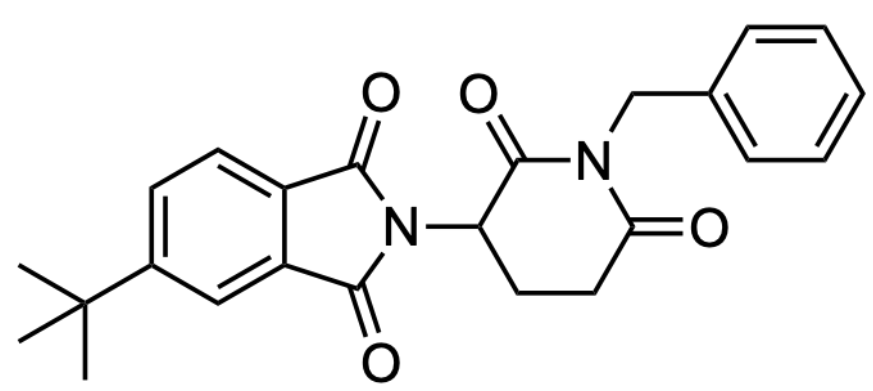

The mixture of 5-(*tert*-butyl)-2-(2,6-dioxopiperidin-3-yl)isoindoline-1,3-dione (260 mg, 0.83 mmol, 1 equiv) and K_2CO_3 (229 mg, 1.66 mmol, 2 equiv) in anhydrous DMF (3 mL) was added benzyl bromide (141 mg, 0.1 mL, 0.83 mmol, 1 equiv), stirred at 60 °C overnight. After this time, the reaction mixture was cooled to rt, quenched with H_2O (10 mL) and extracted with EtOAc (3 × 20 mL). The combined organic components were washed with brine (2 × 20 mL), dried over $MgSO_4$, filtered, and concentrated *in vacuo*. The crude residue was purified using silica gel flash column chromatography eluting with (0 – 30% EtOAc/*n*-Hexane). The resulting colorless oil was triturated with diethyl ether and isolated by filtration to give 2-(1-benzyl-2,6-dioxopiperidin-3-yl)-5-(*tert*-butyl)isoindoline-1,3-dione (**7**) as a pink solid (302 mg, 90%): R_f 0.54 (30% EtOAc/*n*-hexane); m.p. 141−143 °C (from MeOH); $\overline{\nu}_{max}$ (neat)/cm^{-1} 3065.3, 3030.3, 2955.8, 2906.4, 2866.3, 1776.1, 1710.8, 1679.2, 1621.4, 1371.7, 1345.6, 1159.2; ^{1}H NMR (400 MHz, DMSO) δ_H 7.93 – 7.91 (m, 2H), 7.87 – 7.85 (m, 1H), 7.37 – 7.18 (m, 5H), 5.36 (dd, J = 13.1, 5.4 Hz, 1H), 4.94 – 4.78 (m, 2H), 3.13 – 3.04 (m, 1H), 2.84 – 2.78 (m, 1H), 2.68 – 2.56 (m, 1H), 2.15 – 2.08 (m, 1H), 1.35 (s, 9H) ppm; ^{13}C NMR (101 MHz, DMSO) δ_C 171.6, 169.7, 167.4, 167.0, 158.7, 137.0, 131.7, 131.5, 128.7, 128.2, 127.1, 126.9, 123.4, 120.4, 49.6, 42.7, 35.5, 31.1, 30.7, 21.2 ppm; HRMS (DART-Orbitrap) *m/z* [M + H]$^+$ Calcd for $C_{24}H_{24}N_2O_4$ 405.18088; Found 405.17935;

LRMS (ESI^+) *m/z* 405.25 ($[M + H]^+$, 100%); HPLC (*Method 2*), retention time = 9.9 min, purity: 220 nm, 99.9%; 254 nm, 98.2%; 280 nm, 99.7%.

5-(Cyclopropylamino)-2-(2,6-dioxopiperidin-3-yl)isoindoline-1,3-dione (8)

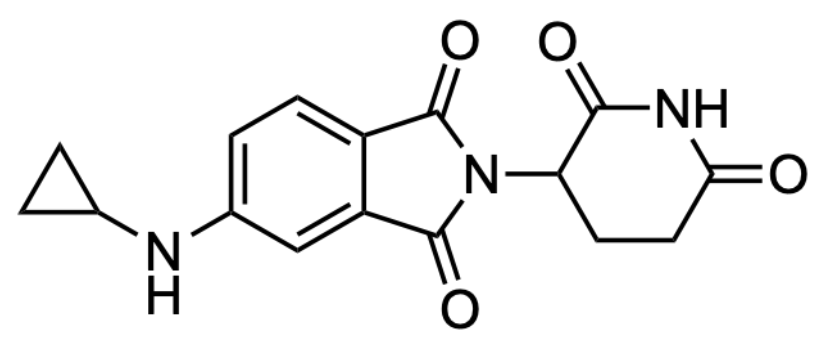

DIPEA (351 mg, 0.48 mL, 1.5 eq.) was added to a solution of 2-(2,6-dioxopiperidin-3-yl)-5-fluoroisoindoline-1,3-dione (500 mg, 1.81 mmol, 1.0 eq.) and cyclopropylamine (125 mg, 0.15 mL, 1.2 equiv) in anhydrous DMSO (2.5 mL) and the mixture was heated to 85 °C in a sealed tube overnight. After cooling to rt, the mixture was diluted with EtOAc (20 mL) and washed with brine (10 mL). The aqueous layer was extracted with EtOAc (2 × 15 mL), and the combined organic layers were dried over anhydrous $Na_2SO_{4(s)}$, filtered, and concentrated *in vacuo*. The crude material was purified using silica gel flash column chromatography eluting with $MeOH/CH_2Cl_2$ (0/100-1/99) to give *5-(cyclopropylamino)-2-(2,6-dioxopiperidin-3-yl)isoindoline-1,3-dione* (**8**) as a yellow solid (227 mg, 40%). R_f 0.33 (1% $MeOH/CH_2Cl_2$); m.p. 232−234 °C (from CH_2Cl_2); $\bar{\nu}_{max}$ (neat)/cm^{-1} 1533.8, 1613.0, 1700.6, 1763.0, 3006.1, 3095.5, 3202.7, 3328.5, 3377.0; ^1H NMR (400 MHz, MeOD) δ_H 7.58 (d, J = 8.3 Hz, 1H), 7.15 (d, J = 2.1 Hz, 1H), 7.00 (dd, J = 8.3, 2.1 Hz, 1H), 5.05 (dd, J = 12.5, 5.4 Hz, 1H), 2.91 – 2.80 (m, 1H), 2.78 – 2.64 (m, 2H), 2.50 (tt, J = 6.8, 3.7 Hz, 1H), 2.16 – 2.03 (m, 1H), 0.86 – 0.81 (m, 2H), 0.55 – 0.50 (m, 2H) ppm; ^{13}C NMR (126 MHz, MeOD) δ_C 174.7, 171.7, 169.6, 169.4, 156.9, 135.7, 126.0, 119.1, 117.4, 107.4, 50.3, 32.2, 25.4, 23.8, 7.7; LRMS (ESI^+) *m/z* 314.15 ($[M + H]^+$, 100%); HRMS (ESI^+) *m/z* $[M + Na]^+$ Calcd for $C_{16}H_{15}N_3O_4Na$ 336.0960; Found 336.0960; HPLC (*Method 2*) retention time = 6.3 min, purity: 220 nm, >99.9%; 254 nm, >99.9%; 280 nm, >99.9%.

4-(Cyclopropylamino)-2-(2,6-dioxopiperidin-3-yl)isoindoline-1,3-dione (9)

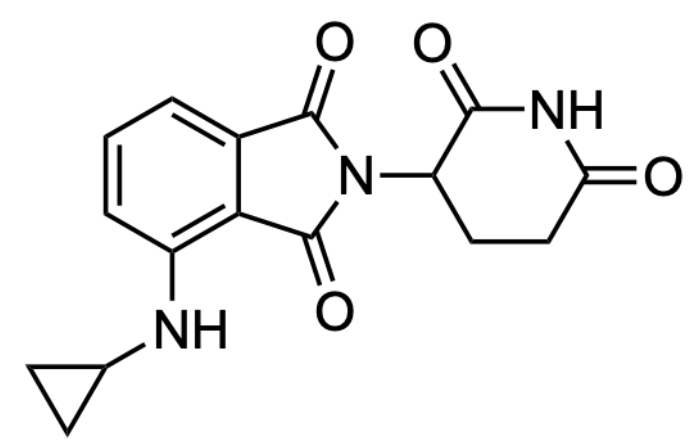

DIPEA (56.2 mg, 0.08 mL, 1.2 eq.) was added to a solution of 2-(2,6-dioxopiperidin-3-yl)-4-fluoroisoindoline-1,3-dione (100 mg, 0.362 mmol, 1.0 eq.) and cyclopropylamine (31 mg, 0.04 mL, 1.5 eq.) in anhydrous DMSO (1 mL) and the mixture was heated to 85 °C in a sealed tube overnight. After cooling to rt, the mixture was diluted with EtOAc (20 mL) and washed with brine (10 mL). The aqueous layer was extracted with EtOAc (2 × 15 mL) and the combined organic layers were dried over anhydrous Na_2SO_4(s), filtered, and concentrated *in vacuo*. The crude material was purified using silica gel flash column chromatography eluting with MeOH/CH_2Cl_2 (1/99) to give 4-(cyclopropylamino)-2-(2,6-dioxopiperidin-3-yl)isoindoline-1,3-dione (**9**) as a yellow solid (34.1 mg, 30%): R_f 0.34 (10/90 MeOH/CH_2Cl_2); m.p. 228−230 °C (from CH_2Cl_2); $\bar{\nu}_{max}$ (neat)/cm^{-1} 1617.7, 1692.2, 1735.1, 1757.4, 2888.7, 2922.2, 2957.6, 3272.6, 3340.6; ^{1}H NMR (500 MHz, MeOD) δ_H 7.63 (1H, d, *J* 8.6 Hz), 7.39 (1H, d, *J* 8.5 Hz), 7.12 (1H, d, *J* 2.1 Hz), 5.04 (1H, dd, *J* 12.4, 5.4 Hz), 2.89 – 2.81 (1H, m), 2.77 – 2.66 (2H, m), 2.50 (1H, tt, *J* 7.0, 3.6 Hz), 2.13 – 2.07 (1H, m), 0.90 – 0.85 (2H, m), 0.62 – 0.58 (2H, m) ppm; ^{13}C NMR (126 MHz, MeOD) δ_C 174.6, 171.6, 170.5, 169.3, 148.6, 136.9, 133.7, 119.3, 111.5, 107.4, 50.1, 32.1, 24.9, 23.7, 8.0 ppm; LRMS (ESI$^+$) *m/z* 314.20 ([M + H]$^+$, 100%); HPLC (*Method 2*) retention time = 6.9 min, purity: 220 nm, >98.6%; 254 nm, >98.6%; 280 nm, >99.9%. These data are in good agreement with the literature values.[4]

Biological Methods

Cell Culture

Jurkat cells were cultured in a media of RPMI (Gibco), 10% heat-inactivated FBS (Gibco), 1% Glutamax (Gibco), 1% MEM non-essential amino acids (Gibco), 1% sodium pyruvate (Gibco) and 1% PenStrep (Sigma). Lenti-X 293-T cells (Takara) were cultured in a media of DMEM high glucose (Gibco), 10% Tet-free FBS (Takara), 1% sodium pyruvate and 1% PenStrep. Cells were incubated at 37 °C and 5% CO_2.

HEK293T and HCT116 cell lines were cultured in Dulbecco's modified Eagle's medium (DMEM) high glucose and MOLM13 cells were grown in Roswell Park Memorial Institute (RPMI) 1640 medium in a 5% CO_2 incubator at 37 °C. DMEM and RPMI 1640 media were supplemented with 10% fetal bovine serum (Corning).

Cloning

The degradation reporter plasmid Artichoke (Addgene plasmid #73320) was used for ZF degradation assays. Gene fragments encoding FIZ1 ZF motifs as 60 or 61 residue hybrid tags with flanking IKZF3 regions were ordered from IDT and cloned into the empty Artichoke vector using the Golden Gate Assembly Kit BsmBI-v2 (New England Biolabs). PCR products were transformed into One Shot™ Stbl3™ Chemically Competent *E. coli* (ThermoFisher) and plasmids were purified using ZR Plasmid Miniprep kits (Zymo Research).

A synthetic DNA gene block (Integrated DNA Technologies) was used to generate a full-length pDONR223-FIZ1 plasmid. Lentivirus-based expression vectors containing N- or C-terminally tagged FIZ1 were generated using the Gateway LR cloning system (Thermo Fisher Scientific). A previously described PCR mutagenesis method was used to generate the FIZ1 constructs containing the S380Δ and G364A mutations.[5] Chemically competent DH5α *E. coli* were transformed with the resulting plasmids, and plasmids were purified using and E.Z.N.A. Plasmid DNA Mini Kit (Omega Biotek).

Lentivirus Vector Production and Transduction

Plasmids of interest were normalized to a concentration of 20 ng/µL in 900 µL of AE Buffer and plated in 100 mm TC-treated cell culture dishes (Greiner). 108 µL of TransIT-LT1 (Mirus) was

added to 4.5 mL of OptiMEM (Gibco). Next, pCMV-VSVg and pCMV-d8.91 were added to a 4 ng/µL concentration and the OptiMEM solution was plated into the cell culture dish containing normalized plasmid of interest and allowed to incubate at room temperature for 20 min. HEK293T cells were then seeded into the dish at 18×10^6 cells per dish in 8.1 mL of DMEM (with FBS and PSG) and incubated at 37 °C and 5% CO_2 for 24 h. After 24 h, the media was exchanged for fresh DMEM and allowed to incubate at 37 °C and 5% CO_2 for an additional 24 h. After 48 h, the supernatant was harvested and centrifuged 1000 × *g* for 10 min. The virus was concentrated using Lenti-X Concentrator (Takara) according to manufacturer's suggestion and reconstituted at a 100X in complete cell culture media. Aliquots of 300 µL were then stored at −80 °C for later use.

To lentivirally transduce Jurkat cells, 6-well plates were coated using Retronectin (Takara Bio) according to the manufacturer's instructions. Jurkat cells were seeded at 3×10^5 cells per well in complete RPMI media, and 300 µL of virus suspension was added dropwise into each well. Cells were allowed to incubate for 72 h, then cells were split and seeded into a fresh 6-well plate in RPMI complete media supplemented with 78 ng/mL puromycin. Selection media was replaced every 2-3 days until the appearance of visibly selected cells began to grow. Cells were split and expanded for future use.

Stable cell lines expressing FIZ1, mCherry-FIZ1 construct or V5-FIZ1 constructs were generated based on a previously reported method.[6] Briefly, HEK293T cells were transfected with viral packaging vectors and a lentiviral delivery vector containing the desired FIZ1 expression cassette using Lipofectamine 3000 (Invitrogen). Virus containing supernatant was harvested 24 and 48 h post-transfection and filtered using a 0.45 µm syringe filter unit (Corning). HEK293T and HCT116 cells were treated with 4 µg/mL protamine sulfate (Thermo Fisher Scientific), infected with virus and selected with antibiotic to create stable cell lines.

Cell Lysis and Immunoblotting

Cells were plated 24 h before treatment. The collected cell pellet was lysed with in-house RIPA buffer (50 mM HEPES, 150 mM NaCl, 1% *v/v* NP-40, 1% *w/v* sodium deoxycholate, 0.1% *w/v* SDS, 10 mM glycerophosphate, 10 mM sodium pyrophosphate, protease inhibitor cocktail, phosphatase inhibitor cocktail, pH 7.4) containing 15 mM $MgCl_2$ and benzonase (MinneBio). The lysate was then sonicated and protein concentration determined for each sample *via* a Bradford

assay. Sample concentrations were normalized, and samples were denatured by addition of LDS supplemented with 50 mM DTT, followed by boiling at 90 °C for 5 min. Up to 40 µg of cell lysate was loaded onto a 4-12% NuPAGE Bis-Tris gel (Thermo Fisher Scientific), followed by SDS-PAGE with MES running buffer. Following transfer to a 0.45 µm PVDF membrane (Millipore), blocking was performed with 5% non-fat milk at room temperature for 30 min. The membrane was then incubated overnight at 4 °C with primary antibodies, washed with TBST and further incubated with fluorescent IRDye antibody (Licor) for 1-2 h at room temperature to visualize. The membrane was then imaged using the OdysseyCLx (Licor) imager or Chemidoc MP (BioRad).

Western blot data were quantified using Image Lab 6.1 software (BioRad) and comparisons of quantified western blot data were performed using Welch's t-test in Graphpad Prism (Dotmatics).

Nuclear-Cytosolic Fractionation

HEK293T cells were harvested, and the collected cell pellet was incubated for 30 min in hypotonic buffer (5 mM Tris, protease inhibitor cocktail, pH 7.5) with end-over-end rotation. Cell membranes were lysed via manual shearing by repeatedly drawing up and expunging with a 21 G needle (Fisher Scientific) and 1 mL syringe (Fisher Scientific). Complete lysis was confirmed by trypan blue staining of nuclei, and an aliquot was collected and subjected to further lysis with addition of 10X RIPA buffer (50 mM HEPES, 150 mM NaCl, 10% *v/v* NP-40, 1% *w/v* SDS, 10% *w/v* sodium deoxycholate). Intact nuclei were spun down and the supernatant collected as the cytosolic fraction, while the nuclear fraction was washed PBS. 10X RIPA buffer was added to the cytosolic fraction and the nuclear fraction was lysed via the addition of RIPA buffer (50 mM HEPES, 150 mM NaCl, 1% *v/v* NP-40, 1% *w/v* sodium deoxycholate, 0.1% *w/v* SDS, 10 mM glycerophosphate, 10 mM sodium pyrophosphate, protease inhibitor cocktail, pH 7.4) to an equal volume as the cytosolic fraction.

Flow cytometry analysis for mCherry-FIZ1 expression changes in HCT116 cells after 5-^{t}Bu-Thal (5) treatment

Cells were plated in 6-well plates, in triplicate for each condition, and treated for 16 h with respective inhibitors. Samples were harvested using trypsin, resuspended in sorting buffer (1X DPBS, 1mM EDTA, 25 mM HEPES, 1% FBS, final pH 7.3-7.5), and filtered through 35 µm cell strainer caps. Flow cytometry was performed on FACS Aria (BD Biosciences), and analysis was

done using the FlowJo software. The distribution graph was exported from FlowJo, while the bar graph of the triplicate experiment was processed in Prism software.

Generation of FIZ1 knock-in cell lines using CRISPR-Cas9 gene editing

gRNAs targeting the C-terminus region of the human *FIZ1* gene were designed using the CHOPCHOP website (http://chopchop.cbu.uib.no/). ssDNA oligonucleotides (Integrated DNA technologies) were annealed into dsDNA duplexes containing the guide sequences (5′-GCGGGGCATGGACTGACTGCC-3′) and (5’-GTCACGCTCAGCAACCTCTCC-3’) and assembled into the pX459 plasmid. The donor vector was constructed by assembling a synthetic DNA gene block (Integrated DNA Technologies) containing HaloTag7 followed by V5 and homology arms (250 nucleotides upstream and downstream) with the pSMART vector backbone by Gibson assembly. HEK293T, HCT116, and U2OS cells were transfected with donor and gRNA vectors using Lipofectamine 3000 (Invitrogen). 24 h after transfection, cells were selected using Puromycin for 48 h. To select a pool of cells positive for knock-in at the endogenous *FIZ1* locus, the puromycin selected cells were then treated with 50 nM Janelia Fluor® 646 HaloTag® Ligand (Promega) for 2 h, the HaloTag ligand containing media was then replaced and after 1 h, the cells were prepared for cell sorting and Janelia Fluor® 646 positive cells were sorted using either a BD FACSymphony S6 or BD FACSAria III cell sorter. The pooled Janelia Fluor® 646 positive cells were expanded and used for experiments or seeded as single cells into 96-well plates by serial dilution. To verify integration of the transgene, both pooled and clonal populations were validated by immunoblotting with anti-V5 antibody as well as PCR amplification of the integrated region using primers flanking the upstream and downstream homology arms of the donor sequence.

Chromatin Immunoprecipitation (ChIP) and Sequencing

HCT116 FIZ1-HaloTag-V5 knock-in cells were plated in 15 cm plates 24 h before treatment such that their confluence was 60% at the time of treatment. Cells were then treated for 12 hours with 5 µM *N*-benzylated 5-*t*-butyl thalidomide or 5 µM 5-*t*-butyl thalidomide. After 12 h, the cells were washed three times with PBS and fixed with 1% formaldehyde for 10 min, after which the reaction was quenched by the addition of glycine to a final concentration of 0.125 M. These fixed cells were then provided to the MSKCC Epigenetics Research Innovation Lab for processing. The cells were washed twice with PBS and cell pellets were resuspended in SDS buffer (100 mM NaCl, 50 mM Tris-HCl pH 8.0, 5 mM EDTA, 0.5% SDS, 1x protease inhibitor cocktail from Roche).

The resulting nuclei were spun down, resuspended in the immunoprecipitation buffer at 1 mL per 0.5 million cells (SDS buffer and Triton Dilution buffer (100 mM NaCl, 100 mM TrisHCl pH 8.0, 5 mM EDTA, 5% Triton X-100) mixed in 2:1 ratio with the addition of 1x protease inhibitor cocktail (Millipore Sigma, #11836170001) and processed on a Covaris LE220+ Focused-ultrasonicator to achieve an average fragment length of 200-300 bps with the following parameters: PIP=420, Duty Factor=30, CBP/Burst per sec=200, Time = 35min. Chromatin concentrations were estimated using the Pierce™ BCA Protein Assay Kit (ThermoFisher Scientific) according to the manufacturer's instructions. The immunoprecipitation reactions were set up in 1mL of immunoprecipitation buffer in Protein LoBind tubes (Eppendorf) and pre-cleared with 50 µL of Protein A/G magnetic beads (Cytiva) for 2 hours at 4°C. After pre-clearing, the samples were transferred into new Protein LoBind tubes and incubated overnight at 4°C with V5 (CST, #13202) or H3K4me3 (Epicypher, 13-0060) at a 1:50 (V5) or 1:100 (H3K4me3) dilution. The next day, 50 µL of BSA-blocked Protein A/G magnetic beads were added to the reactions and incubated for 2 hours at 4°C. The beads were then washed two times with low-salt washing buffer (150mM NaCl, 1% Triton X-100, 0.1% SDS, 2 mM EDTA, 20 mM TrisHCl pH8.0), two times with high-salt washing buffer (500mM NaCl, 1% Triton X-100, 0.1% SDS, 2 mM EDTA, 20mM TrisHCl pH8.0), two times with LiCl wash buffer (250 mM LiCl, 10 mM TrisHCl pH8.0, 1 mM EDTA, 1% Na-Deoxycholate, 1% IGEPAL CA-630) and one time with TE buffer (10 mM TrisHCl pH8.0, 1 mM EDTA). The samples were then reverse-crosslinked overnight in the elution buffer (1% SDS, 0.1 M $NaHCO_3$) and purified using the ChIP DNA Clean & Concentrator kit (Zymo Research) following the manufacturer's instructions. The recovered DNA fragments were quantified using a Qubit Flex fluorometer (ThermoFisher Scientific) and sent to the MSKCC Integrated Genomics Operation core facility for library preparation and sequencing. Immunoprecipitated DNA was quantified by PicoGreen, and the size was evaluated by Agilent BioAnalyzer. Illumina sequencing libraries were prepared using the KAPA EvoPrep Kit (Roche) according to the manufacturer's instructions with 0.2-5 ng input DNA and 14 cycles of PCR. Barcoded libraries were run on the NovaSeq 6000 in a PE100 run, using the NovaSeq 6000 S4 Reagent Kit (200 Cycles) (Illumina). An average of 20-30M paired reads were generated per sample.

ChIP-seq data analysis

Raw sequencing reads were trimmed and filtered for quality (Q>15) and adapter content using version 0.4.5 of TrimGalore (https://www.bioinformatics.babraham.ac.uk/projects/trim_galore) and running version 1.15 of cutadapt and version 0.11.5 of FastQC. Version 2.3.4.1 of bowtie2 (http://bowtie-bio.sourceforge.net/bowtie2/index.shtml) was employed to align reads to human assembly hg38, and alignments were deduplicated using MarkDuplicates in Picard Tools v2.16.0. Enriched regions were discovered using MACS2 (https://github.com/taoliu/MACS) with a p-value setting of 0.001, filtered for blacklisted regions (http://mitra.stanford.edu/kundaje/akundaje/release/blacklists/hg38-human/hg38.blacklist.bed.gz), and a peak atlas was created using +/- 250 bp around peak summits. The BEDTools suite (http://bedtools.readthedocs.io) was used to create normalized bigwig files. Version 1.6.1 of featureCounts (http://subread.sourceforge.net) was used to build a raw counts matrix, and DESeq2 was employed to calculate differential enrichment for all pairwise contrasts. Peak-gene associations were created by assigning all intragenic peaks to that gene, while intergenic peaks were assigned using linear genomic distance to transcription start sites (TSS). Motif signatures were obtained using Homer v4.5 (http://homer.ucsd.edu) on differentially enriched peak regions. Peak intersections from individual replicate comparisons were used to generate motif signatures relative to size and nucleotide composition-matched background sequences.

Total Proteomics

The total proteomics analysis workflow using TMTpro in Jurkat, MM.1S, and ESC (H9 WA09, WiCell) cells is reported in reference 7.[7]

Total proteomics analysis using MOLM13 and HCT116 cells were performed based on a previously reported method.[6] For the MOLM13 total proteomics, 10 mL of MOLM13 cells at a density of 2 x 10^5 cells/mL were plated per condition, for a total of 6 flasks. Samples were treated with 5-^{t}Bu-Thal (**5**) to a final concentration of 500 nM and retreated every 24 h. After 48 h, the cells were expanded to 30 mL of media, and after 72 h, the cells were collected. Cells were lysed with 600 μL of RIPA buffer (50 mM HEPES, 150 mM NaCl, 1% *v/v* NP-40, 1% *w/v* sodium deoxycholate, 0.1% *w/v* SDS, 10 mM glycerophosphate, 10 mM sodium pyrophosphate, protease inhibitor cocktail, phosphatase inhibitor cocktail, pH 7.4) containing 15 mM $MgCl_2$ and benzonase. The lysate was then sonicated, and protein concentration was determined for each

sample via Bradford assay. Sample concentrations were normalized to 2 mg/mL of protein, and 150 µg of protein was taken for each sample. TCEP was added to a concentration of 5 mM to each proteomics sample, and samples were incubated for 10 min at 55 °C. The lysate was then allowed to cool to room temperature, a freshly prepared chloroacetamide solution was added to a final concentration of 20 mM, and the samples were incubated at r.t. for 15 min, followed by chloroform/methanol precipitation of proteins from each sample. Samples were resuspended in 200 mM EPPS, pH 8.5, containing 0.1% RapiGest, and digested at 37 °C overnight with trypsin (100:1 protein:protease mass ratio). Tandem mass tag labeling of each sample was performed by adding the appropriate TMT 6plex reagent to a final acetonitrile concentration of 30% *v/v*. Following incubation at room temperature for 1 h, the reaction was quenched with hydroxylamine to a final concentration of 0.5% *v/v* for 15 min.

For the HCT116 total proteomics, HCT116 cells were plated in 10 cm plates 24 h before treatment, such that their final confluence was 80% at the time of treatment. Cells were then treated with 5 µM *N*-benzylated 5-*t*-butyl thalidomide, 5 µM 5-*t*-butyl thalidomide, or DMSO. After 6 h, the media was removed, and the cells were washed with PBS three times. After removing remaining PBS, the cells were scraped with 0.5 mL RIPA buffer containing 15 mM $MgCl_2$ and benzonase. The lysate was then sonicated, and protein concentration was determined for each sample via Bradford assay. Sample concentrations were normalized to 2 mg/mL of protein, and 150 µg of protein was taken for each sample. TCEP was added to a concentration of 5 mM to each proteomics sample, and samples were incubated for 10 min at 55 °C. The lysate was then allowed to cool to room temperature, a freshly prepared chloroacetamide solution was added to a final concentration of 20 mM, and the samples were incubated at r.t. for 15 min, followed by chloroform/methanol precipitation of proteins from each sample. Samples were resuspended in 200 mM EPPS, pH 8.5, containing 0.1% RapiGest, and 100 ug was taken for digestion at 37 °C overnight with trypsin (100:1 protein:protease mass ratio). Tandem mass tag labeling of each sample was performed by adding the appropriate TMT 11plex reagent to a final acetonitrile concentration of 30% *v/v*. Following incubation at room temperature for 1 h, the reaction was quenched with hydroxylamine to a final concentration of 0.5% *v/v* for 15 min.

For both experiments, TMT labelled samples were pooled together at a 1:1 ratio. The sample was vacuum centrifuged to remove acetonitrile and then subjected to C18 solid phase extraction (SPE)

using a Sep-Pak cartridge (50 mg, Sep-Pak, Waters). The dried TMT-labeled sample was resuspended in 100 µL of 10 mM NH_4HCO_3, pH 8.0, and off-line fractionated using basic pH reversed-phase High-performance liquid chromatography (HPLC) (Agilent LC1260 Infinity II, equipped with a degasser and wavelength detector (set at 214 nm).[7] Peptides were subjected to a 60 min linear gradient from 8% to 40% acetonitrile in 10 mM NH_4HCO_3, pH 8.0, at a flow rate of 0.6 mL/min over an Aeris peptide xb C18 column (Phenomenex; 2.6 µm, 100 Å, 4.6 mm ID and 250 mm in length). The 96 resulting fractions were then pooled in a non-continuous manner into 24 fractions (as outlined in Figure S5 of Paulo *et al.*).[9] Fractions were vacuum centrifuged to near dryness. Each consolidated fraction was desalted via Stage Tip, dried again *via* vacuum centrifugation, and reconstituted in 5% acetonitrile, 1% formic acid for LC-MS/MS processing.

Proteomics – Data Collection

Mass spectrometry data were acquired using an Orbitrap Eclipse Tribrid mass spectrometer (Thermo Fisher Scientific, San Jose, CA) connected to an UltiMate 3000 RSLCnano UHPLC (Thermo Fisher Scientific, San Jose, CA). Peptides were separated on a 100 µm inner diameter microcapillary column packed in-house with ~ 30 cm of HALO Peptide ES-C18 resin (2.7 µm, 160 Å, Advanced Materials Technology, Wilmington, DE) with a gradient consisting of 5 %-40 % (ACN, 0.1% FA) over a 125 min (run at ~400 nL/min. 3/10 of each fraction was loaded onto the column for analysis. Proteome analysis used Multi-Notch MS3-based TMT quantification to reduce ion interference compared to MS2 quantification, combined with the FAIMS Pro Interface (using previously optimized 3 CV parameters for TMT multiplexed samples), and integrated with newly implemented Real-Time Search analysis software.[2,9–12] The scan sequence began with an MS1 spectrum (Orbitrap analysis; resolution 120,000 at 200 Th; mass range 400−1500 *m/z*; maximum injection time 50 ms; automatic gain control (AGC) target 4x105). For MS2 analysis, precursors were selected based on a cycle time of 1.25 sec/CV method (FAIMS CV=-40/-60/-80). MS2 analysis consisted of collision-induced dissociation (quadrupole ion trap analysis; Rapid scan rate; AGC 1.0x104; isolation window 0.5 Th; normalized collision energy (NCE) 35; maximum injection time 35 ms). Monoisotopic peak assignment was used, and previously interrogated precursors were excluded using a dynamic window (120 s ±10 ppm). Following the acquisition of each MS2 spectrum, a synchronous-precursor-selection (SPS) API-MS3 scan was collected on the top 10 most intense ions b- or y-ions matched by the online search algorithm in the associated MS2 spectrum.[12,13] MS3 precursors were fragmented by high-energy collision-induced dissociation

(HCD) and analyzed using the Orbitrap (NCE 65; AGC 2.5x105; maximum injection time 200 ms, resolution was 50,000 at 200 Th). Closeout was set at two peptides per protein per fraction, so that MS3s were no longer collected for proteins having two peptide spectrum matches (PSMs) that passed quality filters.[12]

Proteomics – Data Analysis

Mass spectra were processed using a Comet-based (2020.01 rev. 4) software pipeline.[14,15] Spectra were first converted to mzXML, and monoisotopic peaks were re-assigned using Monocle software.[16] MS2 spectra were matched with peptide sequences with a composite sequence database including the Human Reference Proteome UniProt database (Proteome ID: UP000005640, release 2022_03, containing SwissProt canonical and isoform entries), as well as sequences of common contaminants. This database was concatenated with one composed of all protein sequences in the reverse order. Analysis was performed using a 50 ppm precursor ion tolerance. Static modifications included TMT tags (+229.163 Da) on lysine residues and peptide N termini, along with carbamidomethylation of cysteine residues (+57.021 Da). Oxidation of methionine residues (+15.995 Da) was set as a variable modification. Peptide-spectrum matches (PSMs) were adjusted to a 1% false discovery rate (FDR).[17] PSM filtering was performed using a linear discriminant analysis, while considering the following parameters: Comet log expect, different sequence delta Comet log expect, missed cleavages, peptide length, charge state, precursor mass accuracy, and fraction of ions matched.[18] For protein-level comparisons, PSMs were identified, quantified, and collapsed to a 1% peptide false discovery rate (FDR) and then collapsed further to a final protein-level FDR of 1%.[19] To generate the smallest set of proteins required to account for all observed peptides, the principles of parsimony were applied. For TMT-based reporter ion quantitation, the summed signal-to-noise (S:N) ratio for each TMT channel was first extracted based on the closest matching centroid to the expected mass of the TMT reporter ion (integration tolerance of 0.003, or 0.03 for 6plex). Isotopic impurities of the different TMT reagents provided by the manufacturer's specifications were used to adjust reporter ion intensities. Proteins were quantified by summing reporter ion signal-to-noise measurements across all matching PSMs, resulting in a ''summed signal-to-noise'' measurement. PSMs with poor quality, MS3 spectra missing reporter ion channels, isolation specificity below 0.5, TMT reporter summed signal-to-noise ratio less than 10 per condition, or no MS3 spectra were excluded from quantification.

Protein quantification values were exported for further analysis in Microsoft Excel and Perseus.[20] Each reporter ion channel was summed across all quantified proteins and normalized, assuming equal protein loading across all samples. The maximum and minimum TMT ratio that could be quantified was capped at 100-fold. In Perseus, a two-way Welch's t-test analysis was performed to compare two datasets, using the S0 parameter of 0.895 (a minimal fold change cutoff), and 1% correction for multiple comparisons was achieved using the permutation-based FDR method, both of which are built-in functions of Perseus software. Supplementary Tables list all quantified proteins and the TMT reporter ratio associated with control channels used for quantitative analysis.

Protein quantification values were exported for further analysis in Microsoft Excel and Perseus.[21] Each reporter ion channel was summed across all quantified proteins and normalized, assuming equal protein loading across all samples. The maximum and minimum TMT ratio that could be quantified was capped at 100-fold. In Perseus, a two-way Welch's t-test analysis was performed to compare two datasets, using the S0 parameter of 0.895 (a minimal fold change cutoff), and 1% correction for multiple comparisons was achieved using the permutation-based FDR method, both of which are built-in functions of Perseus software.

Supplementary Tables list all quantified proteins and the TMT reporter ratio associated with control channels used for quantitative analysis.

Immunoprecipitation-Mass Spectrometry Sample Preparation

Pooled HCT116 FIZ1-HaloTag-V5 knock-in cells were plated in 15 cm plates 24 h before treatment such that their final confluence was 80% at the time of treatment. Cells were then treated with 5 µM *N*-benzylated 5-*t*-butyl thalidomide or 5 µM 5-*t*-butyl thalidomide. After 6 h, the media was removed, and the cells were washed with PBS three times. After removing remaining PBS, the cells were scraped with 1 mL of NP-40 buffer (50 mM HEPES, 150 mM NaCl, 0.5% v/v NP-40, protease inhibitor cocktail, phosphatase inhibitor cocktail, pH 7.4) containing 15 mM $MgCl_2$ and benzonase (MinneBio). The lysate was then sonicated and protein concentration determined for each sample via Bradford assay. Protein concentration was normalized to 12 mg/mL and 10 µL of lysate was diluted in 40 µL of lysis buffer for Western blot analysis. 10 µL of anti-V5 nanobody beads (Chromotek) were distributed for each sample and washed twice with lysis buffer. 1 mL of lysate was added to the beads and incubated for 2 h at 4 °C with rotation. The flowthrough

was then collected by centrifuging the beads with a benchtop centrifuge, and 10 μL of flowthrough was diluted in 40 μL of lysis buffer for Western blot analysis. The beads were then washed twice with 1 mL of wash buffer 1 (50 mM HEPES, 150 mM NaCl, 0.5% v/v NP-40, pH 7.4) and washed twice again with 1 mL of wash buffer 2 (50 mM HEPES, 150 mM NaCl, pH 7.4). The beads were then transferred to hydrophilic PTFE membrane filter cups with the aid of wash buffer 2 and incubated with 100 μL of 1X NuPAGE LDS sample buffer (Invitrogen), diluted in wash buffer 1, for 10 min at 60 °C and eluted by centrifugation for 1 min at 2500 rcf. 10 μL of the elution was taken for western blot analysis, and the rest was further processed for TMT-MS analysis.

The eluted samples were subjected to reduction and alkylation. In brief, TCEP was added to a concentration of 5 mM to each sample, and samples were incubated for 15 min at 55ºC. The lysate was then allowed to cool to room temperature, freshly prepared chloroacetamide solution was added to a final concentration of 20 mM, and the samples were incubated at r. t. for 15 min. Samples were then subjected to SP3 protein cleanup (PMID: 29565595). The SP3 beads were then resuspended in 30 μL of digestion buffer (200 mM EPPS, 0.1% RapiGest, pH 8.5), sonicated briefly, and digested at 37 °C overnight with 0.5 μg of trypsin. Following digestion, the tubes were placed on a magnetic rack, and the supernatant was taken for further processing. Tandem mass tag labeling of each sample was performed using 5 μL of 20 ng/mL TMT 6-plex stock (Thermo Scientific) in acetonitrile. Following incubation at room temperature for 1 h, the reaction was quenched with hydroxylamine to a final concentration of 1% v/v for 15 min. The TMT-labelled samples were pooled together at a 1:1 ratio. The sample was then subjected to C18 solid-phase extraction (SPE) (10 mg, Sep-Pak, Waters).

Live Cell Confocal Microscopy

48 h prior to imaging, cells were plated onto 33 mm glass-bottom dishes (No. 1.5, 14 mM glass diameter, MatTek) pretreated with poly-L-lysine. Prior to imaging, cell media was changed to phenol red free medium (FluoroBrite DMDM, Thermo Fisher) supplemented with 10% fetal bovine serum. Cells were then imaged using a Yokogawa spinning disk confocal on an inverted Nikon Ti fluorescence microscope equipped with a Plan Apo TIRF 60x/1.49 N.A. objective lens (oil immersion). Imaging data was further processed using Fiji.

GFP Degradation Flow Cytometry Assay

Test compounds (10 mM stock in DMSO) were serially diluted 10-fold to create an 8-point dilution spanning 1 pM – 10 μM (final assay concentration). In the rescue experiments, additional conditions with 1 μM of compound and either carfilzomib (1 μM) or MLN4924 (1 μM) were tested. Jurkat cells were added to 96 well U-bottom assay plates (Falcon) at 2×10^4 cells per well and treated with compounds for 18 h at 37 °C and 5% CO_2. Cells were acquired on an Attune NxT (Invitrogen). Data were analyzed using FlowJo 10.8.1 (BD).

Gene Fragments

Gene fragment name	Sequence
ZF1:<br>Artichoke_IKZF3_130to145_FIZ1_23to45_IKZF3_169to189	GGCTACCGTCTCACCATGTCCGGATTCAATGTCTTAATGGTTCATAAGCGAAGCCATACTGGTGAACGCCCATTTCACTGCAGTGAATGTGGCAAGAGCTTCCGCTACCGCTCAGACCTGCGGCGCCACTTTGCCCGGCACACAGGGGAAAAACCTTTTAAGTGTCACCTCTGCAACTATGCATGCCAAAGAAGAGATGCGCTCACGCGTGCTGAGAGACGAGTAGC
ZF2:<br>Artichoke_IKZF3_130to145_FIZ1_51to73_IKZF3_169to189	GGCTACCGTCTCACCATGTCCGGATTCAATGTCTTAATGGTTCATAAGCGAAGCCATACTGGTGAACGCCCACACGCATGTCCGCGCTGCGGCAAGGGTTTCAAGCACAGCTTCAACCTAGCCAACCACCTGCGCTCGCACACAGGGGAAAAACCTTTTAAGTGTCACCTCTGCAACTATGCATGCCAAAGAAGAGATGCGCTCACGCGTGCTGAGAGACGAGTAGC
ZF7:<br>Artichoke_IKZF3_130to145_FIZ1_331to352_IKZF3_169to189	GGCTACCGTCTCACCATGTCCGGATTCAATGTCTTAATGGTTCATAAGCGAAGCCATACTGGTGAACGCCCATACCAGTGCGACTGCGGGACCTTCTTTGCGTCGGCCGCGGCCCTGGCCAGTCACCTGGAGGCCCACACAGGGGAAAAACCTTTTAAGTGTCACCTCTGCAACTATGCATGCCAAAGAAGAGATGCGCTCACGCGTGCTGAGAGACGAGTAGC
ZF8:<br>Artichoke_IKZF3_130to145_FIZ1_358to381_IKZF3_169to189	GGCTACCGTCTCACCATGTCCGGATTCAATGTCTTAATGGTTCATAAGCGAAGCCATACGGCTGCGGCCACTGCGGGGCTCTGTACGCGGCGCTGGCCGCCTTGGAGGAGCACCGGCGGGTCAGCCACACAGGGGAAAAACCTTTTAAGTGTCACCTCTGCAACTATGCATGCCAAAGAAGAGATGCGCTCACGCGTGCTGAGAGACGAGTAGC

ZF8_truncated_flank<br><br>Artichoke_IKZF3_130to145_FIZ1_358to381_IKZF3_170to189	GGCTACCGTCTCACCATGTCCGGATTCAATGTCTTAATGGTTCATAAGCGAAGCCATACTGGTGAACGCCCATACGGCTGCGGCCACTGCGGGGCTCTGTACGCGGCGCTGGCCGCCTTGGAGGAGCACCGGCGGGTCAGCCACGGGGAAAAACCTTTTAAGTGTCACCTCTGCAACTATGCATGCCAAAGAAGAGATGCGCTCACGCGTGCTGAGAGACGAGTAGC
ZF8_H381Δ:<br><br>Artichoke_IKZF3_130to145_FIZ1_358to380_IKZF3_169to189	GGCTACCGTCTCACCATGTCCGGATTCAATGTCTTAATGGTTCATAAGCGAAGCCATACTGGTGAACGCCCATACGGCTGCGGCCACTGCGGGGCTCTGTACGCGGCGCTGGCCGCCTTGGAGGAGCACCGGCGGGTCAGCACAGGGGAAAAACCTTTTAAGTGTCACCTCTGCAACTATGCATGCCAAAGAAGAGATGCGCTCACGCGTGCTGAGAGACGAGTAGC
ZF8_S380Δ:<br><br>Artichoke_IKZF3_130to145_FIZ1_358to379_and_381_IKZF3_169to189	GGCTACCGTCTCACCATGTCCGGATTCAATGTCTTAATGGTTCATAAGCGAAGCCATACTGGTGAACGCCCATACGGCTGCGGCCACTGCGGGGCTCTGTACGCGGCGCTGGCCGCCTTGGAGGAGCACCGGCGGGTCCACACAGGGGAAAAACCTTTTAAGTGTCACCTCTGCAACTATGCATGCCAAAGAAGAGATGCGCTCACGCGTGCTGAGAGACGAGTAGC
ZF10:<br><br>Artichoke_IKZF3_130to145_FIZ1_442to464_IKZF3_169to189	GGCTACCGTCTCACCATGTCCGGATTCAATGTCTTAATGGTTCATAAGCGAAGCCATACTGGTGAACGCCCATTCCCGTGCCTGGAGTGCGGCAAGTTCTTCCGCCACGAGTGCTACCTCAAGCGCCACCGGCTGCTGCACACAGGGGAAAAACCTTTTAAGTGTCACCTCTGCAACTATGCATGCCAAAGAAGAGATGCGCTCACGCGTGCTGAGAGACGAGTAGC
ZF11:<br><br>Artichoke_IKZF3_130to145_FIZ	GGCTACCGTCTCACCATGTCCGGATTCAATGTCTTAATGGTTCATAAGCGAAGCCATACTGGTGAACGCCCATTCCCTTGCCACATCTGCGGCAAGGGCTTCATCACGCTCAGCAACCTCTCCAGGCACCTGAAGCTGCACACAGGGGAAAAACCTTTTAAGTGTCACCTCTGCAAC

1_470to492_IKZF3_169to189	TATGCATGCCAAAGAAGAGATGCGCTCACGCGTGCTGAGAGACGAGTAGC
Artichoke_IKZF3_130to189	GGCTACCGTCTCACCATGTCCGGATTCAATGTCTTAATGGTTCATAAGCGAAGCCATACTGGTGAACGCCCATTCCAGTGTAATCAGTGTGGGGCATCTTTTACTCAGAAAGGTAACCTCCTCCGCCACATTAAACTGCACACAGGGGAAAAACCTTTTAAGTGTCACCTCTGCAACTATGCATGCCAAAGAAGAGATGCGCTCACGCGTGCTGAGAGACGAGTAGC
Artichoke_HEIP_130to189	GGCTACCGTCTCACCATGTCCGGATTCAATGTCTTAATGGTTCATAAGCGAAGCCATACTGGTGAACGCCCATTCCATTGTGAGATCTGTGGGGCACCATTTACTCAGAAAGGTAACCTCCTCCGCCACATTAAACTGCACACAGGGGAAAAACCTTTTAAGTGTCACCTCTGCAACTATGCATGCCAAAGAAGAGATGCGCTCACGCGTGCTGAGAGACGAGTAGC
Artichoke_AEVK_130to189	GGCTACCGTCTCACCATGTCCGGATTCAATGTCTTAATGGTTCATAAGCGAAGCCATACTGGTGAACGCCCATTCGCTTGTGAAGTGTGTGGGGCAAAATTTACTCAGAAAGGTAACCTCCTCCGCCACATTAAACTGCACACAGGGGAAAAACCTTTTAAGTGTCACCTCTGCAACTATGCATGCCAAAGAAGAGATGCGCTCACGCGTGCTGAGAGACGAGTAGC

Computational Methods

MD simulations to relax the models

Molecular dynamics (MD) simulations were performed using AMBER22 to prepare and relax the system.[22] The initial protein-ligand complex was parameterized using the ff14SB force field for the protein and GAFF2 for the ligand, with partial charges derived via the RESP method. The system was solvated in a TIP3P water box with a buffer of 10 Å and neutralized with counter ions. Two stages of minimization were conducted: (i) 5000 steps with 500 kcal/mol·$Å^2$ restraints on backbone atoms, and (ii) 5000 steps with 2 kcal/mol·$Å^2$ restraints. The system was then heated in six incremental steps from 0 K to 300 K over 50 ps for each step, using Langevin dynamics with a collision frequency of 5 ps^{-1}. Equilibration was performed in the NPT ensemble for 4 ns, with decreasing restraints from 30 kcal/mol·$Å^2$ to zero. Periodic boundary conditions and a nonbonded cutoff of 8.0 Å were applied throughout, and trajectories were saved every 25 ps for subsequent analyses.

Representative frame selection

Clustering of molecular dynamics (MD) trajectories in UCSF Chimera is performed using the MD Movie tool to identify conformational states based on structural similarity.[23] Trajectory frames are grouped into clusters using RMSD as a metric, typically focusing on specific regions such as the protein backbone or ligand-binding site. The clustering algorithm ranks clusters by population size, and the most representative frames—those closest to the centroid of each cluster—are selected.

^{1}H NMR of 5-(*tert*-butyl)-2-(1-methyl-2,6-dioxopiperidin-3-yl)isoindoline-1,3-dione (6)

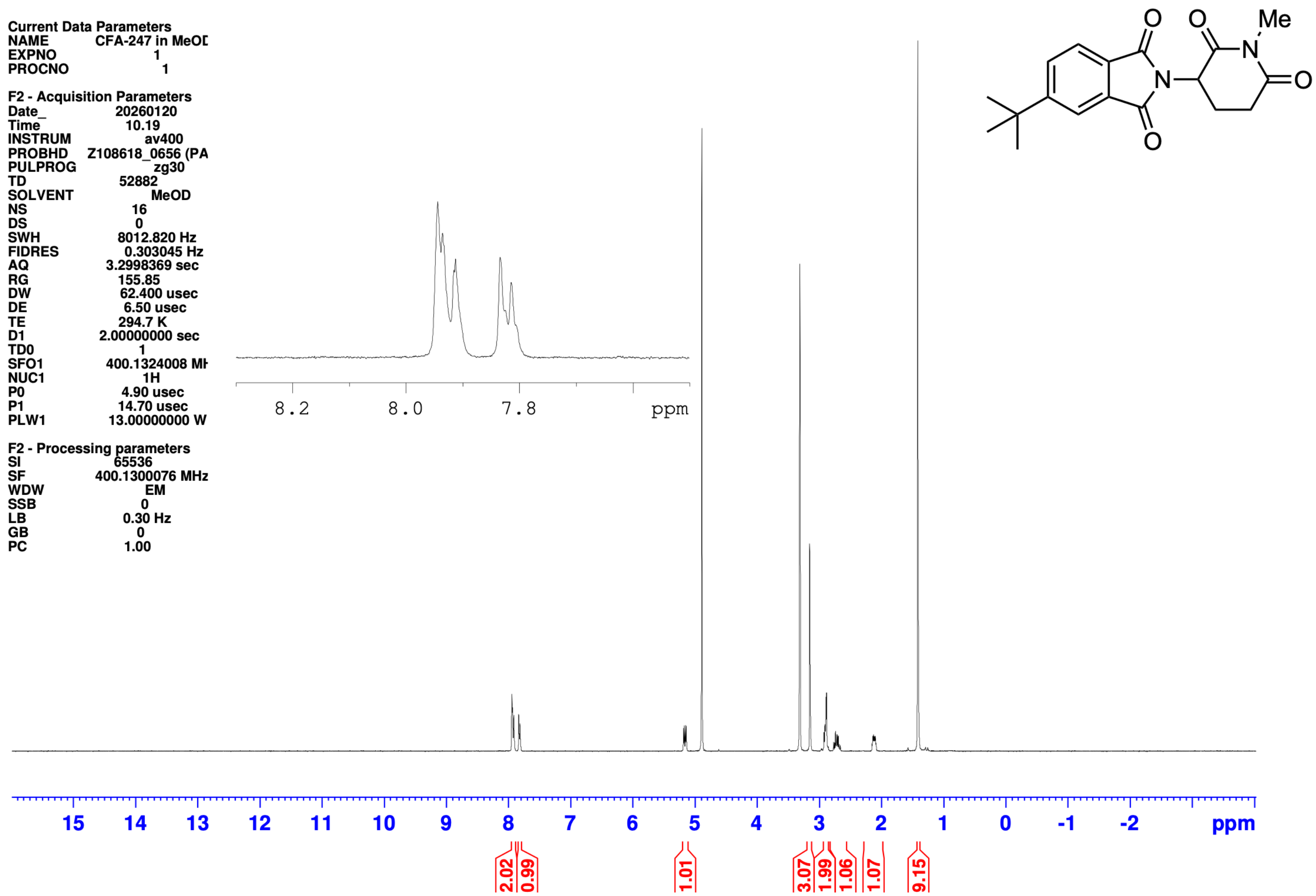

^{13}C NMR of 5-(*tert*-butyl)-2-(1-methyl-2,6-dioxopiperidin-3-yl)isoindoline-1,3-dione (6)

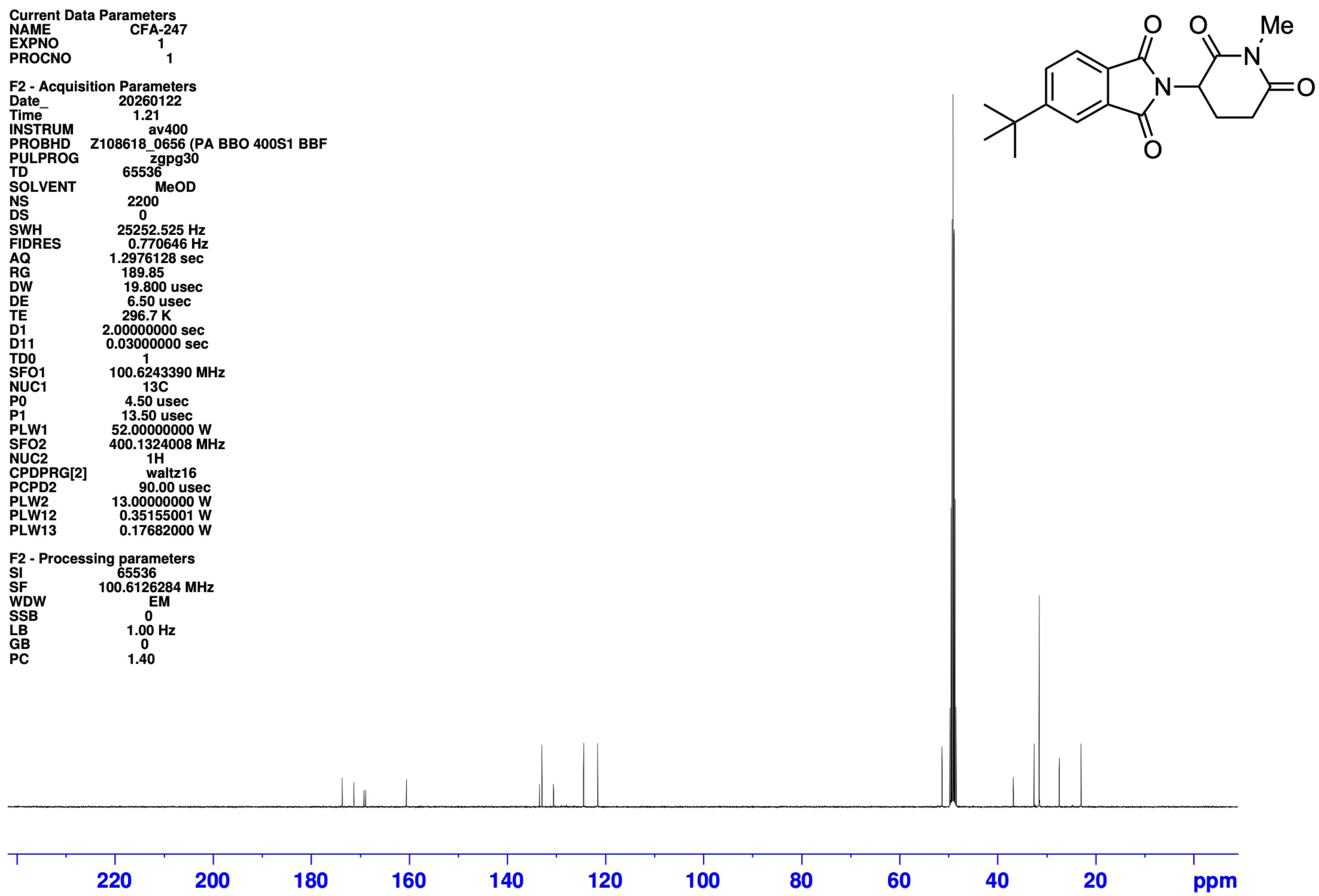

^{1}H NMR of 2-(1-benzyl-2,6-dioxopiperidin-3-yl)-5-(*tert*-butyl)isoindoline-1,3-dione (7)

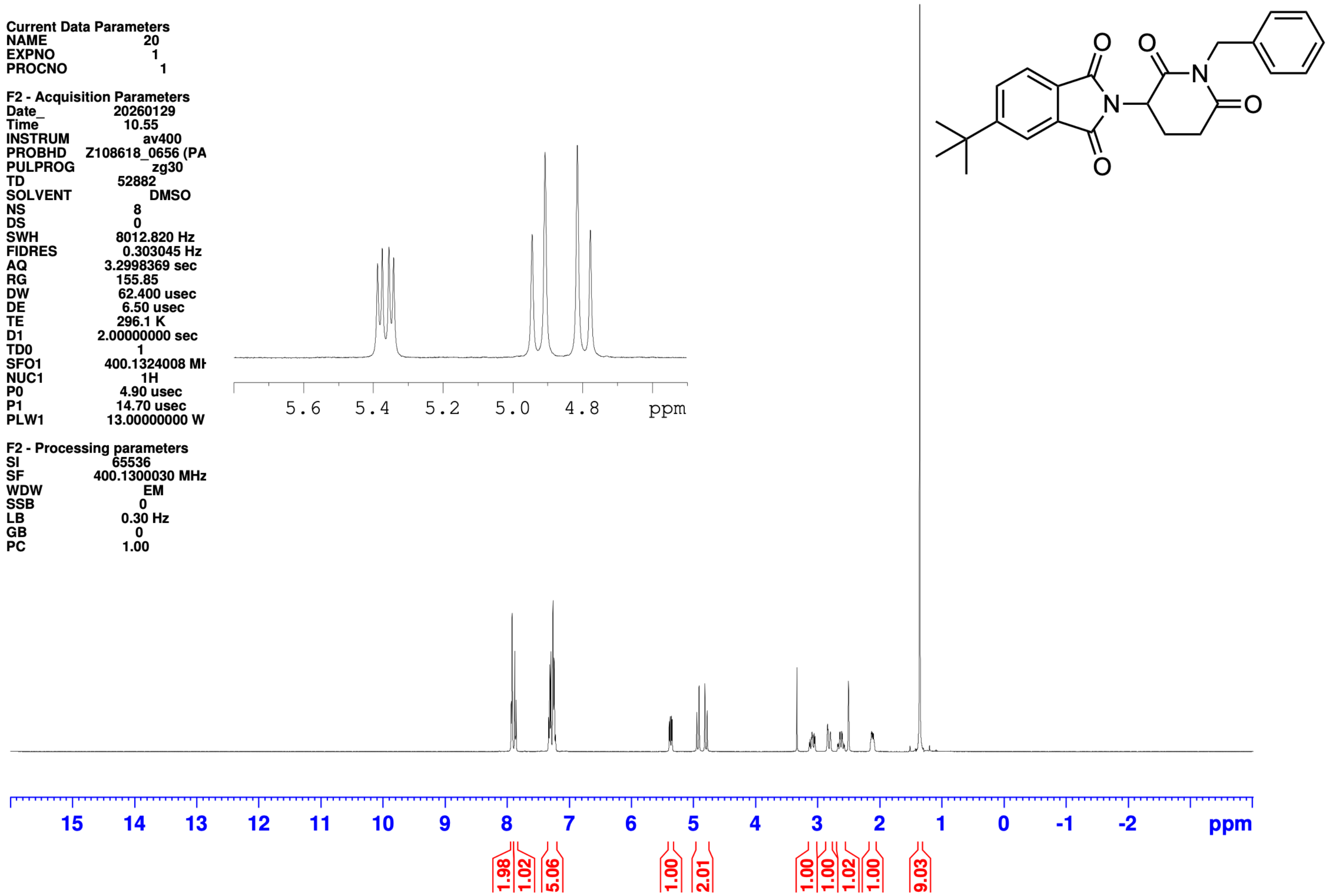

^{13}C NMR of 2-(1-benzyl-2,6-dioxopiperidin-3-yl)-5-(*tert*-butyl)isoindoline-1,3-dione (7)

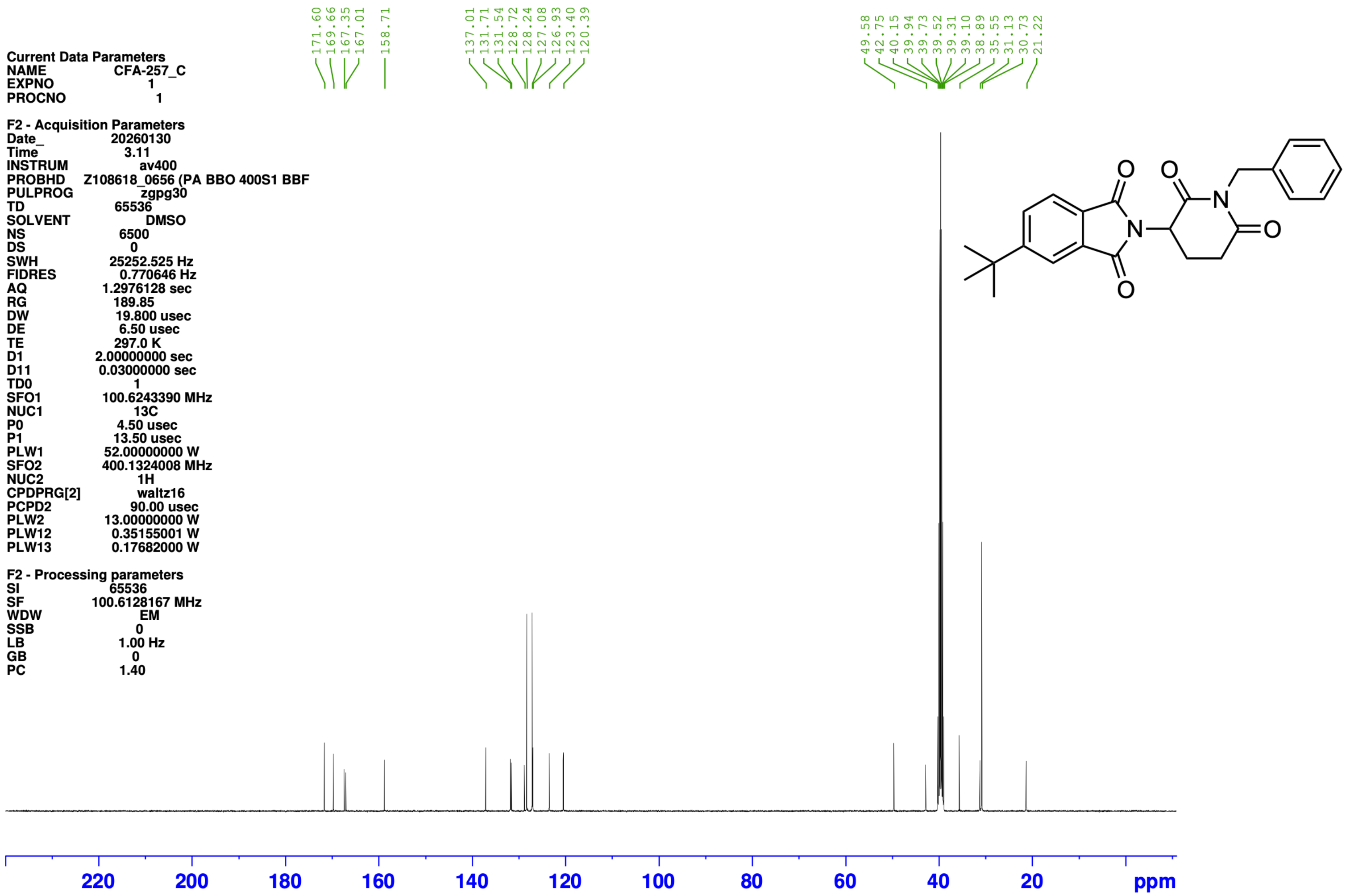

^{1}H NMR of 5-(cyclopropylamino)-2-(2,6-dioxopiperidin-3-yl)isoindoline-1,3-dione (8)

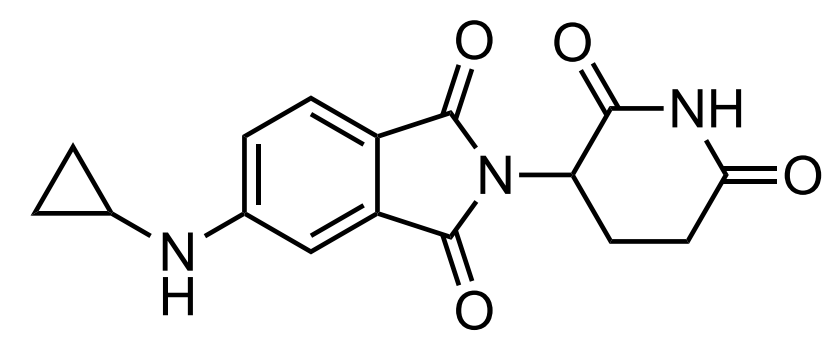

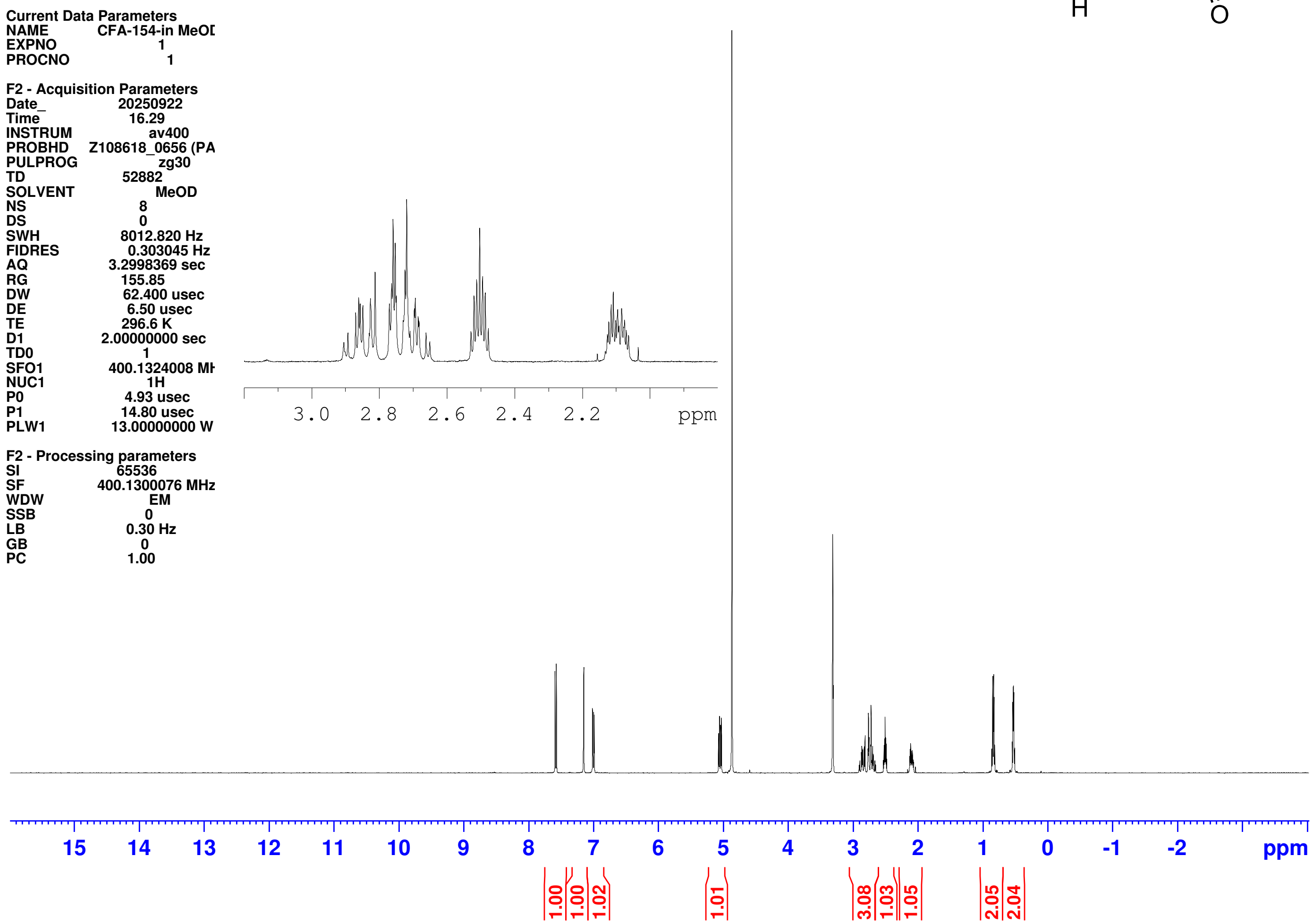

^{13}C NMR of 5-(cyclopropylamino)-2-(2,6-dioxopiperidin-3-yl)isoindoline-1,3-dione (8)

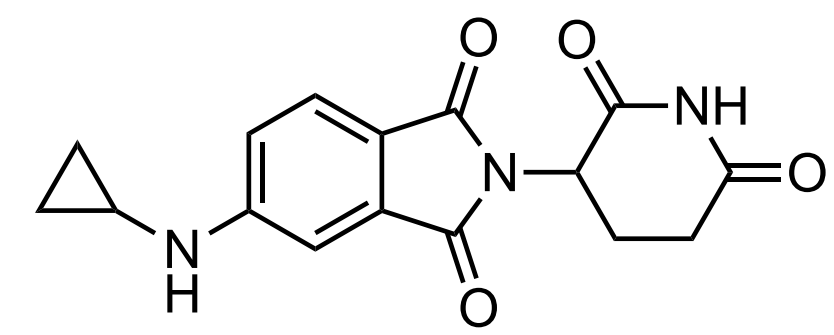

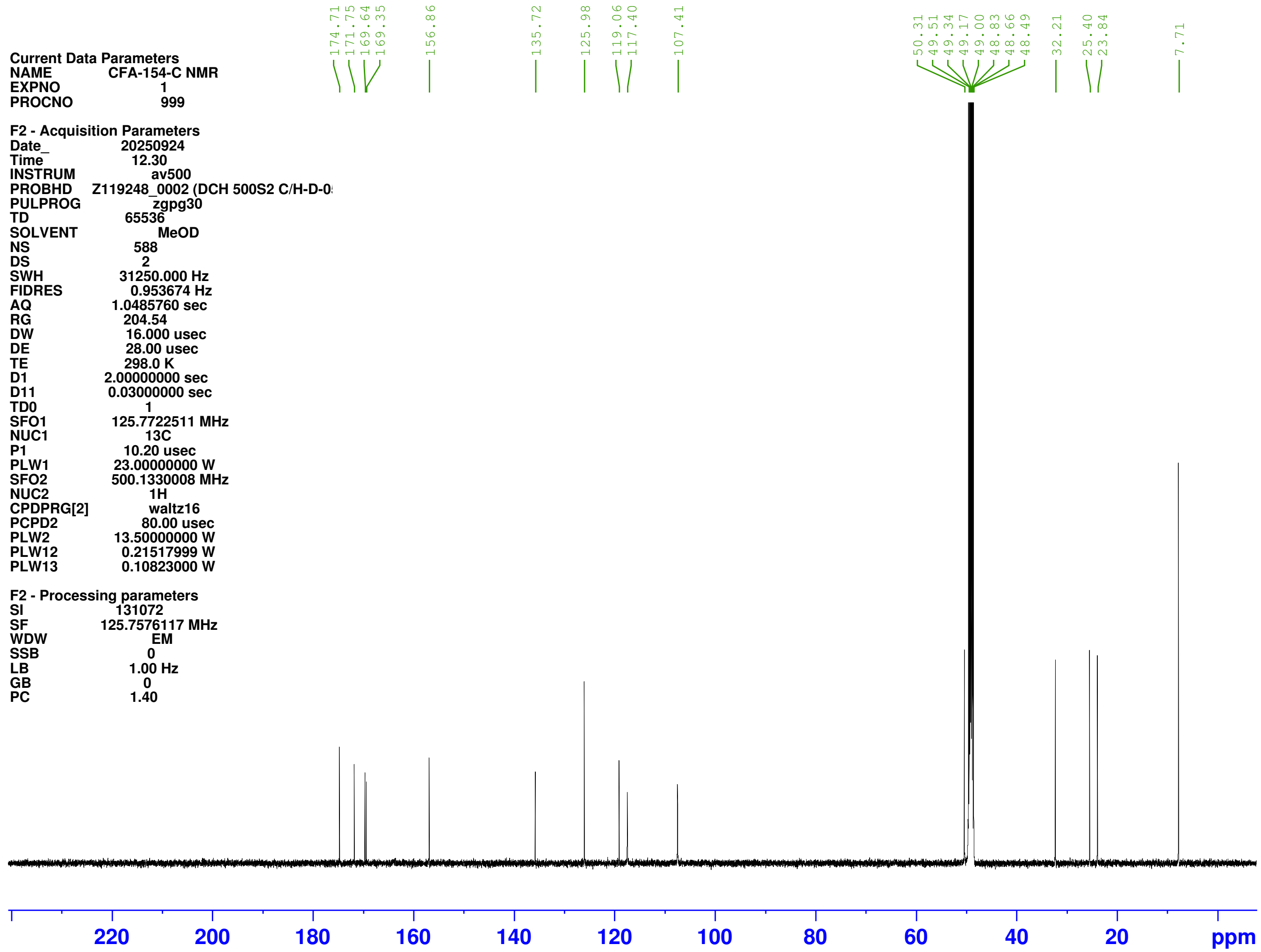

^{1}H NMR of 4-(cyclopropylamino)-2-(2,6-dioxopiperidin-3-yl)isoindoline-1,3-dione (9)

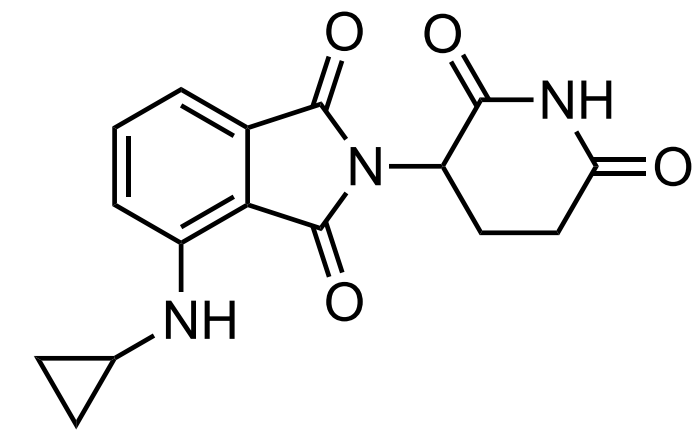

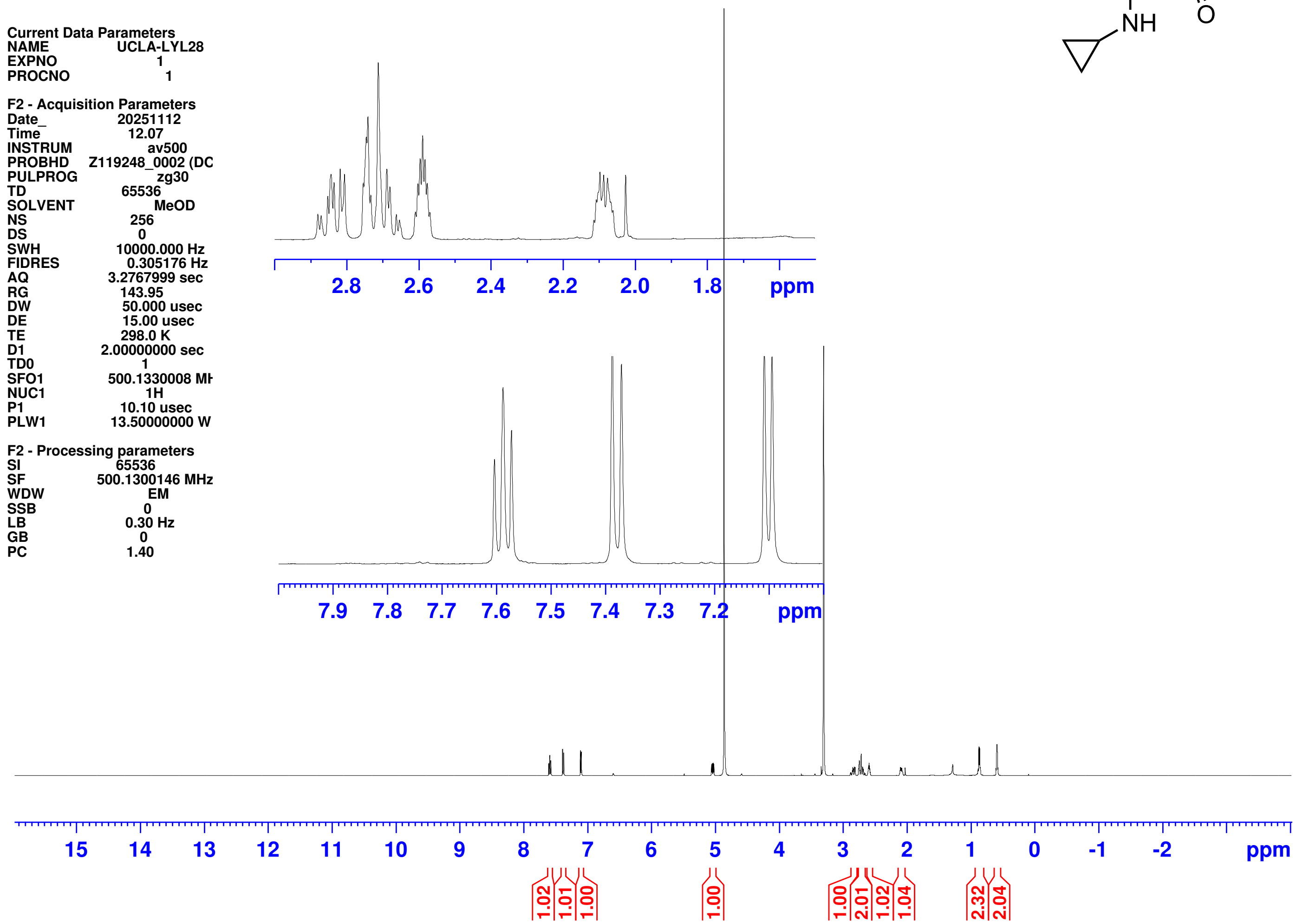

^{13}C NMR of 4-(cyclopropylamino)-2-(2,6-dioxopiperidin-3-yl)isoindoline-1,3-dione (9)

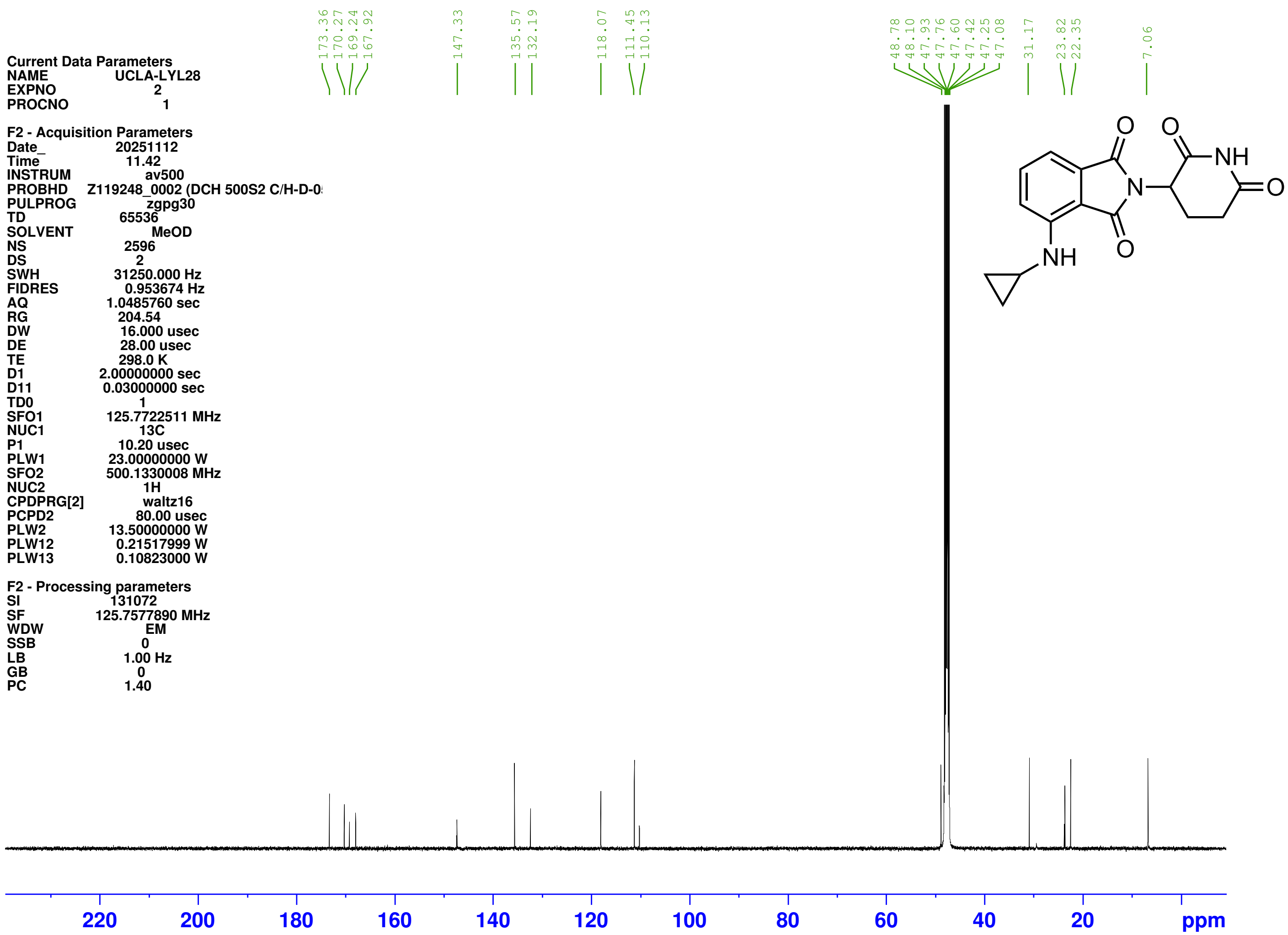

HPLC traces for 5-(*tert*-butyl)-2-(1-methyl-2,6-dioxopiperidin-3-yl)isoindoline-1,3-dione (6)

SHIMADZU LabSolutions **Analysis Report**

<Sample Information>

Sample Name	: CFA-247(30-38)-1		
Sample ID	:		
Data Filename	: Batch purity Fatma_1162026_005.lcd		
Method Filename	: Fatma_Standard_5-95MeCN1minhold_10mingrad_5minhold_0.1FA_1mlmin.lcm		
Batch Filename	: Batch purity Fatma.lcb		
Vial #	: 1-29	Sample Type	: Unknown
Injection Volume	: 1 uL		
Date Acquired	: 1/16/2026 12:21:38 PM	Acquired by	: System Administrator
Date Processed	: 1/16/2026 12:54:40 PM	Processed by	: System Administrator

<Chromatogram>

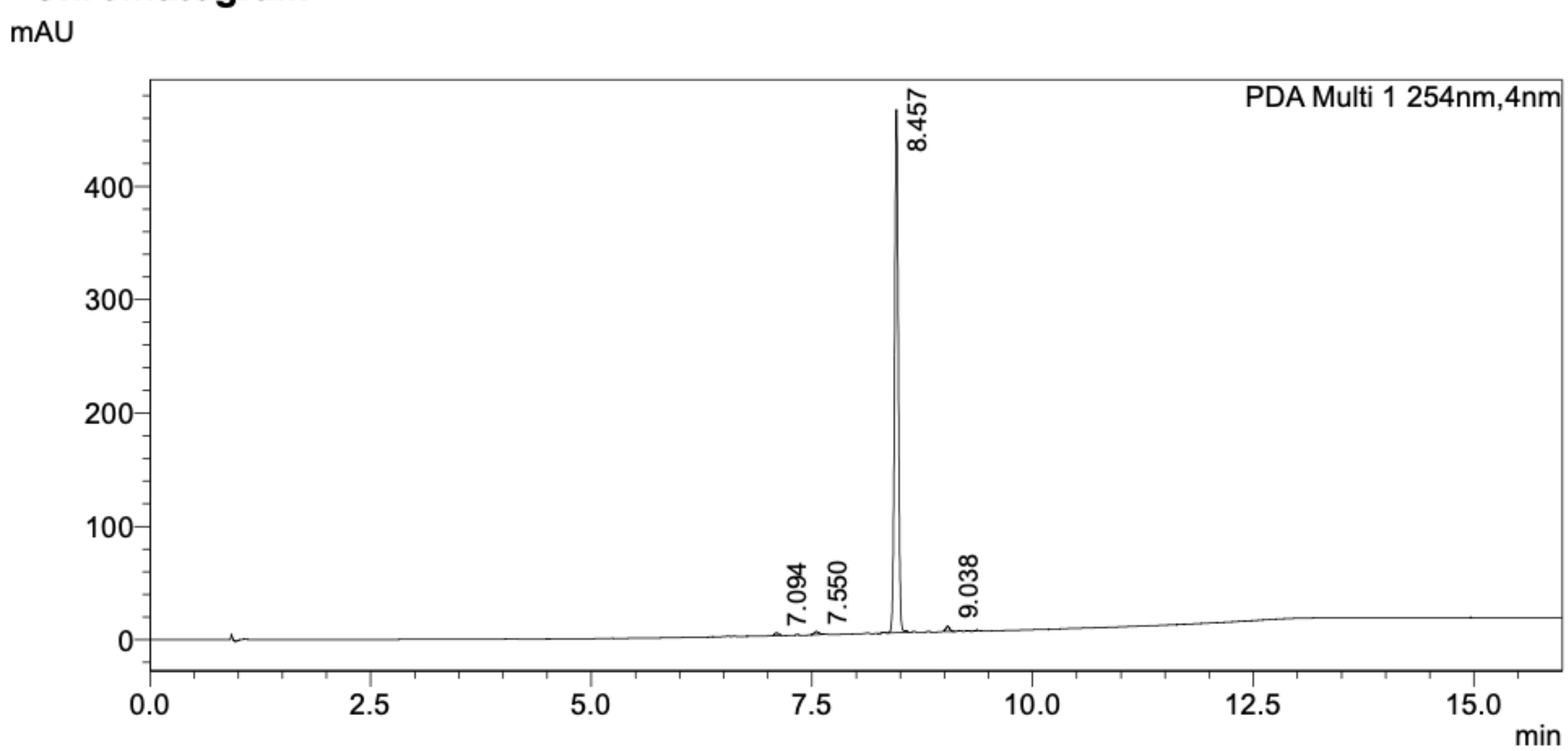

<Peak Table>

PDA Ch1 254nm

Peak#	Ret. Time	Area	Height	Unit	Mark	Area%
1	7.094	8036	2338		M	0.588
2	7.550	10729	2733		M	0.784
3	8.457	1336031	446796		M	97.678
4	9.038	12992	4515		M	0.950
Total		1367789	456382			100.000

SHIMADZU LabSolutions Analysis Report

<Sample Information>

Sample Name	: CFA-247(30-38)-1		
Sample ID	:		
Data Filename	: Batch purity Fatma_1162026_005.lcd		
Method Filename	: Fatma_Standard_5-95MeCN1minhold_10mingrad_5minhold_0.1FA_1mlmin.lcm		
Batch Filename	: Batch purity Fatma.lcb		
Vial #	: 1-29	Sample Type	: Unknown
Injection Volume	: 1 uL		
Date Acquired	: 1/16/2026 12:21:38 PM	Acquired by	: System Administrator
Date Processed	: 1/16/2026 12:54:40 PM	Processed by	: System Administrator

<Chromatogram>

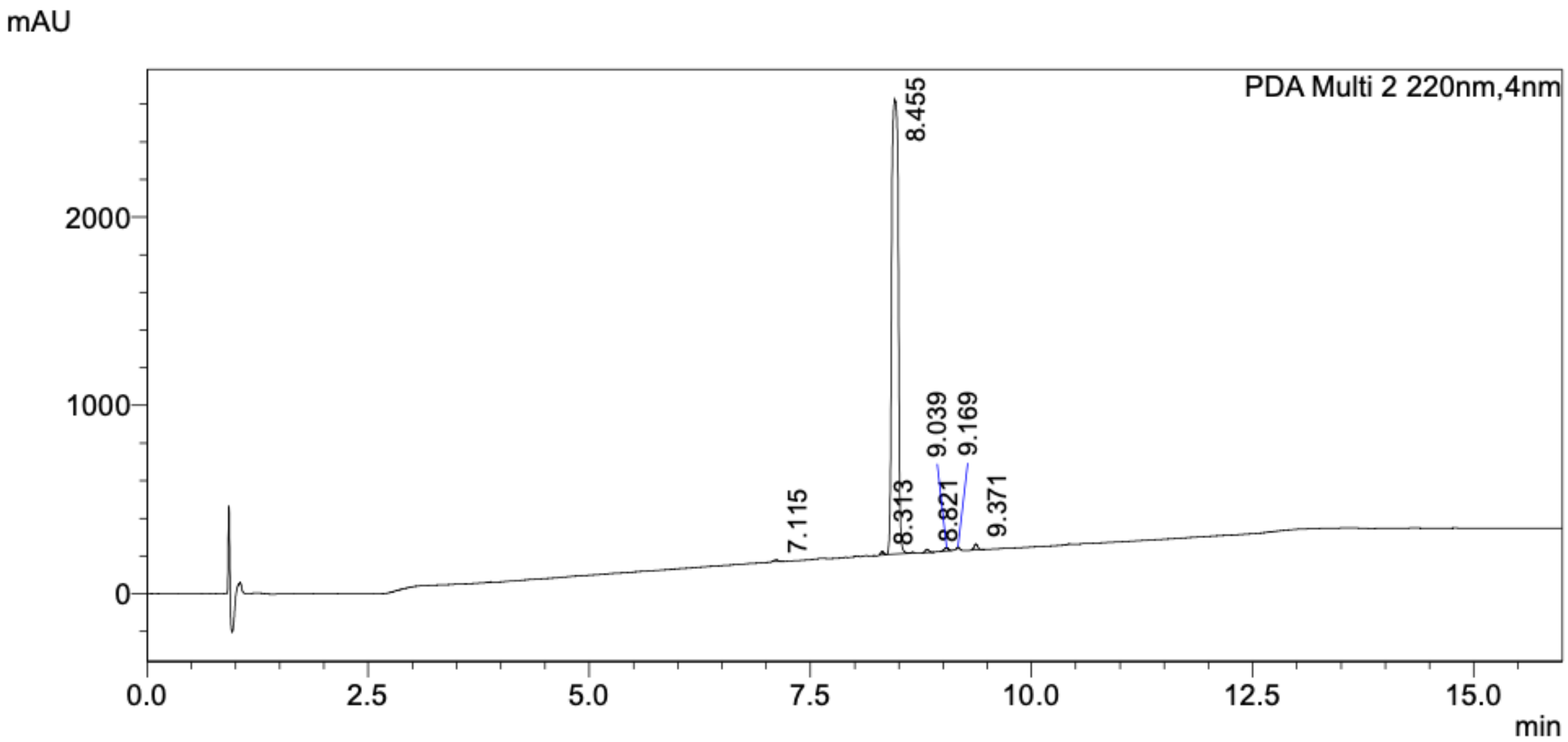

<Peak Table>

PDA Ch2 220nm

Peak#	Ret. Time	Area	Height	Unit	Mark	Area%
1	7.115	26985	8189		M	0.214
2	8.313	40875	17502		M	0.324
3	8.455	12330866	2413109		M	97.836
4	8.821	45005	16472		M	0.357
5	9.039	56478	16630		M	0.448
6	9.169	11327	6611		M	0.090
7	9.371	92064	31160		M	0.730
Total		12603599	2509673			100.000

Analysis Report

<Sample Information>

Sample Name : CFA-247(30-38)-1
Sample ID :
Data Filename : Batch purity Fatma_1162026_005.lcd
Method Filename : Fatma_Standard_5-95MeCN1minhold_10mingrad_5minhold_0.1FA_1mlmin.lcm
Batch Filename : Batch purity Fatma.lcb
Vial # : 1-29 Sample Type : Unknown
Injection Volume : 1 uL
Date Acquired : 1/16/2026 12:21:38 PM Acquired by : System Administrator
Date Processed : 1/16/2026 12:54:40 PM Processed by : System Administrator

<Chromatogram>

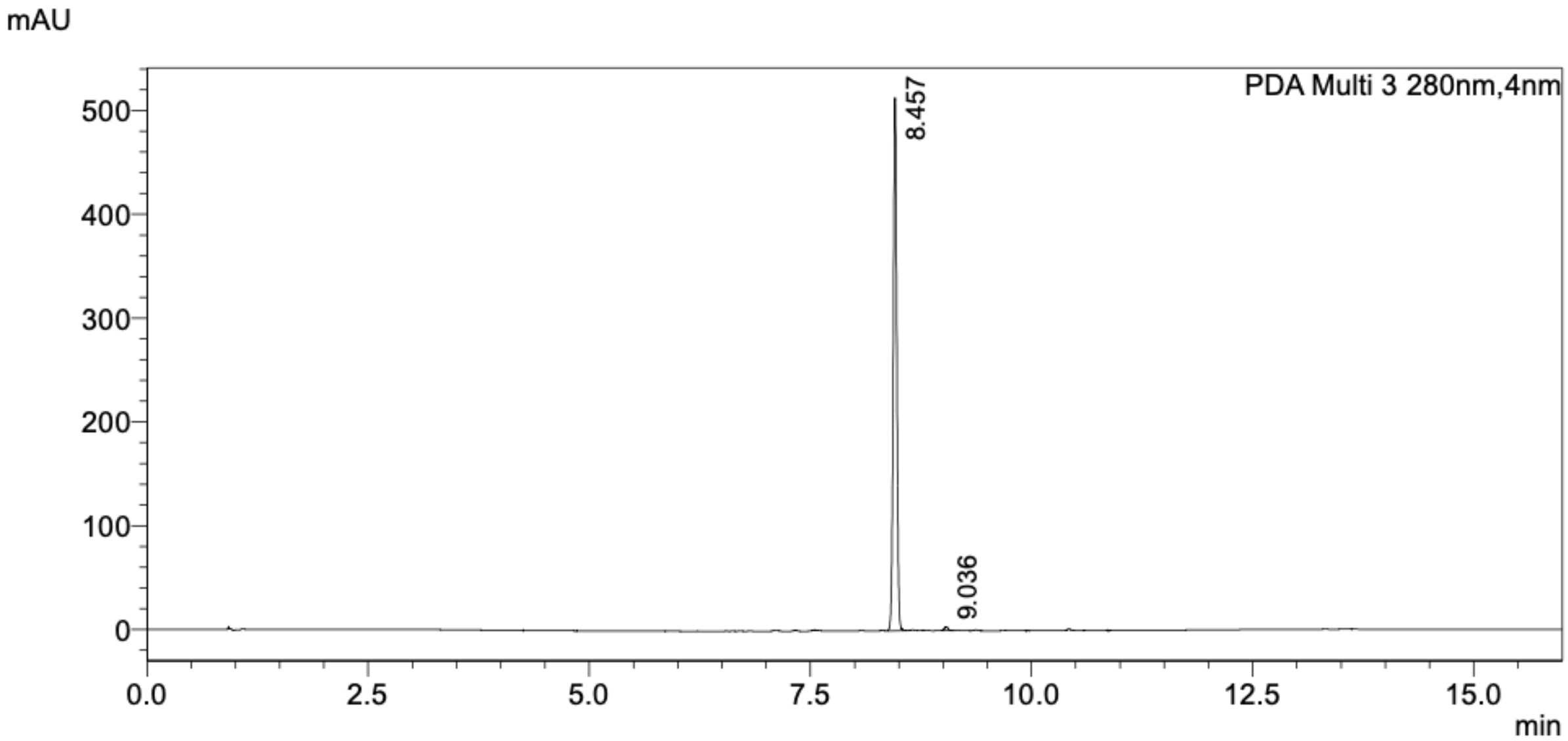

<Peak Table>

PDA Ch3 280nm

Peak#	Ret. Time	Area	Height	Unit	Mark	Area%
1	8.457	1417807	495276		M	99.267
2	9.036	10473	3593		M	0.733
Total		1428280	498869			100.000

HPLC traces for 2-(1-benzyl-2,6-dioxopiperidin-3-yl)-5-(*tert*-butyl)isoindoline-1,3-dione (7)

SHIMADZU LabSolutions **Analysis Report**

<Sample Information>

Sample Name : CFA-257
Sample ID :
Data Filename : Batch purity Fatma_1282026_002.lcd
Method Filename : Fatma_Standard_5-95MeCN1minhold_10mingrad_5minhold_0.1FA_1mlmin.lcm
Batch Filename : Batch purity Fatma.lcb
Vial # : 1-29 Sample Type : Unknown
Injection Volume : 1 uL
Date Acquired : 1/28/2026 5:00:20 PM Acquired by : System Administrator
Date Processed : 1/29/2026 10:19:01 AM Processed by : System Administrator

<Chromatogram>

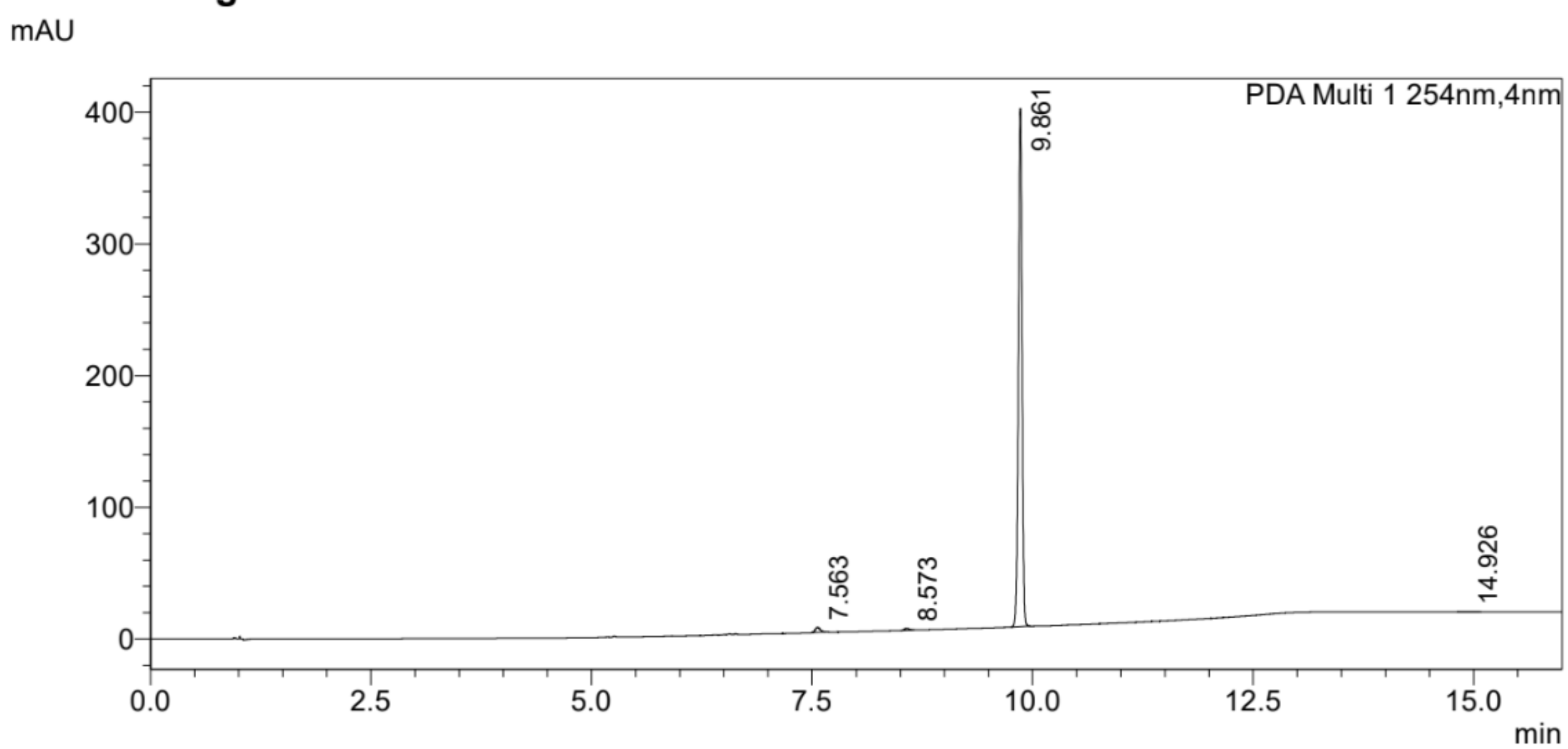

<Peak Table>

PDA Ch1 254nm

Peak#	Ret. Time	Area	Height	Unit	Mark	Area%
1	7.563	15194	3870		M	1.276
2	8.573	4832	1460		M	0.406
3	9.861	1169259	391034		M	98.215
4	14.926	1228	217			0.103
Total		1190512	396581			100.000

Analysis Report

<Sample Information>

Sample Name	: CFA-257		
Sample ID	:		
Data Filename	: Batch purity Fatma_1282026_002.lcd		
Method Filename	: Fatma_Standard_5-95MeCN1minhold_10mingrad_5minhold_0.1FA_1mlmin.lcm		
Batch Filename	: Batch purity Fatma.lcb		
Vial #	: 1-29	Sample Type	: Unknown
Injection Volume	: 1 uL		
Date Acquired	: 1/28/2026 5:00:20 PM	Acquired by	: System Administrator
Date Processed	: 1/29/2026 10:19:01 AM	Processed by	: System Administrator

<Chromatogram>

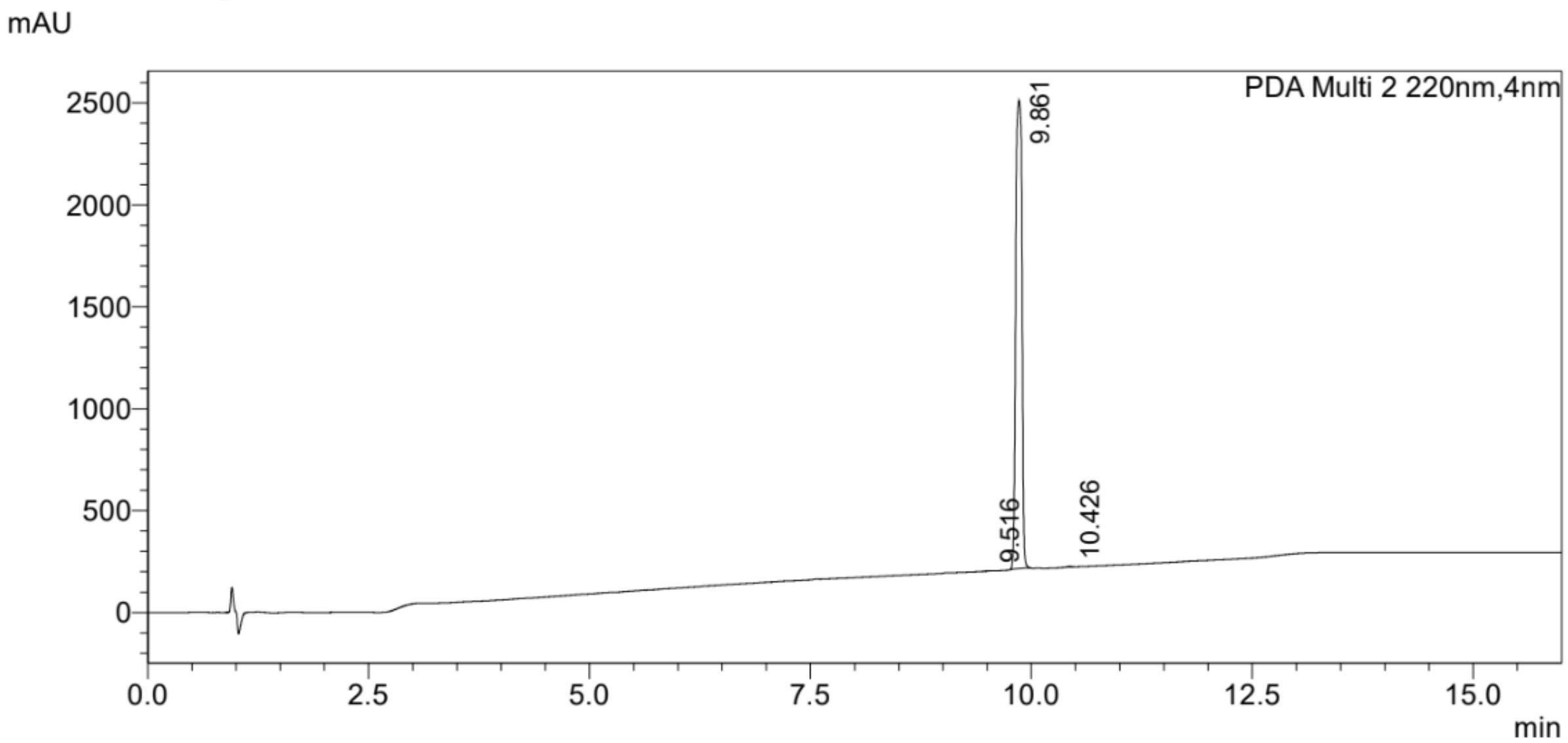

<Peak Table>

PDA Ch2 220nm

Peak#	Ret. Time	Area	Height	Unit	Mark	Area%
1	9.516	5450	2101		M	0.048
2	9.861	11290268	2292673		M	99.913
3	10.426	4329	2199		M	0.038
Total		11300047	2296973			100.000

Analysis Report

<Sample Information>

Sample Name	: CFA-257		
Sample ID	:		
Data Filename	: Batch purity Fatma_1282026_002.lcd		
Method Filename	: Fatma_Standard_5-95MeCN1minhold_10mingrad_5minhold_0.1FA_1mlmin.lcm		
Batch Filename	: Batch purity Fatma.lcb		
Vial #	: 1-29	Sample Type	: Unknown
Injection Volume	: 1 uL		
Date Acquired	: 1/28/2026 5:00:20 PM	Acquired by	: System Administrator
Date Processed	: 1/29/2026 10:19:01 AM	Processed by	: System Administrator

<Chromatogram>

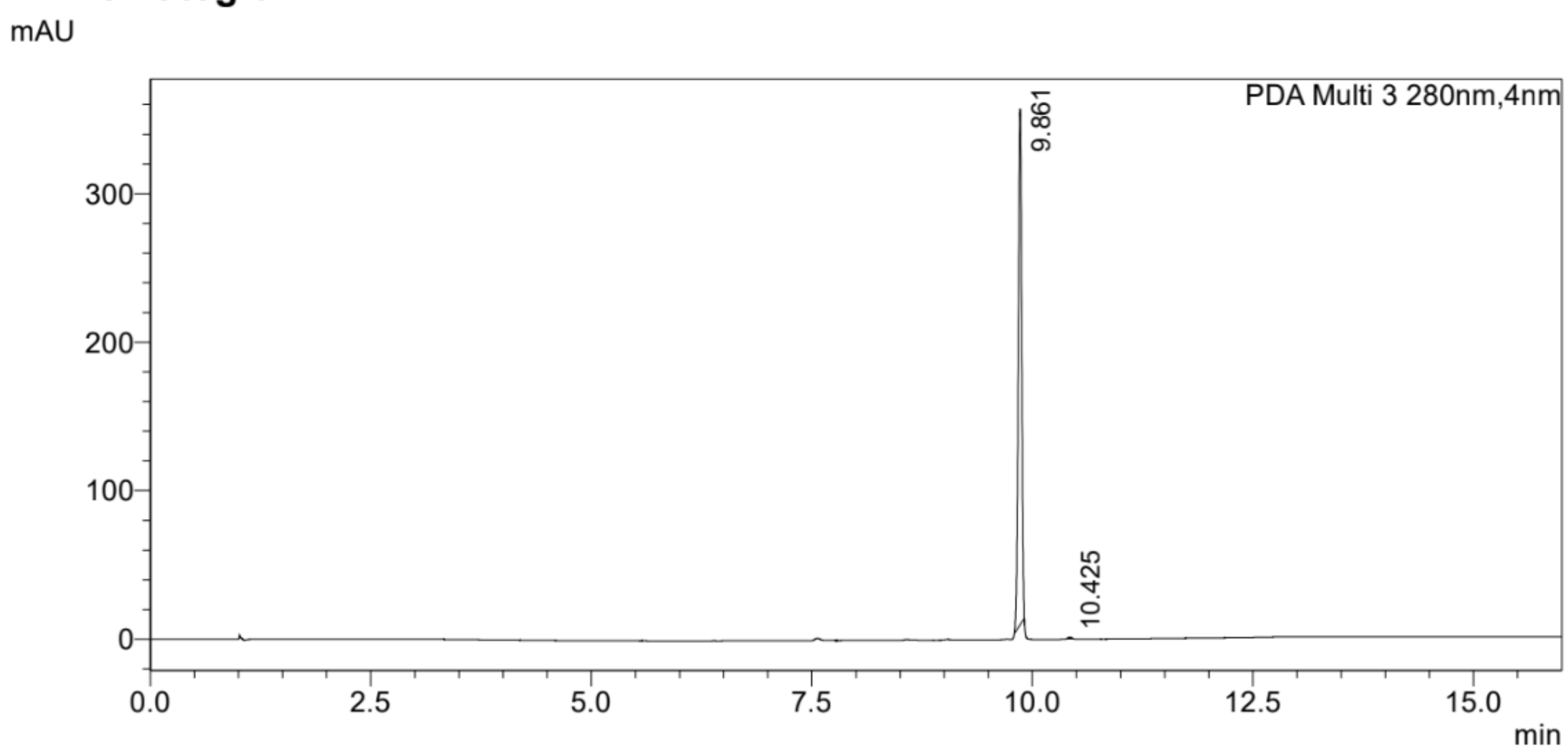

<Peak Table>

PDA Ch3 280nm

Peak#	Ret. Time	Area	Height	Unit	Mark	Area%
1	9.861	958985	344939		M	99.731
2	10.425	2584	1051		M	0.269
Total		961569	345990			100.000

HPLC traces for 5-(cyclopropylamino)-2-(2,6-dioxopiperidin-3-yl)isoindoline-1,3-dione (8)

SHIMADZU LabSolutions **Analysis Report**

<Sample Information>

Sample Name	: CFA-154		
Sample ID	:		
Data Filename	: Batch purity Fatma_9222025_003.lcd		
Method Filename	: Fatma_Standard_5-95MeCN1minhold_10mingrad_5minhold_0.1FA_1mlmin.lcm		
Batch Filename	: Batch purity Fatma.lcb		
Vial #	: 1-21	Sample Type	: Unknown
Injection Volume	: 1 uL		
Date Acquired	: 9/22/2025 10:17:18 AM	Acquired by	: System Administrator
Date Processed	: 9/22/2025 10:39:12 AM	Processed by	: System Administrator

<Chromatogram>

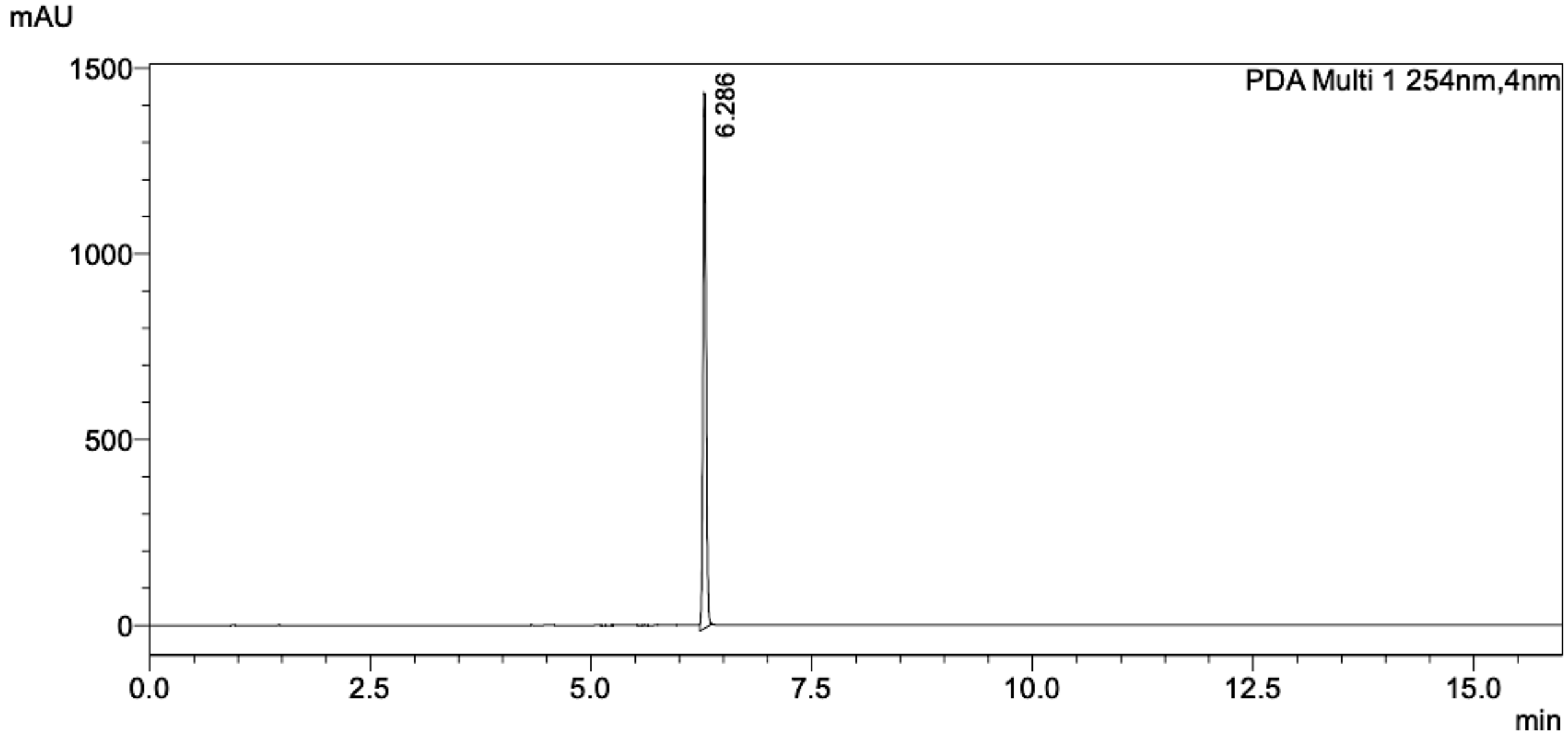

<Peak Table>

PDA Ch1 254nm

Peak#	Ret. Time	Area	Height	Unit	Mark	Area%
1	6.286	3333994	1420097		M	100.000
Total		3333994	1420097			100.000

Analysis Report

SHIMADZU LabSolutions

<Sample Information>

Sample Name	: CFA-154		
Sample ID	:		
Data Filename	: Batch purity Fatma_9222025_003.lcd		
Method Filename	: Fatma_Standard_5-95MeCN1minhold_10mingrad_5minhold_0.1FA_1mlmin.lcm		
Batch Filename	: Batch purity Fatma.lcb		
Vial #	: 1-21	Sample Type	: Unknown
Injection Volume	: 1 uL		
Date Acquired	: 9/22/2025 10:17:18 AM	Acquired by	: System Administrator
Date Processed	: 9/22/2025 10:39:12 AM	Processed by	: System Administrator

<Chromatogram>

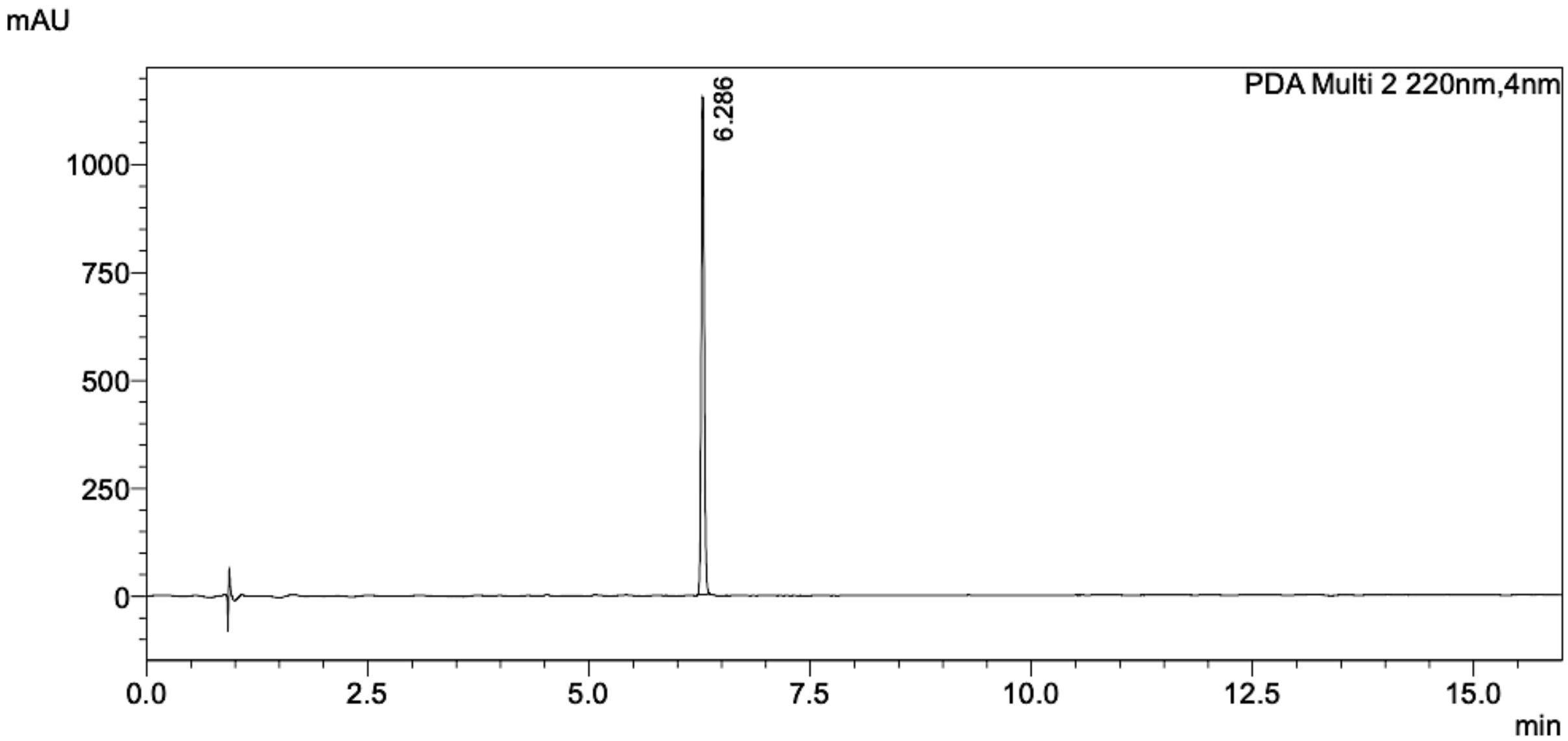

<Peak Table>

PDA Ch2 220nm

Peak#	Ret. Time	Area	Height	Unit	Mark	Area%
1	6.286	2661346	1136540		M	100.000
Total		2661346	1136540			100.000

Analysis Report

<Sample Information>

Sample Name	: CFA-154		
Sample ID	:		
Data Filename	: Batch purity Fatma_9222025_003.lcd		
Method Filename	: Fatma_Standard_5-95MeCN1minhold_10mingrad_5minhold_0.1FA_1mlmin.lcm		
Batch Filename	: Batch purity Fatma.lcb		
Vial #	: 1-21	Sample Type	: Unknown
Injection Volume	: 1 uL		
Date Acquired	: 9/22/2025 10:17:18 AM	Acquired by	: System Administrator
Date Processed	: 9/22/2025 10:39:12 AM	Processed by	: System Administrator

<Chromatogram>

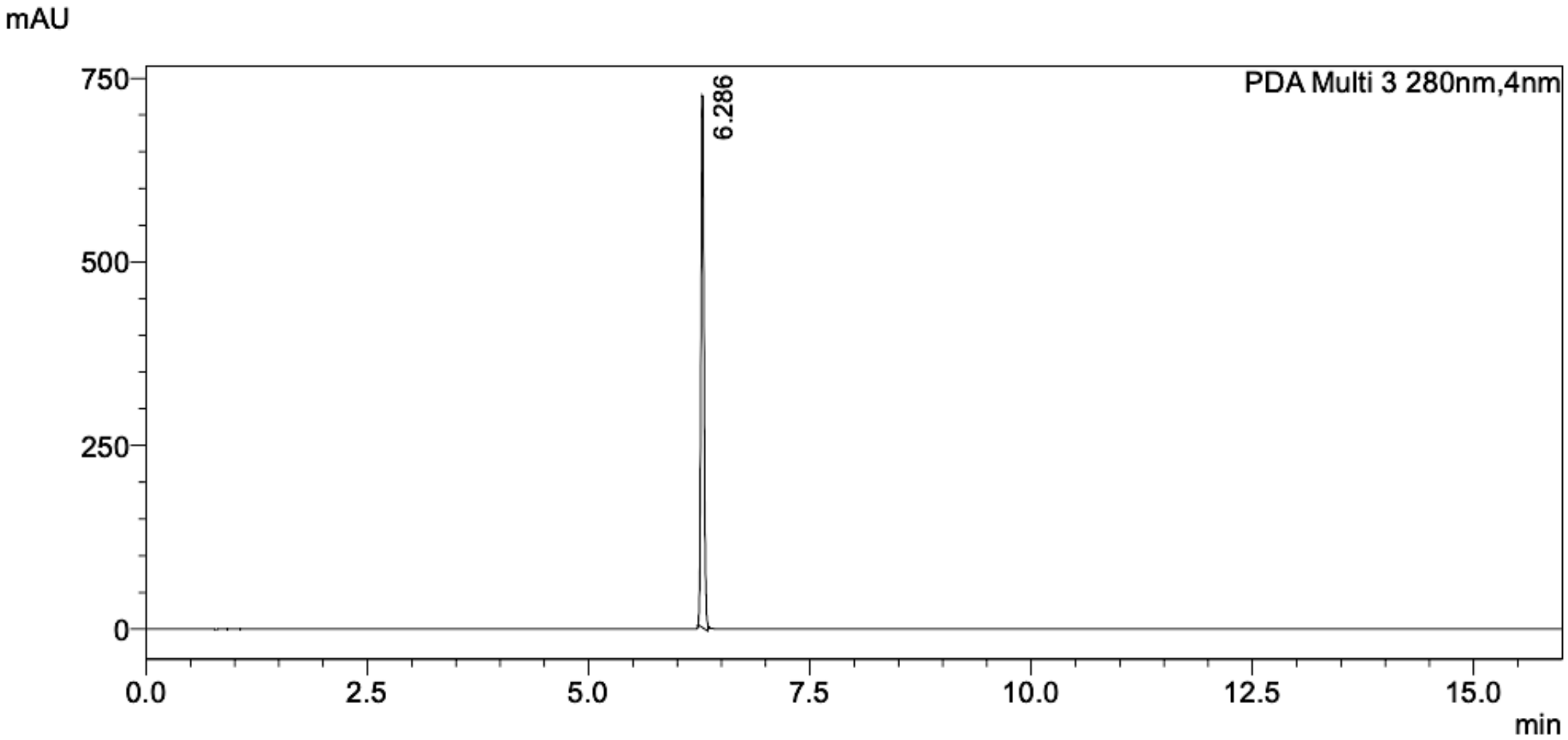

<Peak Table>

PDA Ch3 280nm

Peak#	Ret. Time	Area	Height	Unit	Mark	Area%
1	6.286	1695359	714396		M	100.000
Total		1695359	714396			100.000

HPLC traces for 4-(cyclopropylamino)-2-(2,6-dioxopiperidin-3-yl)isoindoline-1,3-dione (9)

SHIMADZU LabSolutions **Analysis Report**

<Sample Information>

Sample Name : LYL-29
Sample ID :
Data Filename : Batch purity_9222025_002.lcd
Method Filename : Fatma_Standard_5-95MeCN1minhold_10mingrad_5minhold_0.1FA_1mlmin.lcm
Batch Filename : Batch purity.lcb
Vial # : 1-51 Sample Type : Unknown
Injection Volume : 1 uL
Date Acquired : 9/22/2025 2:49:03 PM Acquired by : System Administrator
Date Processed : 9/22/2025 4:13:22 PM Processed by : System Administrator

<Chromatogram>

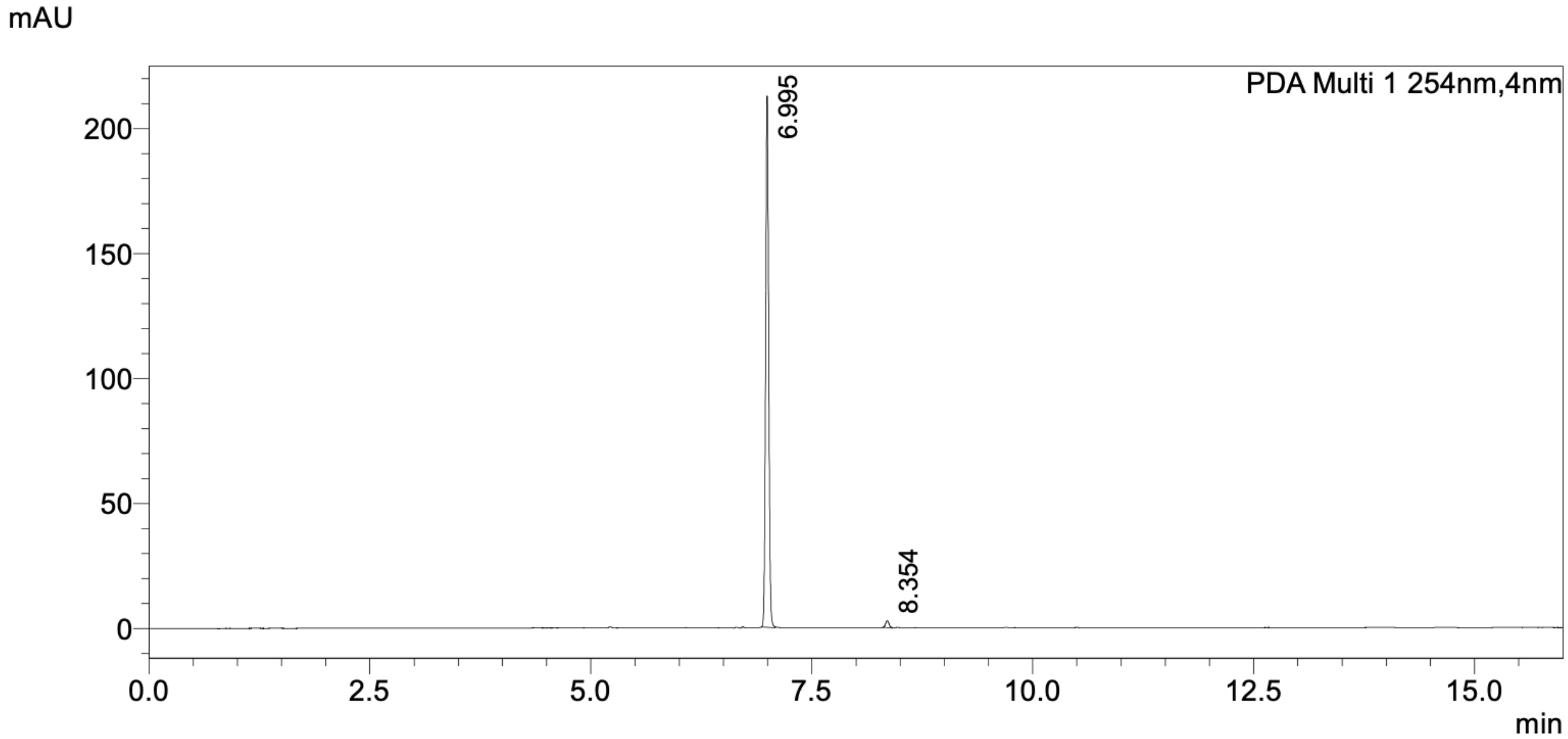

<Peak Table>

PDA Ch1 254nm

Peak#	Ret. Time	Area	Height	Unit	Mark	Area%
1	6.995	509179	208528		M	98.568
2	8.354	7397	2682		M	1.432
Total		516576	211210			100.000

Analysis Report

<Sample Information>

Sample Name	: LYL-29		
Sample ID	:		
Data Filename	: Batch purity_9222025_002.lcd		
Method Filename	: Fatma_Standard_5-95MeCN1minhold_10mingrad_5minhold_0.1FA_1mlmin.lcm		
Batch Filename	: Batch purity.lcb		
Vial #	: 1-51	Sample Type	: Unknown
Injection Volume	: 1 uL		
Date Acquired	: 9/22/2025 2:49:03 PM	Acquired by	: System Administrator
Date Processed	: 9/22/2025 4:13:22 PM	Processed by	: System Administrator

<Chromatogram>

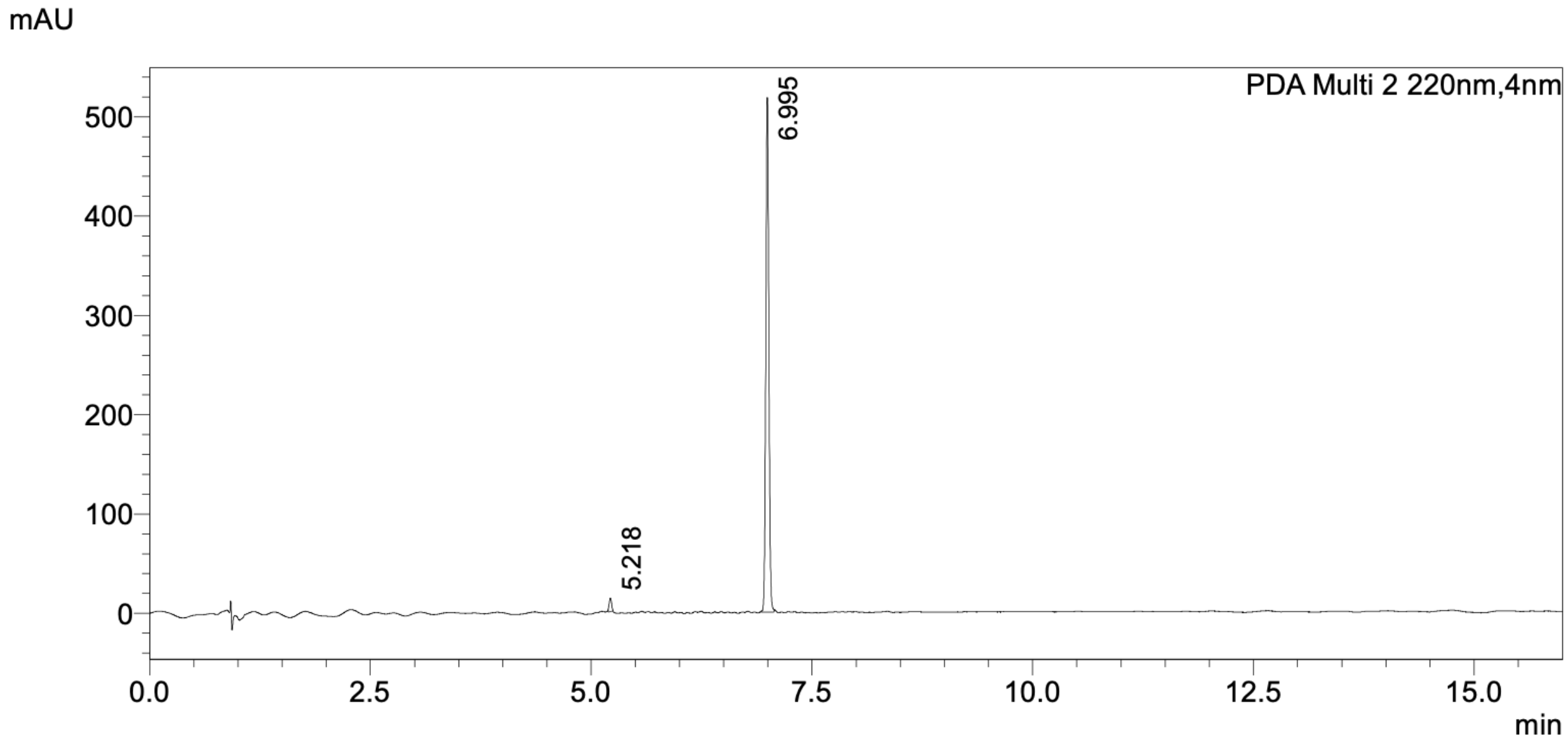

<Peak Table>

PDA Ch2 220nm

Peak#	Ret. Time	Area	Height	Unit	Mark	Area%
1	5.218	26088	13011		M	2.062
2	6.995	1239135	507703		M	97.938
Total		1265223	520714			100.000

SHIMADZU LabSolutions

Analysis Report

<Sample Information>

Sample Name	: LYL-29		
Sample ID	:		
Data Filename	: Batch purity_9222025_002.lcd		
Method Filename	: Fatma_Standard_5-95MeCN1minhold_10mingrad_5minhold_0.1FA_1mlmin.lcm		
Batch Filename	: Batch purity.lcb		
Vial #	: 1-51	Sample Type	: Unknown
Injection Volume	: 1 uL		
Date Acquired	: 9/22/2025 2:49:03 PM	Acquired by	: System Administrator
Date Processed	: 9/22/2025 4:13:22 PM	Processed by	: System Administrator

<Chromatogram>

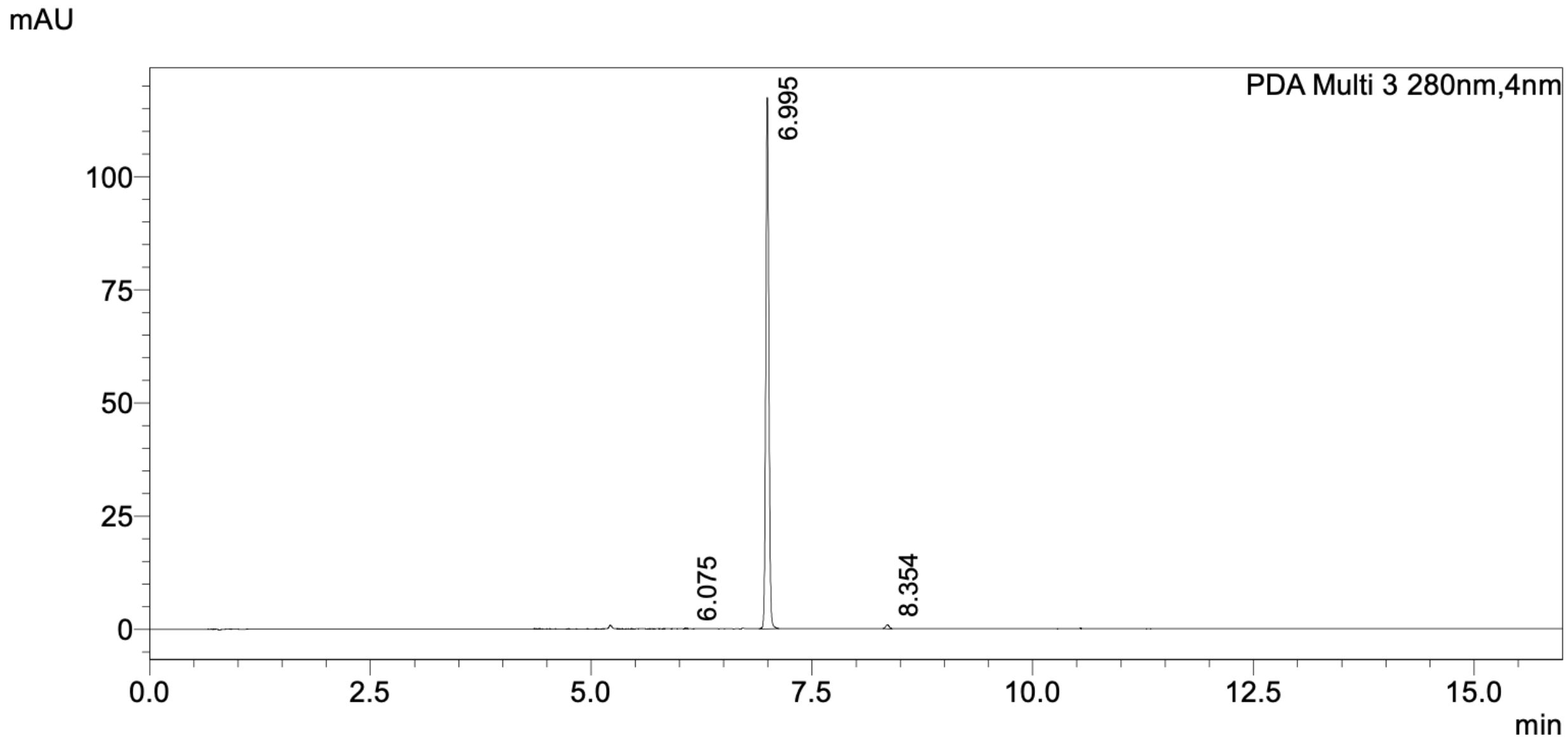

<Peak Table>

PDA Ch3 280nm

Peak#	Ret. Time	Area	Height	Unit	Mark	Area%
1	6.075	417	218		M	0.146
2	6.995	283397	115063		M	99.023
3	8.354	2378	857		M	0.831
Total		286192	116138			100.000

REFERENCES

(1) Sievers, Q. L.; Petzold, G.; Bunker, R. D.; Renneville, A.; Słabicki, M.; Liddicoat, B. J.; Abdulrahman, W.; Mikkelsen, T.; Ebert, B. L.; Thomä, N. H. Defining the Human C2H2 Zinc Finger Degrome Targeted by Thalidomide Analogs through CRBN. *Science* **2018**, *362* (6414), eaat0572. https://doi.org/10.1126/SCIENCE.AAT0572.

(2) Fulmer, G. R.; Miller, A. J. M.; Sherden, N. H.; Gottlieb, H. E.; Nudelman, A.; Stoltz, B. M.; Bercaw, J. E.; Goldberg, K. I. NMR Chemical Shifts of Trace Impurities: Common Laboratory Solvents, Organics, and Gases in Deuterated Solvents Relevant to the Organometallic Chemist. *Organometallics* **2010**, *29* (9), 2176–2179. https://doi.org/10.1021/OM100106E/SUPPL_FILE/OM100106E_SI_001.PDF.

(3) Burslem, G. M.; Ottis, P.; Jaime-Figueroa, S.; Morgan, A.; Cromm, P. M.; Toure, M.; Crews, C. M. Efficient Synthesis of Immunomodulatory Drug Analogues Enables Exploration of Structure-Degradation Relationships. *ChemMedChem* **2018**, *13* (15), 1508–1512. https://doi.org/10.1002/CMDC.201800271.

(4) Jochem, M.; Schrempf, A.; Wagner, L.-M.; Segal, D.; Cisneros, J.; Ng, A.; Winter, G. E.; Krijgsveld, J. Degradome Analysis to Identify Direct Protein Substrates of Small-Molecule Degraders. *Cell Chemical Biology* **2025**, *32* (1), 192-200.e6. https://doi.org/10.1016/j.chembiol.2024.10.007.

(5) Liu, H.; Naismith, J. H. An Efficient One-Step Site-Directed Deletion, Insertion, Single and Multiple-Site Plasmid Mutagenesis Protocol. *BMC Biotechnol* **2008**, *8*, 91. https://doi.org/10.1186/1472-6750-8-91.

(6) Yi, S. A.; Sepic, S.; Schulman, B. A.; Ordureau, A.; An, H. mTORC1-CTLH E3 Ligase Regulates the Degradation of HMG-CoA Synthase 1 through the Pro/N-Degron Pathway. *Mol Cell* **2024**, *84* (11), 2166-2184.e9. https://doi.org/10.1016/j.molcel.2024.04.026.

(7) Brennan, P. J.; Saunders, R. E.; Spanou, M.; Singleton, S. E.; Serafini, M.; Sun, L.; Heger, G. P.; Konopacka, A.; Beveridge, R. D.; Taylor, C. C.; DePaola, P. I.; Gordon, L.; Bunally, S. B.; Saudemont, A.; Benowitz, A. B.; Martinez-Fleites, C.; Schmitt, D. L.; Damoiseaux, R.; Queisser, M. A.; An, H.; Deane, C. M.; Hann, M. M.; Brayshaw, L. L.; Conway, S. J. Orthogonal IMiD-Degron Pairs Induce Selective Protein Degradation in Cells. *ACS Chem. Biol.* **2025**. https://doi.org/10.1021/acschembio.5c00751.

(8) Wang, Y.; Yang, F.; Gritsenko, M. A.; Wang, Y.; Clauss, T.; Liu, T.; Shen, Y.; Monroe, M. E.; Lopez-Ferrer, D.; Reno, T.; Moore, R. J.; Klemke, R. L.; Camp, D. G.; Smith, R. D. Reversed-Phase Chromatography with Multiple Fraction Concatenation Strategy for Proteome Profiling of Human MCF10A Cells. *Proteomics* **2011**, *11* (10), 2019–2026. https://doi.org/10.1002/pmic.201000722.

(9) Paulo, J. A.; O’Connell, J. D.; Everley, R. A.; O’Brien, J.; Gygi, M. A.; Gygi, S. P. Quantitative Mass Spectrometry-Based Multiplexing Compares the Abundance of 5000 S. Cerevisiae Proteins across 10 Carbon Sources. *J. Proteomics* **2016**, *148*, 85–93. https://doi.org/10.1016/j.jprot.2016.07.005.

(10) McAlister, G. C.; Nusinow, D. P.; Jedrychowski, M. P.; Wühr, M.; Huttlin, E. L.; Erickson, B. K.; Rad, R.; Haas, W.; Gygi, S. P. MultiNotch MS3 Enables Accurate, Sensitive, and Multiplexed Detection of Differential Expression across Cancer Cell Line Proteomes. *Anal. Chem.* **2014**, *86* (14), 7150–7158. https://doi.org/10.1021/ac502040v.

(11) Paulo, J. A.; O'Connell, J. D.; Gygi, S. P. A Triple Knockout (TKO) Proteomics Standard for Diagnosing Ion Interference in Isobaric Labeling Experiments. *J. Am. Soc. Mass. Spectrom.* **2016**, *27* (10), 1620–1625. https://doi.org/10.1007/s13361-016-1434-9.

(12) Schweppe, D. K.; Prasad, S.; Belford, M. W.; Navarrete-Perea, J.; Bailey, D. J.; Huguet, R.; Jedrychowski, M. P.; Rad, R.; McAlister, G.; Abbatiello, S. E.; Woulters, E. R.; Zabrouskov, V.; Dunyach, J.-J.; Paulo, J. A.; Gygi, S. P. Characterization and Optimization of Multiplexed Quantitative Analyses Using High-Field Asymmetric-Waveform Ion Mobility Mass Spectrometry. *Anal Chem* **2019**, *91* (6), 4010–4016. https://doi.org/10.1021/acs.analchem.8b05399.

(13) Schweppe, D. K.; Eng, J. K.; Yu, Q.; Bailey, D.; Rad, R.; Navarrete-Perea, J.; Huttlin, E. L.; Erickson, B. K.; Paulo, J. A.; Gygi, S. P. Full-Featured, Real-Time Database Searching Platform Enables Fast and Accurate Multiplexed Quantitative Proteomics. *J. Proteome. Res.* **2020**, *19* (5), 2026–2034. https://doi.org/10.1021/acs.jproteome.9b00860.

(14) Erickson, B. K.; Mintseris, J.; Schweppe, D. K.; Navarrete-Perea, J.; Erickson, A. R.; Nusinow, D. P.; Paulo, J. A.; Gygi, S. P. Active Instrument Engagement Combined with a Real-Time Database Search for Improved Performance of Sample Multiplexing Workflows. *J. Proteome. Res.* **2019**, *18* (3), 1299–1306. https://doi.org/10.1021/acs.jproteome.8b00899.

(15) Eng, J. K.; Hoopmann, M. R.; Jahan, T. A.; Egertson, J. D.; Noble, W. S.; MacCoss, M. J. A Deeper Look into Comet--Implementation and Features. *J Am Soc Mass Spectrom* **2015**, *26* (11), 1865–1874. https://doi.org/10.1007/s13361-015-1179-x.

(16) Eng, J. K.; Jahan, T. A.; Hoopmann, M. R. Comet: An Open-Source MS/MS Sequence Database Search Tool. *Proteomics* **2013**, *13* (1), 22–24. https://doi.org/10.1002/pmic.201200439.

(17) Rad, R.; Li, J.; Mintseris, J.; O'Connell, J.; Gygi, S. P.; Schweppe, D. K. Improved Monoisotopic Mass Estimation for Deeper Proteome Coverage. *J. Proteome. Res.* **2021**, *20* (1), 591–598. https://doi.org/10.1021/acs.jproteome.0c00563.

(18) Elias, J. E.; Gygi, S. P. Target-Decoy Search Strategy for Increased Confidence in Large-Scale Protein Identifications by Mass Spectrometry. *Nat. Methods* **2007**, *4* (3), 207–214. https://doi.org/10.1038/nmeth1019.

(19) Huttlin, E. L.; Jedrychowski, M. P.; Elias, J. E.; Goswami, T.; Rad, R.; Beausoleil, S. A.; Villén, J.; Haas, W.; Sowa, M. E.; Gygi, S. P. A Tissue-Specific Atlas of Mouse Protein Phosphorylation and Expression. *Cell* **2010**, *143* (7), 1174–1189. https://doi.org/10.1016/j.cell.2010.12.001.

(20) Savitski, M. M.; Wilhelm, M.; Hahne, H.; Kuster, B.; Bantscheff, M. A Scalable Approach for Protein False Discovery Rate Estimation in Large Proteomic Data Sets. *Mol. Cell. Proteomics.* **2015**, *14* (9), 2394–2404. https://doi.org/10.1074/mcp.M114.046995.

(21) Tyanova, S.; Temu, T.; Sinitcyn, P.; Carlson, A.; Hein, M. Y.; Geiger, T.; Mann, M.; Cox, J. The Perseus Computational Platform for Comprehensive Analysis of (Prote)Omics Data. *Nat. Methods.* **2016**, *13* (9), 731–740. https://doi.org/10.1038/nmeth.3901.

(22) Salomon-Ferrer, R.; Case, D. A.; Walker, R. C. An Overview of the Amber Biomolecular Simulation Package. *WIREs Computational Molecular Science* **2013**, *3* (2), 198–210. https://doi.org/10.1002/wcms.1121.

(23) *UCSF Chimera--a visualization system for exploratory research and analysis - PubMed.* https://pubmed.ncbi.nlm.nih.gov/15264254/ (accessed 2024-12-05).
